# Molecular design of a therapeutic LSD analogue with reduced hallucinogenic potential

**DOI:** 10.1073/pnas.2416106122

**Published:** 2025-04-14

**Authors:** Jeremy R. Tuck, Lee E. Dunlap, Yara A. Khatib, Cassandra J. Hatzipantelis, Sammy Weiser Novak, Rachel M. Rahn, Alexis R. Davis, Adam Mosswood, Anna M. M. Vernier, Ethan M. Fenton, Isak K. Aarrestad, Robert J. Tombari, Samuel J. Carter, Zachary Deane, Yuning Wang, Arlo Sheridan, Monica A. Gonzalez, Arabo A. Avanes, Noel A. Powell, Milan Chytil, Sharon Engel, James C. Fettinger, Amaya R. Jenkins, William A. Carlezon, Alex S. Nord, Brian D. Kangas, Kurt Rasmussen, Conor Liston, Uri Manor, David E. Olson

**Affiliations:** ^a^Chemistry and Chemical Biology Graduate Program, University of California, Davis, CA 95616; ^b^Institute for Psychedelics and Neurotherapeutics, University of California, Davis, CA 95616; ^c^Pharmacology and Toxicology Graduate Program, University of California, Davis, CA 95616; ^d^Department of Chemistry, University of California, Davis, CA 95616; ^e^Waitt Advanced Biophotonics Center, Salk Institute for Biological Studies, La Jolla, CA 92037; ^f^Department of Psychiatry and Brain and Mind Research Institute, Weill Cornell Medicine, New York, NY 10065; ^g^Biochemistry, Cellular, Molecular and Developmental Biology Graduate Program, University of California, Davis, CA 95616; ^h^Neuroscience Graduate Program, University of California, Davis, CA 95618; ^i^Delix Therapeutics, Inc, Bedford, MA 01730; ^j^Jerry and Phyllis Rappaport Center of Excellence in Basic Neuroscience Research, Harvard Medical School McLean Hospital, Belmont, MA 02478; ^k^Department of Psychiatry and Behavioral Sciences, School of Medicine University of CaliforniaDavis, CA 95817; ^l^Department of Neurobiology, Physiology, and Behavior University of California, Davis, CA 95616; ^m^Center for Neuroscience, University of California, Davis, CA 95618; ^n^Department of Cell & Developmental Biology, School of Biological Sciences, University of California, San Diego, CA 92093; ^o^Department of Biochemistry & Molecular Medicine, School of Medicine, University of California, Davis, Sacramento, CA 95817

**Keywords:** psychedelic, neuroplasticity, LSD, neuropsychiatric disease, nonhallucinogenic

## Abstract

Decreased dendritic spine density in the cortex is a key pathological feature of neuropsychiatric diseases including depression, addiction, and schizophrenia (SCZ). Psychedelics possess a remarkable ability to promote cortical neuron growth and increase spine density; however, these compounds are contraindicated for patients with SCZ or a family history of psychosis. Here, we report the molecular design and de novo total synthesis of (+)-JRT, a structural analogue of lysergic acid diethylamide (LSD) with lower hallucinogenic potential and potent neuroplasticity-promoting properties. In addition to promoting spinogenesis in the cortex, (+)-JRT produces therapeutic effects in behavioral assays relevant to depression and cognition without exacerbating behavioral and gene expression signatures relevant to psychosis. This work underscores the potential of nonhallucinogenic psychoplastogens for treating diseases where the use of psychedelics presents significant safety concerns.

## Significance

Psychedelic compounds, such as lysergic acid diethylamide (LSD), can promote the growth of atrophied cortical neurons, which is relevant to the treatment of numerous brain conditions. However, their hallucinogenic properties have limited their adoption as medicines and preclude their use in certain patient populations, such as those with schizophrenia or a family history of psychosis. By transposing only two atoms, we have created JRT, an exceptionally potent analogue of LSD with lower hallucinogenic potential, improved pharmacological selectivity, and the ability to produce a wide range of therapeutic effects. Our work highlights the potential of rationally designed, nonhallucinogenic analogues of psychedelics for treating diseases where the use of psychedelics is contraindicated.

Psychoplastogens are small molecules that rapidly promote structural plasticity in the cortex and produce sustained therapeutic behavioral effects after a single administration (1). The dissociative anesthetic ketamine and serotonergic psychedelics are among the best-known psychoplastogens (2–4), though their hallucinogenic effects have limited their clinical use. More recently, nonhallucinogenic analogues of psychedelics have been developed with potential for treating depression and substance use disorders (5–9). Several of these compounds have demonstrated the ability to promote cortical neuron growth (5, 6, 9, 10), and a single administration of the nonhallucinogenic psychedelic analogue tabernanthalog was able to repair cortical circuits damaged by chronic stress (11) and produce sustained antidepressant-like effects and reductions in drug-seeking behavior (5).

Depression and substance use disorder involve cortical dysregulation (12) and often co-occur with schizophrenia (SCZ) (13), a neuropsychiatric disease that affects approximately 0.5% of individuals (14) and is characterized by a wide range of symptoms including hallucinations and delusions (positive symptoms), anhedonia and avolition (negative symptoms), and impairments in attention and working memory (cognitive symptoms) (15). In fact, decreased dendritic arborization, reduced dendritic spine density, and lower levels of synaptic proteins in the cortex are hallmarks of SCZ (16–20), and these structural changes are believed to contribute to the positive, negative, and cognitive symptoms of the disease (21, 22, 23). While current antipsychotics have demonstrated efficacy for treating the positive symptoms of SCZ through D2 and 5-HT2A receptor antagonism (24, 25), they have proven much less effective for addressing the negative and cognitive symptoms (26), and evidence suggests that they are unlikely to rescue morphological or synaptic deficits (17, 20). Thus, compounds capable of promoting cortical neuron growth have enormous therapeutic potential for addressing these aspects of the disease. However, many compounds that promote cortical neuron growth are hallucinogens. Emergency department visits involving hallucinogens are associated with a greater risk for developing SCZ spectrum disorder (27), and the use of psychedelics has generally been precluded in patients with SCZ or related psychotic disorders.

While several psychedelic scaffolds have been used to develop nonhallucinogenic analogues, the ergoline core was particularly attractive to us given that lysergic acid diethylamide (LSD) is an exceptionally potent 5-HT2A and 5-HT2C receptor agonist with an impressive ability to promote spine growth in cortical cultures (3, 28). Here, we used rational chemical design to convert LSD into a potent psychoplastogen with reduced hallucinogenic potential, simply by transposing two atoms. Unlike LSD, this constitutional isomer could not be produced via semisynthesis from lysergic acid and required the development of an efficient de novo total synthesis. Our work establishes the indolonaphthyridine core as a scaffold for the construction of nonhallucinogenic psychoplastogens and demonstrates that exceedingly small structural modifications to hallucinogenic 5-HT2A receptor ligands can have profound functional consequences and be used to improve efficacy and safety profiles.

## Results

### Design of JRT.

**Fig. 1.**
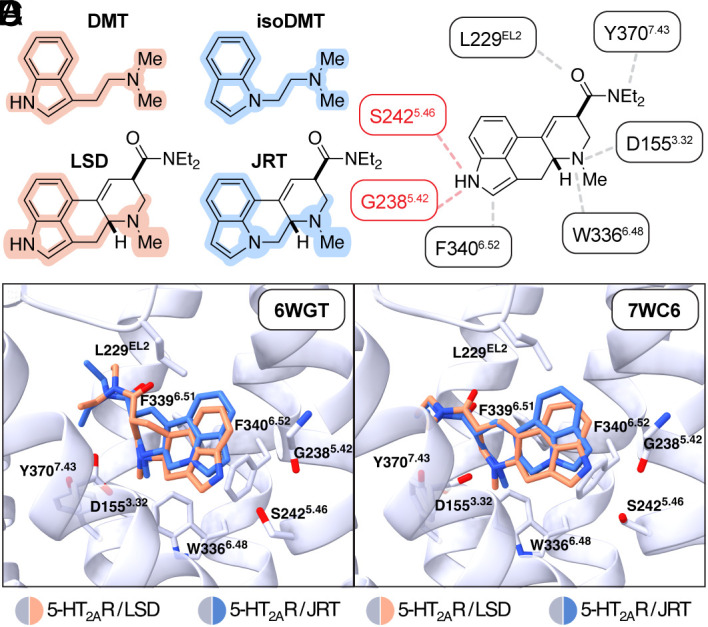
Structural basis for the rational design of JRT. (*A*) Comparison between the structures of *N,N*-dimethyltryptamine and LSD with *N,N*-dimethylisotryptamine (isoDMT) and JRT. (*B*) Structure of LSD with key 5-HT2AR contacts indicated. Key hydrogen bonding interactions between LSD and the 5-HT2AR are highlighted in red. These interactions are not possible for JRT due to its lack of an indole N–H bond. (*C* and *D*) Molecular docking of JRT into the crystal structures of the 5-HT2AR bound to LSD (PDB: 6WGT (*Left*) and 7WC6 (*Right*)) demonstrates that JRT is predicted to adopt similar binding poses as LSD with the exception that the indole nitrogen of JRT is predicted to be more removed from S242^5.46^ or G238^5.42^, respectively. The binding modes with the lowest RMSD score relative to native bound (+)-LSD are shown. Transmembrane helix 4 was removed for clarity. DMT = *N,N*-dimethyltryptamine; LSD = lysergic acid diethylamide. See also *SI Appendix*, Fig. S1.

The *N,N*-dimethyltryptamine (DMT) core structure is embedded within the tetracyclic ergoline framework of LSD (Fig. 1*A*). Previously, we demonstrated that transposition of the *N,N*-dimethylaminoethyl substituent from C3 to N1 reduced the hallucinogenic potential of tryptamines (29) while maintaining their effects on structural neuroplasticity (10). We reasoned that an analogous modification to LSD might lower its hallucinogenic potential while preserving key contacts within the 5-HT2AR binding pocket that endow LSD with exceptional potency. Specifically, our goal was to selectively disrupt the hydrogen bonding interaction between the indole N–H and S242^5.46^ or G238^5.42^ (Fig. 1*B*) while maintaining other interactions such as the salt bridge between the protonated tertiary amine and D155^3.32^, the edge-to-face π-stacking interaction between the indole and F340^6.52^, and the general hydrophobic interactions between the diethylamide and the second extracellular loop (EL2) of the protein (7, 30).

We hypothesized that by eliminating a hydrogen bonding interaction with S242^5.46^ or G238^5.42^, the 5-HT2AR would adopt a ligand-induced conformation not conducive to hallucinogenic signaling. Using psychLight2—an engineered biosensor that couples ligand-induced conformational changes to a fluorescence readout—we previously demonstrated that nonhallucinogenic ligands of the 5-HT2AR induce distinct conformational states as compared to their hallucinogenic congeners (6). Moreover, structural biology studies have revealed that differences in binding poses between hallucinogenic and nonhallucinogenic ligands can be quite subtle (7). The indolonaphthyridine constitutional isomer of LSD cannot form a hydrogen bond with S242^5.46^ or G238^5.42^, and we named this compound JRT.

Molecular docking studies indicated that JRT—which shares the same absolute and relative stereochemistry as LSD—should adopt a favorable binding pose within the 5-HT2A receptor that is very similar to that of LSD in two recently reported 5-HT2AR crystal structures (RMSD = 1.239 in 6WGT, Fig. 1*C*; RMSD = 0.930 in 7WC6, Fig. 1*D*). Docking LSD produced binding poses nearly identical to the poses reported in the respective crystal structures (*SI Appendix*, Fig. S1 *A* and *B*) (7, 30), supporting the validity of our computational modeling. Moreover, docking the enantiomer of JRT did not produce any poses with substantial homology to that of LSD (*SI Appendix*, Fig. S1 *C* and *D*). The only key difference between the docked pose of JRT and the position of LSD in the crystal structures was that the indole of JRT was shifted ~1 Å away from S242^5.46^ (Fig. 1*C* and *SI Appendix*, Fig. S1*E*) or G238^5.42^ (Fig. 1*D* and *SI Appendix*, Fig. S1*F*).

### De Novo Total Synthesis of (+)-JRT.

**Fig. 2.**
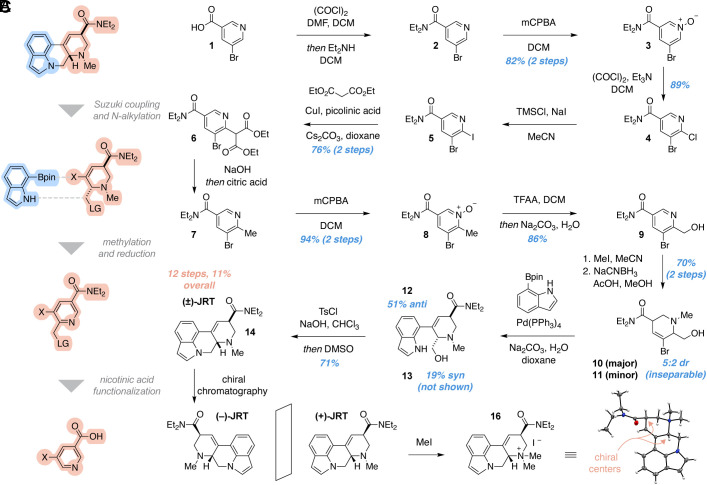
Total synthesis of JRT. (*A*) Retrosynthetic analysis indicates that JRT can be accessed from a 7-substituted indole and a 3-substituted nicotinic acid. (*B*) The total synthesis of (±)-JRT was completed in 12 steps and 11% overall yield. (*C*) X-ray crystal structure of methylated (+)-JRT (16). The counterion (iodide) and a solvent molecule (DCM) in the crystal structure were removed for clarity. red = oxygen, blue = nitrogen, gray = carbon, white = hydrogen. See also *SI Appendix*, Fig. S2.

We envisioned that JRT could be accessed via Suzuki coupling and subsequent *N*-alkylation of a commercially available indole boronic ester with a bifunctional tetrahydropyridine intermediate (Fig. 2*A*). The latter compound could be produced through methylation and regioselective reduction of a highly functionalized nicotinic acid derivative. The viability of this retrosynthetic approach relied on being able to readily access a key 2,3,5-trisubstituted pyridine intermediate.

We began our total synthesis of JRT by converting commercially available 5-bromonicotinic acid **1** into the *N,N*-diethylnicotinamide **2** (Fig. 2*B*), which was used in the subsequent reaction without purification. Oxidation of **2** proceeded smoothly in the presence of mCPBA to give the corresponding *N*-oxide **3** in 82% yield over 2 steps. A site-selective chlorination (31) of **3** was accomplished in high yield (89%) using oxalyl chloride and triethylamine. Next, we used a mixture of trimethylsilyl chloride and sodium iodide to facilitate chloride/iodide exchange (32), yielding a ~9:1 mixture of **5** and **4**, respectively. After extraction, unpurified **5** was subjected to a copper(I) iodide catalyzed coupling reaction with diethyl malonate (33), to afford **6** in 76% yield over 2 steps.

From **6**, successive decarboxylation events afforded the substituted picoline **7**. The malonate substituent was efficiently hydrolyzed and monodecarboxylated under basic conditions, while the second decarboxylation only proceeded after adjustment to a mildly acidic pH using aqueous citric acid (34). The picoline **7** was sufficiently pure after extraction to undergo mCPBA-mediated oxidation to its corresponding *N*-oxide, affording **8** in 94% yield over this sequence. Subsequent Boekelheide rearrangement (35) and hydrolysis of the resulting trifluoroacetate converted **8** to alcohol **9** in 86% yield.

Alkylation of **9** with methyl iodide afforded the corresponding pyridinium salt, which was readily reduced to an inseparable mixture of tetrahydropyridines **10** and **11** using NaCNBH_3_ in acidic methanol (70% combined yield over 2 steps, 5:2 dr). The mixture was subjected to Suzuki coupling with indole-7-boronic acid pinacol ester to yield a separable mixture of anti (**12**, 51%) and syn (**13**, 19%) diastereomers. Given that the cross-coupling was performed under basic conditions, we tentatively assigned **12** and **13** as the anti and syn diastereomers, respectively. Related tetrahydropyridines, such as LSD, are known to favor the more thermodynamically stable anti-isomers when allowed to equilibrate under basic conditions (36). This stereochemical assignment was later confirmed via X-ray crystallography upon completion of the total synthesis (Fig. 2 *B* and *C*).

Next, we envisioned that activation of the primary alcohol followed by indole *N*-alkylation would afford (±)-JRT (14). Tosylation emerged as the most viable option for this transformation, as several standard conditions for converting the alcohol to an alkyl halide also resulted in C3-functionalization of the indole. Interestingly, treatment of **12** with tosyl chloride (TsCl) and triethylamine in dichloromethane did not yield the corresponding tosylate. Instead, the alkyl chloride was produced in near quantitative yields and this intermediate was prone to decomposition. We subsequently found that the use of hydroxide bases favored the tosylate while suppressing the formation of the alkyl chloride. Treatment of **12** with TsCl and NaOH in chloroform, followed by the addition of DMSO as a cosolvent, facilitated a highly selective *O*-tosylation and rapid ring closure in a single step to yield (±)-JRT (14) in 71% yield. We were also able to convert the syn diastereomer **13** to **15** under identical conditions (see SI for details), albeit in lower yields due to in situ epimerization. However, we were able to take advantage of this epimerization reaction to convert **15** into **14**. Treating a 1:2 mixture of **14**:**15** with base led to the rapid establishment of a 5:1 thermodynamic ratio favoring **14** (*SI Appendix*, Fig. S2). Using chiral HPLC, we separated (±)-JRT (14) into its respective enantiomers. Methylation of (+)-JRT produced **16**, which enabled us to confirm that the absolute stereochemistry of (+)-JRT was the same as (+)-LSD (Fig. 2*C*). Finally, we converted both (+)-JRT and (–)-JRT into their corresponding fumarate salts (1:1) in preparation for biological evaluation.

### (+)-JRT Is Highly Selective for Serotonin Receptors.

**Fig. 3.**
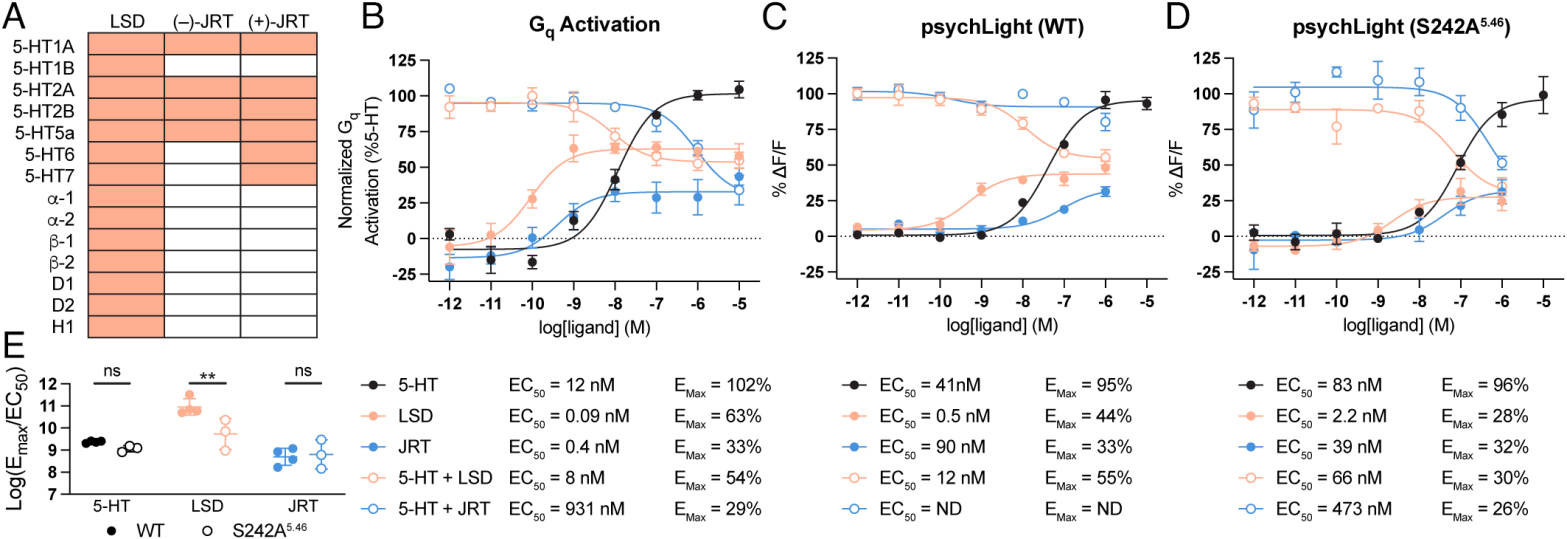
(+)-JRT is highly selective for serotonin receptors. (*A*) Radioligand binding (RLB) profiles of (+)-JRT and (–)-JRT compared to (+)-LSD. Orange and white cells indicate K_i_ values that are less than or greater than 10 μM, respectively. Data for LSD were obtained from literature values (see *SI Appendix* and Dataset S1). (*B*) BRET-based assay of G_q_ activation indicates that (+)-JRT is a potent partial agonist of 5-HT2A receptors. For antagonist mode (open circles), compounds were cotreated with 10 μM serotonin. (*C* and *D*) PsychLight assays indicate that (+)-JRT is a lower potency 5-HT2A receptor partial agonist than LSD. Differences in potency/efficacy between LSD and (+)-JRT are greater for the wild type human (*C*) than the murinized variant (*D*). For antagonist mode (open circles), compounds were cotreated with 10 μM serotonin. (*E*) Comparison of E_max_/EC_50_ values reveals that LSD is a stronger activator of the wild type human 5-HT2A receptor than the S242A^5.46^ mutant. Data for concentration–response curves represent the mean ± SEM of 3 to 4 biological replicates (performed in duplicate). *P < 0.05, **P < 0.01, ***P < 0.001, ****P < 0.0001. 5-HT = serotonin; ns = not significant; ND = not determined for incomplete curves.

Lack of an indole N–H dramatically alters the pharmacological profile of JRT relative to LSD. Competitive radioligand binding studies across a panel of 55 central nervous system targets revealed that both (+)-JRT and (–)-JRT are highly selective for a subset of serotonin receptors (*SI Appendix*, Fig. S3 and Dataset S1). Unlike LSD, neither of these compounds exhibited affinity for dopamine, histamine, or adrenergic receptors (Fig. 3*A*). Additional studies revealed that (+)-JRT acted as an agonist or partial agonist at 5-HT1A and 5-HT7 receptors as well as an antagonist at 5-HT5A and 5-HT7 receptors (*SI Appendix*, Fig. S4). Moreover, (+)-JRT demonstrated high affinity for the entire family of 5-HT2 receptors (K_i_ range = 2 to 184 nM), while (–)-JRT was substantially less potent (K_i_ range = 495 nM – 32 μM) (*SI Appendix*, Fig. S5). Traditional functional assays relevant to G protein activation revealed that (+)-JRT is a potent partial agonist at 5-HT2A (E_max_ = 81%) and 5-HT2B (E_max_ = 48%) receptors but is nearly a full agonist at 5-HT2C receptors (E_max_ = 89%) (*SI Appendix*, Fig. S5).

Given that (+)-JRT is a potent partial agonist of the 5-HT2A receptor, we were interested in determining whether it possessed pharmacological properties more consistent with a hallucinogenic or nonhallucinogenic ligand. Previous work suggested that the hallucinogenic effects of LSD in mice require β-arrestin 2 (37), and G protein–biased agonists of the 5-HT2A receptor have recently been reported to be nonhallucinogenic (8). Ligand kinetics have been shown to drastically impact biased signaling, with mutations to the 5-HT2AR that accelerate LSD dissociation selectively disrupting β-arrestin recruitment without perturbing G_q_ signaling (38). As disruption of the hydrogen bond between LSD and S242^5.46^ is known to accelerate ligand dissociation (30), we reasoned that the lack of an indole N–H bond might alter the kinetics of (+)-JRT at the 5-HT2A receptor to ultimately impact signaling bias.

To assess relative ligand kinetics at 5-HT2A receptors, we first conducted a series of competition binding assays using 5-HT2A receptor membrane preparations obtained from PSYLI2 cells (6) to determine K_i_ values for LSD, (+)-JRT, and 6-fluoro-*N,N*-diethyltryptamine (6-F-DET) (*SI Appendix*, Fig. 6*A*). The K_i_ values for ligands obtained using native 5-HT2ARs or psychLight2 have been shown to be comparable (39), likely due to the fact that their ligand binding domains are identical. Next, we performed association binding assays to determine the k_on_ and k_off_ values of our radioligand, [^3^H]-LSD (*SI Appendix*, Fig. S6*B*). Using these data, we performed kinetics of competition binding assays at, above, and below the K_i_ value of each ligand to determine k_off_ values (*SI Appendix*, Fig. S6 *C* and *D*). The dissociation rates of (+)-JRT and 6-F-DET from the 5-HT2A receptor were approximately 10- and 100-fold faster than LSD, respectively. As expected, (+)-JRT was not able to recruit β-arrestin through 5-HT2A receptor activation with high efficacy (*SI Appendix*, Fig. S5*D*), despite being a potent agonist of G_q_ signaling (*SI Appendix*, Fig. S5*A*).

Currently, it is unclear whether hallucinogenic potential can be adequately predicted in vitro using ligand bias or partial agonism determined via traditional assays of GPCR activation. Therefore, we also utilized conformational biosensors as more proximal readouts of 5-HT2A receptor activation that are not impacted by signal amplification or receptor reserve (Fig. 3 *B*–*E*). Bioluminescence resonance energy transfer (BRET)-based methods for assessing GPCR activation (40) revealed that (+)-JRT is a potent partial agonist of 5-HT2A receptors leading to G_q_ (Fig. 3*B*), but not G_i_ (*SI Appendix*, Fig. S5 *E* and *F*), activation. As has been observed with other nonhallucinogenic analogues of psychedelics (41), the efficacy of (+)-JRT was reduced substantially compared to its hallucinogenic counterpart LSD (Emax of 33% vs 63%). Antagonist mode experiments confirmed partial agonism (Fig. 3*B*).

Next, we performed psychLight experiments using the wild type human receptor (WT) and a murinized version (S242A^5.46^). When assessing activation of the human receptor, LSD was two orders of magnitude more potent than (+)-JRT with higher efficacy (Emax of 44% vs 33%) (Fig. 3*C*). However, this potency and efficacy difference was attenuated when the S242A^5.46^ mutant version of psychLight was employed (Fig. 3*D*). Analysis of bias using ligand activity ratios (log(E_max_/EC_50_) (42) revealed that LSD exhibits a clear bias for activating the WT human 5-HT2A receptor while (+)-JRT and serotonin do not (Fig. 3*E*). Before testing (+)-JRT in assays relevant to therapeutic efficacy, we confirmed that it exhibited excellent absorption, distribution, metabolism, and excretion (ADME) properties and did not inhibit human ether-a-go-go related gene (hERG) channels (*SI Appendix*, Fig. S7)

### (+)-JRT Promotes Neuroplasticity.

**Fig. 4.**
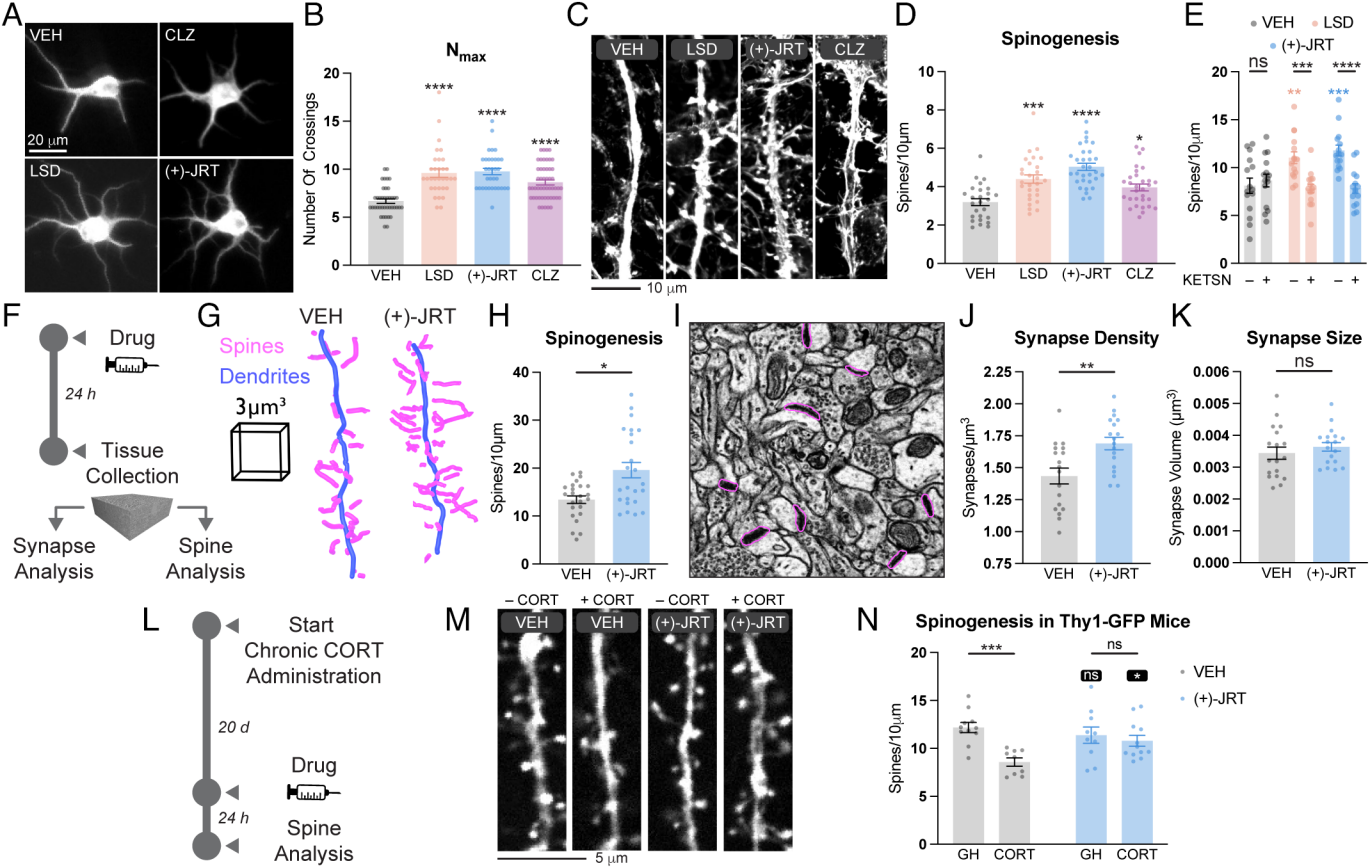
(+)-JRT promotes structural plasticity in vitro and in vivo. (*A*) Representative images of embryonic day 18 (E18) rat cortical neurons (DIV6) treated with compounds (1 μM) demonstrate that (+)-JRT promotes dendritogenesis (white, MAP2). (*B*) Maximum numbers of crossings (N_max_) of the Sholl plots in *SI Appendix*, Fig. S8*A*. (*C*) Representative images of E18 rat embryonic cortical neurons (DIV18) treated with compounds (1 μM) for 24 h demonstrate that (+)-JRT promotes spinogenesis (white, F-actin). (*D*) Quantification of spine density in *F*. (*E*) The 5-HT2 antagonist ketanserin (10 μM) blocks dendritic spine density induced by LSD or (+)-JRT (1 μM). (*F*) Schematic depicting the design of in vivo experiments. Scanning electron microscopy of serial sections demonstrates that a single dose of (+)-JRT (1 mg/kg) increases structural plasticity in the PFC 24 h after administration. (*G*) Representative reconstruction from EM data of dendritic segments (blue) and associated dendritic spines (magenta). (*H*) (+)-JRT promotes spinogenesis in vivo (N = 3 female animals, n = 8 × 27 μm^3^ volumes each per condition). (*I*) Representative S3EM image of mPFC neuropil from animals treated with (+)-JRT 24 h prior to tissue collection. Synapses are highlighted in magenta. (*J*) (+)-JRT promotes synaptogenesis in vivo (3 female animals, 6 fields each per condition). (*K*) (+)-JRT does not impact synapse size in vivo (*L*) Schematic depicting the design of cortical atrophy rescue experiments. (*M*) Representative images of cortical neurons from the PFC of Thy1-EGFP mice exposed to chronic CORT and/or (+)-JRT. (*N*) Confocal microscopy demonstrates that a single dose of (+)-JRT (1 mg/kg) rescues chronic CORT-induced dendritic spine loss in layer 2/3 of the mPFC 24 h after administration (2-way ANOVA). Black asterisks with black bars indicate comparisons within treatment groups (i.e., VEH or JRT. White asterisks indicate comparisons within stress groups (i.e., GH or CORT). VEH = vehicle; LSD = lysergic acid diethylamide; CLZ = clozapine; S3EM = scanning electron microscopy of serial sections; GH = gentle handling; CORT = corticosterone; ns = not significant. *P < 0.05, **P < 0.01, ***P < 0.001, ****P < 0.0001, as compared to VEH control or indicated comparator. See *Materials and Methods* and *SI Appendix*, Table S1 for full details on statistics.

As several nonhallucinogenic analogues of psychedelics with neuroplasticity-promoting properties have recently been identified, (5, 6, 9, 10), we were interested in assessing the effects of (+)-JRT on the growth of cultured rat embryonic cortical neurons. Sholl analysis of DIV6 neurons following treatment with (+)-JRT, LSD, and the atypical antipsychotic clozapine (CLZ) revealed that (+)-JRT exhibited comparable or greater efficacy than LSD or CLZ for promoting dendritic growth (Fig. 4 *A* and *B* and *SI Appendix*, Fig. S8). While all three compounds increased N_max_ values (Fig. 4*B*) and the number of primary dendrites (*SI Appendix*, Fig. S8*B*) compared to the vehicle control, (+)-JRT increased N_max_ values to a greater extent than CLZ (*P* < 0.05). Moreover, both LSD and (+)-JRT promoted dendritic branching, while CLZ did not (*SI Appendix*, Fig. S8*C*). When comparing the areas under the curve of the Sholl plots, only (+)-JRT was statistically significant compared to the vehicle control (*SI Appendix*, Fig. S8*C*). In addition to increasing dendritic arbor complexity, (+)-JRT also promoted dendritic spine growth in more mature cortical cultures (DIV19) as compared to vehicle (*P* < 0.0001) and CLZ (*P* = 0.0005) (Fig. 4 *C* and *D* and *SI Appendix*, Table S1). As with LSD (3), pretreatment with a 5-HT2R antagonist blocked (+)-JRT induced spinogenesis (Fig. 4*E*).

To assess the effects of (+)-JRT on structural plasticity in vivo, we administered the drug to mice (1 mg/kg, IP) and collected tissue from the medial prefrontal cortex (mPFC) 24 h later (Fig. 4*F*). We chose this dose as it approaches the limit of tolerability for LSD, and (+)-JRT demonstrated comparable in vitro potency as LSD. Using scanning electron microscopy of serial sections (S3EM), we collected large high-resolution volumetric datasets from layer 1 of the mPFC and manually segmented dendrites, their dendritic spines, and their synapses. A single administration of (+)-JRT led to a 46% increase in dendritic spine density (Fig. 4 *G* and *H*) and an 18% increase in synapse density (Fig. 4 *I* and *J*) within the mPFC, though synapse size remained unchanged (Fig. 4*K*).

Given that (+)-JRT produced large effects on cortical neuron growth in wild type animals, we were interested to see whether it could rescue structural plasticity deficits induced by chronic stress. First, we administered corticosterone (CORT) daily via intraperitoneal injection to Thy1–enhanced green fluorescent protein (EGFP) mice for 20 d (Fig. 4*L*). These mice have a subset of the neurons in their cortex labeled with EGFP to enable visualization of dendritic spines. Given that layer 2/3 of the mPFC has been implicated in the pathophysiology of SCZ (16), we next administered a single dose of (+)-JRT (1 mg/kg, IP) and measured spine density in this brain region 24 h later. Chronic CORT led to a dramatic decrease in spine density that was rescued by a single dose of (+)-JRT (Fig. 4 *M* and *N*).

### (+)-JRT Does Not Exacerbate the Positive Symptoms of SCZ.

**Fig. 5.**
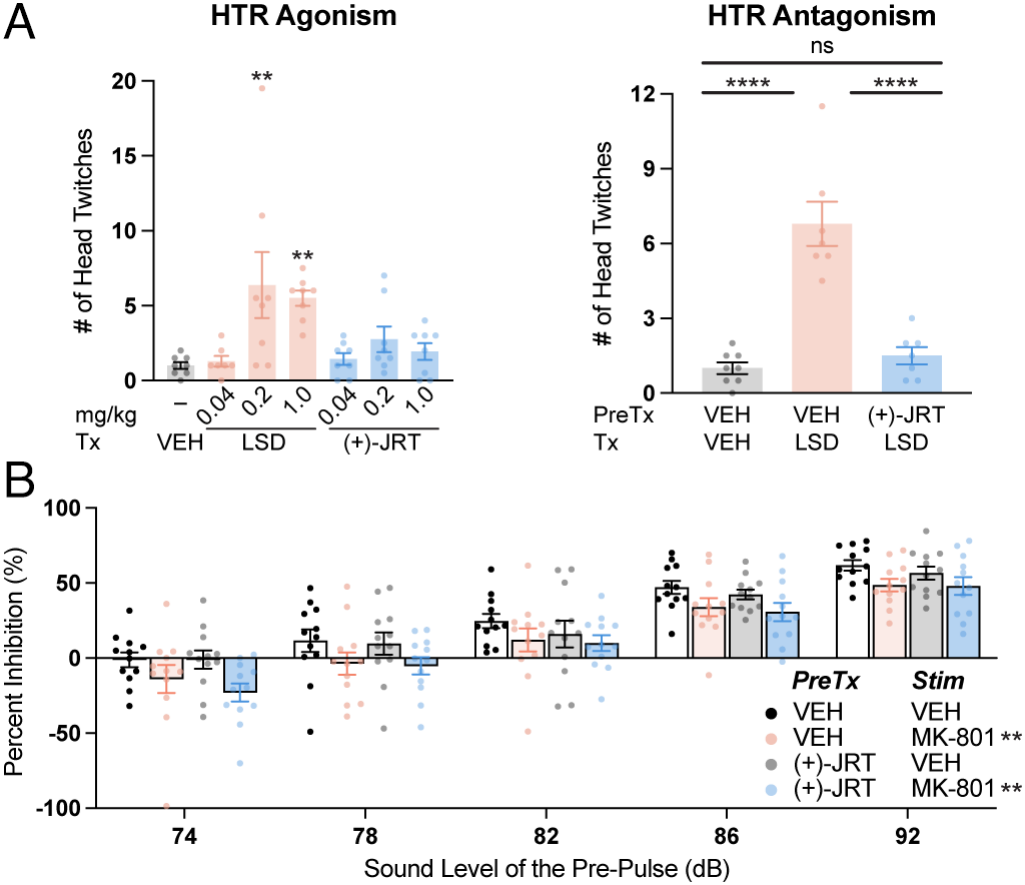
(+)-JRT does not exacerbate hallucinogen-like activity in vivo. (*A*) Mouse HTR assays in male and female animals demonstrate that (+)-JRT has low hallucinogenic potential when the assay is run in agonist mode (head-twitches quantified over 20 min). Furthermore, (+)-JRT (1 mg/kg) demonstrates antipsychotic properties by antagonizing a HTR induced by LSD (0.2 mg/kg). (*B*) Pretreating mice with (+)-JRT (1 mg/kg) does not induce a deficit in prepulse inhibition or exacerbate MK-801-induced deficits. AMPH = (+)-amphetamine; KET = ketamine; PCP = phencyclidine; VEH = vehicle. *P < 0.05, **P < 0.01, ***P < 0.001, ****P < 0.0001, as compared to VEH controls or the comparator(s) indicated by a horizontal bar for *A*. For *B*, a 3-way ANOVA indicated a significant main effect of sound level and MK-801 treatment, but not (+)-JRT treatment, nor any significant interaction effects. ns = not significant. See *Materials and Methods* and *SI Appendix*, Table S1 for full details on statistics.

The in vitro pharmacology profile and robust neuroplasticity-promoting effects of (+)-JRT led us to hypothesize that it might produce beneficial behavioral effects in assays relevant to treating the negative and cognitive symptoms of SCZ without producing behavioral effects characteristic of hallucinogenic compounds. First, we assessed the hallucinogenic potential of (+)-JRT in the mouse head-twitch response (HTR) assay, as potencies in this behavioral test correlate exceptionally well with potencies for producing hallucinations in humans (43). Unlike LSD, (+)-JRT did not induce a robust HTR compared to vehicle control at any dose (Fig. 5*A*). Moreover, pretreatment with (+)-JRT (1 mg/kg, IP) completely blocked the HTR induced by LSD (0.2 mg/kg, IP) (Fig. 5*A*). Similarly, antipsychotics such as CLZ and haloperidol as well as nonhallucinogenic 5-HT2A receptor agonists like lisuride have been shown to block psychedelic-induced HTR (7, 44, 45, 46). To ensure that pretreatment with a high (+)-JRT dose was not confounding our interpretation by shifting the response toward the back side of LSD’s inverted U-shaped dose–response, we performed an additional (+)-JRT blocking study where the dose of LSD was varied. We found the (+)-JRT pretreatment was able to attenuate the HTR induced by LSD at all doses tested (*SI Appendix*, Fig. S9).

While the HTR assay probes serotonergic mechanisms relevant to the positive symptoms of SCZ, dopaminergic mechanisms are also involved (47). We reasoned that the potent 5-HT2C agonist properties of (+)-JRT might block enhanced dopamine signaling in the striatum, and thus, we performed amphetamine-induced hyperlocomotion assays. At a dose of 1 mg/kg, (+)-JRT does not impact locomotion (*SI Appendix*, Fig. S10) or produce any serotonin syndrome-like responses (e.g., backward walking, forepaw treading, hind limb abduction, low body posture, Straub tail, or tremor). However, this dose reduces the hyperlocomotion-inducing effects of (+)-amphetamine (3 mg/kg, IP) in female, but not male, mice (*SI Appendix*, Fig. S11*A*), suggesting that (+)-JRT might attenuate hyperdopaminergic states in female animals despite lacking affinity for D2 receptors. In humans, sex differences in responses to antipsychotics have been observed (48).

Next, we assessed the effects of (+)-JRT in several other assays relevant to the positive symptoms of SCZ. Pretreatment with (+)-JRT did not exacerbate phencyclidine-induced hyperlocomotion (*SI Appendix*, Fig. S11*B*) or MK-801-induced deficits in prepulse inhibition (PPI) (Fig. 5*B*). Importantly, and in sharp contrast to LSD (37), (+)-JRT itself did not induce any deficits in PPI (Fig. 5*B* and *SI Appendix*, Fig. S12), suggesting that it possesses lower hallucinogenic potential.

### (+)-JRT Does Not Promote SCZ-Related Gene Expression.

Given that chronic administration of LSD can result in dysregulated gene expression in the PFC (49) and produce abnormal behaviors in rodents relevant to SCZ (50), we were interested to see whether a single dose (1 mg/kg) of LSD or (+)-JRT led to differential expression of SCZ-related genes in the PFC 24 h after administration. For a SCZ relevant gene set, we used the top 250 loci from a recent transcriptome-wide association study (TWAS) comparing SCZ and control postmortem human brain tissue, ranking the FDR < 0.01 associated loci by absolute log2 FC in SCZ versus control brain samples (51). We performed permutation tests to determine whether the top 250 DEGs following LSD or (+)-JRT administration (*P* < 0.1 ranked by absolute log2 FC) were overrepresented among SCZ TWAS candidate genes. DEGs induced by LSD were indeed more likely to appear in the list of SCZ TWAS DEGs than expected by chance (*P* = 0.018). In contrast, there was no enrichment for (+)-JRT induced DEGs among SCZ-associated loci (*P* = 0.557) (*SI Appendix*, Figs. S13 and S14 and Dataset S2; see *SI Appendix* for additional details).

### (+)-JRT Produces Robust Antidepressant-Like Effects.

**Fig. 6.**
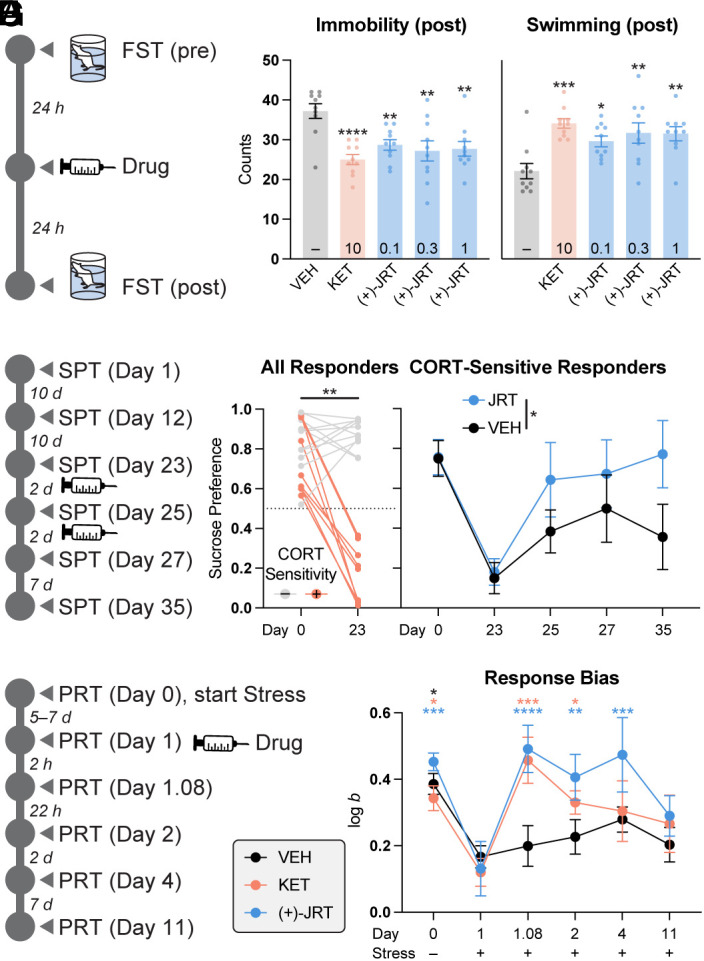
(+)-JRT exhibits antidepressant effects in vivo. (*A*) Schematic depicting a rat FST conducted in males 24 h after compound administration. (*B*) A single dose of (+)-JRT (IP) produces antidepressant-like effects at substantially lower doses than ketamine. Doses (mg/kg) are indicated within the bars representing various treatment groups. (*C*) Schematic depicting CORT-induced anhedonia and pharmacological rescue assessed with the SPT in mice. (*D*) CORT-sensitive animals (+) were defined as sucrose-preferring animals that lost that preference (i.e., score of ≤ 0.5) following 20 d of CORT administration (i.e., Day 23). (*E*) Treatment with (+)-JRT (1 mg/kg, IP) rescued CORT-induced deficits in the SPT (2-way ANOVA). (*F*) Schematic depicting chronic cold-water stress-induced anhedonia and pharmacological rescue assessed with the PRT in rats. (*G*) Chronic cold-water stress leads to deficits in response bias (log *b*) that is rescued by both KET (10 mg/kg, IP) and (+)-JRT (1 mg/kg). KET = ketamine; VEH = vehicle; CORT = corticosterone; SPT = sucrose preference test; PRT = probabilistic reward task. *P < 0.05, **P < 0.01, ***P < 0.001, ****P < 0.0001, as compared to VEH controls (*A*), the comparator indicated by horizontal/vertical bars (*D* and *E*), or Day 1 log *b* values (*G*) for the same treatment (indicated by the color of the asterisks). See *Materials and Methods* and *SI Appendix*, Table S1 for full details on statistics.

To determine whether (+)-JRT might impact the negative symptoms of SCZ, we subjected rats to a forced swim test (Fig. 6*A*)—an assay that is highly predictive of antidepressant potential (52). Pharmacokinetic studies indicated that the highest dose tested (1 mg/kg) produced brain concentrations ~20-fold higher than the K_i_ of (+)-JRT at the 5-HT2AR (*SI Appendix*, Fig. S15). As a positive control, we used the lowest dose of ketamine that reliably produces an antidepressant response in this assay (10 mg/kg, IP). We found that (+)-JRT was exceptionally potent, decreasing immobility and increasing swimming behavior at all doses tested 24 h after administration (Fig. 6*B*). Though we did not determine the lowest effective dose, it seems that (+)-JRT is at least 100-fold more potent than ketamine in this assay. Moreover, (+)-JRT produces sustained antidepressant-like effects, as it is nearly absent from both rat brain and plasma within 2 h following a 1 mg/kg IP dose (*SI Appendix*, Fig. S15).

Next, we employed several assays relevant to anhedonia. Given that chronic CORT administration resulted in cortical atrophy (Fig. 4 *L*–*N*), we used this stressor to induce a deficit in the sucrose preference test (SPT) (Fig. 6*C*). Mice were first classified as CORT-sensitive if their baseline preference for sucrose was eliminated following chronic CORT administration (Fig. 6*D*). Next, (+)-JRT (1 mg/kg) was administered via two intraperitoneal injections spaced 48 h apart with SPT assessments conducted immediately prior to drug administration as well as 2 and 9 d after the last dose. There was an overall main effect of treatment (F (1, 7) = 6.664; *P* = 0.0364) but no interaction effect of treatment and time (F (4, 28) = 0.6584; *P* = 0.6260), indicating that (+)-JRT treatment rescued CORT-induced anhedonia (Fig. 6*E*).

Given the challenges associated with translating mouse behavior to humans in neuropsychiatry, we decided to utilize a PRT (53) as a measure of anhedonia (Fig. 6*F*). This assay is a recommended task to probe positive valence systems in the latest revision of the RDoC matrix (54) and has been reverse translated using touchscreen technology (55) for rodents (56, 57) and nonhuman primates (58). The PRT uses visual discrimination methodology to quantify reward responsivity. Subjects make visual discriminations and probabilistic contingencies are arranged such that correct responses to one alternative are rewarded more often (rich) than correct responses to the other (lean). Healthy human participants consistently develop a highly adaptive response bias in favor of the rich alternative, and deficits in response biases are observed in major depressive disorder (53) and SCZ (59). Moreover, these deficits can be induced by early-life adversity or chronic stress (60, 61), correlate with current anhedonia, and predict future anhedonia (53, 62, 63).

A repeated measures analysis revealed that chronic cold-water stress reduced response bias (log *b*), and both ketamine and (+)-JRT were able to rescue this deficit. Notably, the effect of (+)-JRT persisted for at least 3 d following a single dose, even in the presence of continued daily cold-water stress (Fig. 6*G*). Discriminability (log *d*) and reactions’ time metrics remained stable over the length of the experiment (*SI Appendix*, Fig. S16).

### (+)-JRT Promotes Cognitive Flexibility.

**Fig. 7.**
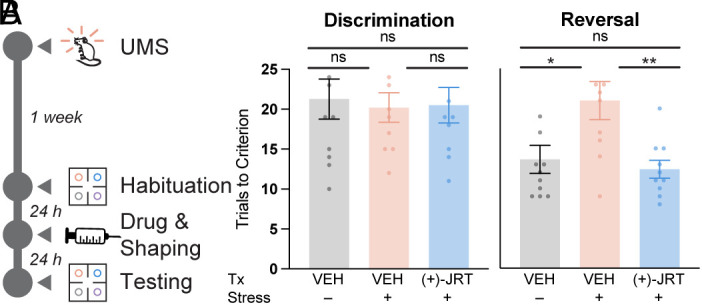
(+)-JRT exhibits procognitive effects in vivo. (*A*) Schematic depicting UMS-induced impairments in cognitive flexibility and pharmacological rescue assessed with a 4-odor discrimination and reversal assay. (*B*) A single dose of (+)-JRT (1 mg/mL, IP) does not impact stimulus discrimination but rescues cognitive deficits induced by UMS. UMS = unpredictable mild stress; VEH = vehicle; ns = not significant. *P < 0.05, **P < 0.01, as compared to the comparator indicated by a horizontal bar. ns = not significant. See *Materials and Methods* and *SI Appendix*, Table S1 for full details on statistics.

Finally, to assess cognitive flexibility, we performed a reversal learning experiment in mice following 1 wk of unpredictable mild stress (UMS) (Fig. 7*A*). Before the shaping session on the second day of training, the animals were given a single dose of (+)-JRT (1 mg/kg, IP). The following day, there was no difference observed in the discrimination test between stressed or unstressed animals given the vehicle control and stressed animals treated with (+)-JRT (Fig. 7*B*). However, UMS led to a deficit in reversal learning that was completely rescued by treatment with (+)-JRT (Fig. 7*B*). There was no statistical difference in the total number of trials completed, though the number of omission trials approached significance (*P* = 0.07) when comparing stressed animals that received vehicle or (+)-JRT (*SI Appendix*, Fig. S17). As patients with SCZ and bipolar disorder are known to have deficits in attentional set-shifting and reversal learning (64, 65), the procognitive effects of (+)-JRT could have important therapeutic implications.

## Discussion

Effective treatments for complex neuropsychiatric diseases like depression, substance use disorders, and SCZ are likely to involve multiple targets rather than a single site of action (66). However, the polypharmacology of such agents must be carefully tuned to maximize benefit while minimizing unwanted side effects. The unique polypharmacology of (+)-JRT might endow it with specific advantages compared to compounds currently in use. For example, antipsychotics that block the positive symptoms of SCZ through D2 antagonism often lead to extrapyramidal side effects. In contrast, (+)-JRT can block amphetamine-induced hyperlocomotion in female mice without exhibiting any appreciable affinity for D2 receptors. This effect is likely due to its potent 5-HT2C agonism given that 5-HT2C agonists have been shown to reduce dopamine efflux in the nucleus accumbens (67) and produce antipsychotic effects in rodent assays relevant to the positive symptoms of SCZ (68). However, (+)-JRT is also a 5-HT2B receptor partial agonist, and chronic stimulation of this receptor has the potential to induce cardiac valvulopathy (69).

In contrast to the typical antipsychotic haloperidol, the atypical antipsychotic CLZ has proven much more efficacious for treating SCZ, perhaps due to the diversity of receptors that it targets (70). Despite its greater efficacy, clozapine is often reserved for patients with treatment-resistant SCZ as its promiscuity is associated with a number of undesirable side effects including weight gain, metabolic issues, and sedation (70). Unlike CLZ, (+)-JRT lacks affinity for dopamine, histamine, muscarinic, and adrenergic receptors, perhaps explaining why it does not appear to produce sedative effects. Moreover, CLZ exhibits 5-HT2C antagonism, which is believed to contribute to metabolic dysfunction and weight gain—side effects that are not predicted to plague (+)-JRT, as it is a potent agonist of 5-HT2C receptors.

Antagonism of 5-HT2A receptors is hypothesized to play a key role in the mechanism by which CLZ and other antipsychotics block the positive symptoms of SCZ. However, agonism of 5-HT2A receptors has the potential to ameliorate the negative and cognitive symptoms of the disease—aspects that have historically proven much more difficult to address, but that correlate better with treatment response (23). In fact, 5-HT2A receptor agonists have been shown to produce antidepressant effects in both rodents (71–74) and humans (75, 76, 77), and recent evidence suggests that LSD may have procognitive effects (78). Moreover, psychedelics are among the most effective psychoplastogens known, producing robust effects on cortical neuron growth in the PFC after a single administration (3, 4, 39, 79). The ability of (+)-JRT to promote cortical neuron growth without hallucinogenic effects is likely due to its partial 5-HT2AR agonist properties, though this should be confirmed in future studies utilizing 5-HT2AR knockout mice. Emerging evidence suggests that a threshold level of Gq activation is required for 5-HT2AR ligands to induce hallucinations (41), and partial agonism appears to be sufficient to maintain effects on structural neuroplasticity (5).

Their ability to promote spine growth in the PFC makes psychedelics an attractive treatment option for diseases characterized by cortical atrophy, but their hallucinogenic properties preclude their use in patients with a psychotic illness or a family history of such disorders. In fact, several recent psilocybin clinical trials for depression excluded ~95% of volunteers due to contraindications (12). Unlike LSD (37), (+)-JRT does not produce any of the hallmark preclinical behavioral characteristics of hallucinogens such as a HTR, hyperlocomotion, or deficits in PPI, nor does it lead to the differential expression of genes associated with SCZ.

While rodent behavioral and gene expression studies demonstrated a clear difference in the hallucinogenic potential of (+)-JRT compared to LSD, this difference might be even greater in humans given that LSD is biased toward activating the human 5-HT2A receptor. In the human 5-HT2A receptor, the indole N–H of LSD has been shown to hydrogen bond to S242^5.46^, but (+)-JRT lacks the ability to engage this residue in a similar manner. Interestingly, mutation of S242^5.46^ to an alanine attenuates the potency and efficacy differences between LSD and (+)-JRT.

Despite its lower hallucinogenic potential, (+)-JRT has demonstrated profound therapeutic effects. In head-to-head in vitro comparisons with both LSD and CLZ, (+)-JRT demonstrated superior effects on cortical neuron growth. Moreover, (+)-JRT produced a remarkable 46% increase in dendritic spine density in vivo as measured by electron microscopy in layer 1. Immunohistochemistry experiments also demonstrated that (+)-JRT completely rescued cortical atrophy in layer 2/3 of Thy1-EGFP neurons following 20 d of chronic CORT administration. Interestingly, (+)-JRT did not promote the growth of Thy1-EGFP neurons beyond baseline levels in layer 2/3.

These changes in structural plasticity were accompanied by robust antidepressant-like properties and procognitive effects in a number of assays including traditional tests measuring active coping strategies in response to an unavoidable stressor (FST) and the rescue of an anhedonia phenotype following chronic stress (SPT). Additionally, (+)-JRT demonstrated robust antidepressant-like activity in the PRT and promoted cognitive flexibility in a reversal learning task—assays that are highly translatable from preclinical species to humans. Unlike (+)-JRT, selective 5-HT2C receptor agonists are unable to rectify reversal learning deficits in rodents (68), suggesting that the unique pharmacological profile of (+)-JRT at 5-HT2A receptors might confer some additional therapeutic benefit.

Given that 5-HT2A receptors are believed to be responsible for the hallucinogenic and neuroplasticity-promoting properties of psychedelics (3, 39, 74, 79, 80), identifying 5-HT2A receptor partial agonists that can promote cortical neuron growth without hallucinogenic effects will be essential for treating the negative and cognitive symptoms of SCZ. Such ligands have the potential to produce effects characteristic of both 5-HT2A antagonists and agonists. For example, (+)-JRT acts as a partial antagonist in psychLight and BRET-based assays of 5-HT2A receptor activation and blocks psychedelic-induced HTR, yet it promotes cortical neuron growth and produces antidepressant-like effects. Recent studies have identified several novel nonhallucinogenic ligands of 5-HT2A receptors that act as partial agonists in traditional assays of GPCR activation. Some of these compounds have been shown to promote cortical neuron growth (5, 9), while it is unclear whether the others possess similar neuroplasticity-promoting properties (7, 8).

Here, we demonstrate that (+)-JRT possesses unique pharmacological properties as a selective serotonergic agent that can potentially address negative and cognitive symptoms associated with SCZ without exacerbating positive symptoms or producing the side effects characteristic of current treatments. Remarkably, this highly beneficial pharmacological profile was achieved through chemical transposition of only two atoms within the ergoline core structure of LSD. Taken together, our work highlights the potential for modifying the chemical structures of psychedelics to produce analogues with improved efficacy and safety profiles and further underscores the potential usefulness of nonhallucinogenic psychoplastogens for treating illnesses unlikely to be addressed by psychedelics such as SCZ, bipolar disorder, and psychosis in neurodegenerative diseases.

## Materials and Methods

### Chemical Synthesis.

All reagents were obtained from commercial sources and reactions were performed using oven-dried glassware (120 °C) under an inert N_2_ atmosphere unless otherwise noted. Air- and moisture-sensitive liquids and solutions were transferred via syringe or stainless-steel cannula. Organic solutions were concentrated under reduced pressure (∼5 Torr) by rotary evaporation. Solvents were purified by passage under 12 psi N_2_ through activated alumina columns. Chromatography was performed using Fisher Chemical™ Silica Gel Sorbent (230 to 400 Mesh, Grade 60). Compounds purified by chromatography were typically applied to the adsorbent bed using the indicated solvent conditions with a minimum amount of added dichloromethane as needed for solubility. Thin layer chromatography was performed on Merck silica gel 60 F254 plates (250 μm). Visualization of the developed chromatogram was accomplished by fluorescence quenching or by staining with aqueous potassium permanganate or Ehrlich’s reagent. NMR spectra were acquired on a Bruker 400 operating at 400 and 100 MHz for ^1^H and ^13^C, respectively, and are referenced internally according to residual solvent signals. Data for ^1^H NMR are recorded as follows: chemical shift (δ, ppm), multiplicity (s, singlet; d, doublet; t, triplet; q, quartet; quint, quintet; m, multiplet), coupling constant (Hz), and integration. Data for ^13^C NMR are reported in terms of chemical shift (δ, ppm). Infrared spectra were recorded using a Thermo Nicolet iS10 Fourier transform infrared spectrometer with a Smart iTX Accessory (diamond attenuated total reflection) and are reported in the frequency of absorption (ν, cm^−1^). Liquid chromatography-mass spectrometry (LC–MS) was performed using a Waters LC–MS with an ACQUITY Arc QDa detector. Specific rotation measurements were performed on an AUTOPOL IV Automatic Digital Polarimeter. Detailed methods for each reaction and characterization data are provided in *SI Appendix*.

### Pharmacology.

Treatments were randomized, and data were analyzed by experimenters blinded to treatment conditions. Statistical analyses were performed using GraphPad Prism (version 10.0.3) unless noted otherwise. All comparisons were planned prior to performing each experiment. No data were excluded. Data are represented as mean ± SEM, unless noted otherwise, with asterisks indicating *P < 0.05, **P < 0.01, ***P < 0.001, and ****P < 0.0001. Details of all statistical tests are shown in *SI Appendix*, Table S1. Many of the drugs used in these studies were purchased from commercial sources including (+)-amphetamine sulfate (Sigma Aldrich, 1180004), ketamine hydrochloride (Spectrum, K1068), and ketanserin (APExBIO, B2248). LSD hemitartrate was generously provided by the NIH Drug Supply Program. Both (+)-JRT and (–)-JRT were synthesized in-house and judged to be analytically pure based on NMR and LC–MS data. For cell culture experiments, VEH = 0.1% (agonist studies) or 0.2% (antagonist studies) molecular biology grade dimethyl sulfoxide (Sigma-Aldrich). For in vivo experiments, compounds were administered i.p. at 5 mL/kg using 0.9% saline as the vehicle, unless noted otherwise. VEH = USP grade saline (0.9%). Stock solutions for behavioral assays were prepared fresh before use. Fumarate salts of (+)-JRT and (–)-JRT were used for all biological studies unless noted otherwise. All experimental procedures involving animals were approved by the Institutional Animal Care and Use Committee at the University of California, Davis, the Salk Institute, Weill Cornell Medicine, McLean Hospital (Harvard Medical School), or the Contract Research Organization where the study was performed. All procedures involving animals adhered to principles described in the NIH Guide for the Care and Use of Laboratory Animals. Animals were either obtained from Jackson Laboratory (Sacramento, CA) or bred in-house unless noted otherwise. Power analyses were conducted to ensure appropriate sample sizes for all experiments involving animals. Animals were housed 2 to 5 animals of the same sex per cage on a 12 h light/dark cycle and were given ad libitum access to food and water unless noted otherwise. The University of California, Davis, the Salk Institute, and Weill Cornell Medicine are accredited by the Association for Assessment and Accreditation of Laboratory Animal Care International. Whenever possible, both sexes were utilized. However, a single sex was utilized in some cases when 1) a contract research organization had only validated the assay with a single sex, or 2) previous data indicated that both sexes respond similarly in the assay. Detailed materials and methods for each pharmacology experiment are provided in *SI Appendix*.

## Supplementary Material

Appendix 01 (PDF)

Dataset S01 (XLSX)

Dataset S02 (CSV)

## Data Availability

Data are available at the following link https://doi.org/10.6084/m9.figshare.21579507 (81). All materials are available upon request or are commercially available. The X-ray data for compound **16** have been deposited at the Cambridge Crystallographic Data Centre (CCDC) database and are available free of charge under CCDC number 2220431 (82). All other data are available in the manuscript or supporting information.
