# Supplementary material for: Molecular design of a therapeutic LSD analogue with reduced hallucinogenic potential
Source: Proc Natl Acad Sci U S A. 2025 Apr 14;122(16):e2416106122. doi: 10.1073/pnas.2416106122 (PMC12037037; doi:10.1073/pnas.2416106122)
Supplement: Supplementary file 1 — Appendix 01 (PDF) [file pnas.2416106122.sapp.pdf]

PNAS

Supporting Information for

Molecular Design of a Therapeutic LSD Analogue with Reduced Hallucinogenic Potential

Jeremy R. Tuck, Lee E. Dunlap, Yara A. Khatib, Cassandra J. Hatzipantelis, Sammy Weiser Novak, Rachel M. Rahn, Alexis R. Davis, Adam Mosswood, Anna M. M. Vernier, Ethan M. Fenton, Isak K. Aarrestad, Robert J. Tombari, Samuel J. Carter, Zachary Deane, Yuning Wang, Arlo Sheridan, Monica A. Gonzalez, Arabo A. Avanes, Noel A. Powell, Milan Chytil, Sharon Engel, James C. Fettinger, Amaya R. Jenkins, William A. Carlezon Jr., Alex S. Nord, Brian D. Kangas, Kurt Rasmussen, Conor Liston, Uri Manor, David E. Olson*

Corresponding Author: David E. Olson
Email: deolson@ucdavis.edu

This PDF file includes:

Figures S1 to S17
Table S1
Legend for Dataset S1
Legend for Dataset S2
Materials and Methods
General Information for Chemical Synthesis
Detailed Synthetic Procedures and Experimental Data for All Compounds
X-ray Crystallography
^{1}H and ^{13}C NMR Spectra
Supplemental References

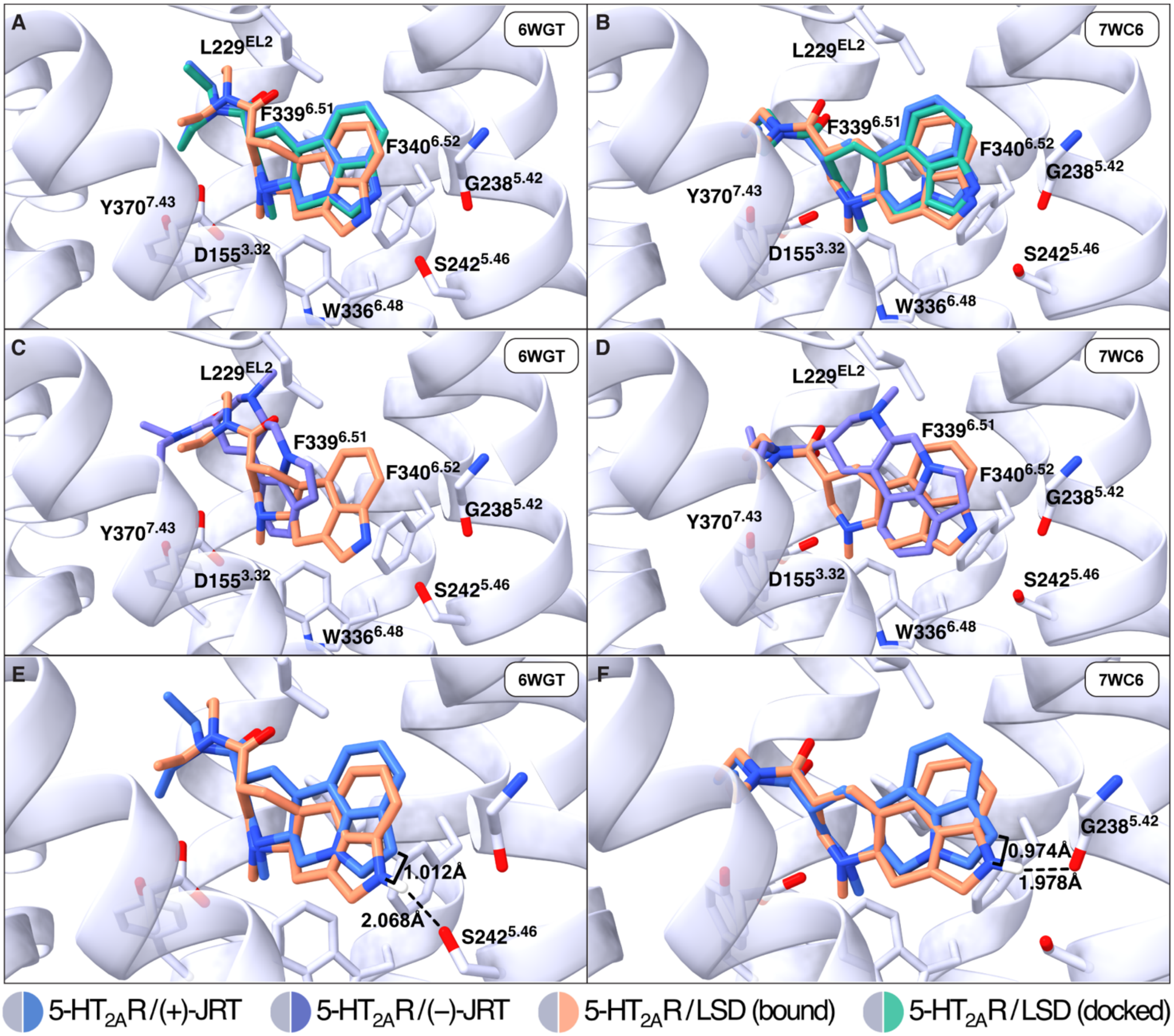

Figure S1. Docking of JRT enantiomers in the 5-HT2AR binding pocket. (**A–B**) Molecular docking of JRT (assigned (+)-JRT based on comparison of the absolute stereochemistry relative to (+)-LSD) into the crystal structure of the 5-HT2AR bound to LSD (PDB: 6WGT (Figure S1A) and 7WC6(Figure S1B)). The orange and green chemical structures of LSD indicate the position of LSD observed in the crystal structure and predicted based on docking, respectively. The docked (+)-LSD structures are in close agreement with the crystal structure data (RMSD = 1.024 for 6WGT and RMSD = 0.460 for 7WC6). The blue chemical structure indicates the docked position of (+)-JRT, which shares many similarities to the position of LSD in the respective binding pockets. (**C**–**D**) Molecular docking of the enantiomer of JRT (assigned (–)-JRT based on comparison of the absolute stereochemistry relative to (+)-LSD) demonstrates limited homology to the binding poses of LSD observed in the respective crystal structures (RMSD = 4.576 for 6WGT (Figure S1C) and RMSD = 3.171 for 7WC6 (Figure S1D)). (**E**–**F**) The primary difference between the position of LSD and the position of docked (+)-JRT is that the latter compound is further removed from $S242^{5.46}$ (Figure S1E) or $G238^{5.42}$ (Figure S1F). Hydrogen bonding distances between bound LSD and either $S242^{5.46}$ or $G238^{5.42}$ are shown, alongside the associated shift between homologous positions of the indoles for docked JRT. The binding modes with the lowest RMSD scores relative to native bound (+)-LSD are shown. Transmembrane helix 4 was removed for clarity. LSD = lysergic acid diethylamide.

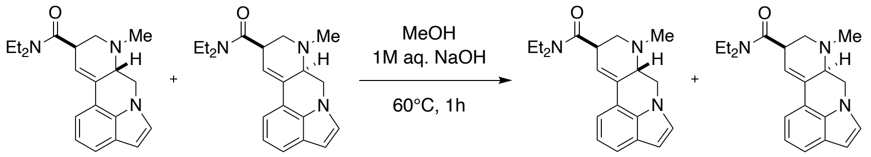

14 [1:2] **15** → **14** [5:1] **15**

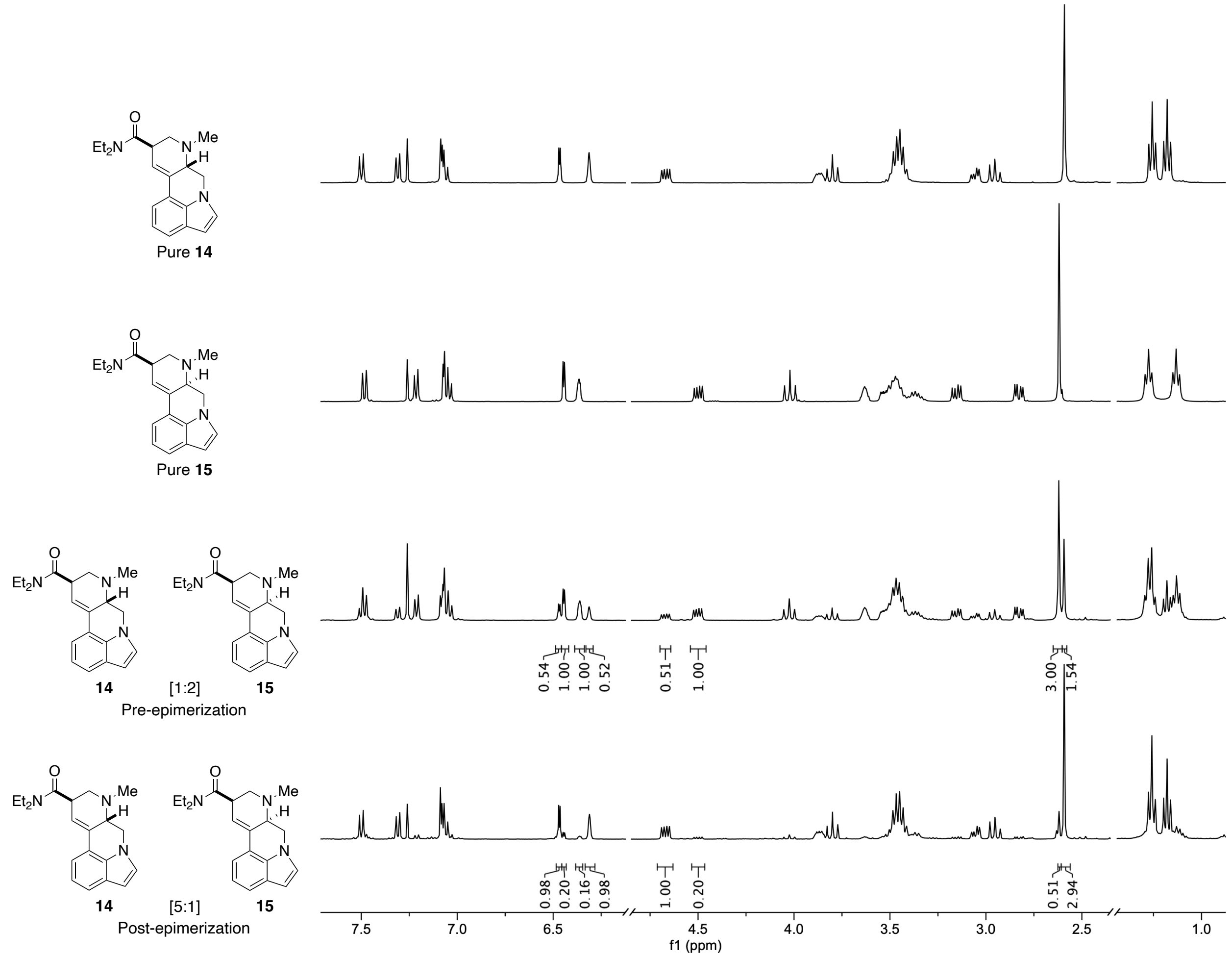

Figure S2. Thermodynamic equilibrium studies. To a solution of diastereomers **14** and **15** (1:2 ratio, measured via ^{1}H NMR analysis) (0.014 g, 0.043 mmol, 1.0 equiv) in MeOH (1 mL) was added 2M aqueous NaOH (0.5 mL). The solution was heated at 60°C for 1 h and then concentrated under reduced pressure. The mixture was partitioned in H_2O (20 mL) and DCM (10 mL) and the layers were separated. The aqueous layer was further extracted with DCM (2 x 10 mL). The organic extracts were combined, washed with brine (20 mL), dried over Na_2SO_4, and concentrated under reduced pressure. Diastereomers **14** and **15** were obtained as a 5:1 mixture (measured via ^{1}H NMR analysis in $CDCl_3$), with **14** as the major diastereomer and **15** as the minor diastereomer, indicating that **14** is the thermodynamically favored product.

Assay Name	(–)-JRT	(+)-JRT
5-HT1A (h) (agonist radioligand)	**59**	**91**
5-HT1B (h) (antagonist radioligand)	0	28
5-HT2A (h) (antagonist radioligand)	**88**	**99**
5-HT2B (h) (agonist radioligand)	**97**	**101**
5-HT3 (h) (antagonist radioligand)	-4	-7
5-HT5a (h) (agonist radioligand)	**70**	**96**
5-HT6 (h) (agonist radioligand)	39	**83**
5-HT7 (h) (agonist radioligand)	25	**84**
A1 (h) (antagonist radioligand)	10	18
A2A (h) (agonist radioligand)	-6	-15
A3 (h) (agonist radioligand)	14	13
alpha 1 (non-selective) (antagonist radioligand)	5	11
alpha 2 (non-selective) (antagonist radioligand)	0	-6
AT1 (h) (antagonist radioligand)	0	-4
B2 (h) (agonist radioligand)	-11	-6
beta 1 (h) (agonist radioligand)	-3	0
beta 2 (h) (antagonist radioligand)	1	-6
BZD (central) (agonist radioligand)	-6	-11
Ca2+ channel (L, verapamil site) (phenylalkylamine) (antagonist radioligand)	2	13
CB1 (h) (agonist radioligand)	3	-1
CCK1 (CCKA) (h) (agonist radioligand)	21	6
CCR1 (h) (agonist radioligand)	-5	-3
Cl- channel (GABA-gated) (antagonist radioligand)	6	6
CXCR2 (IL-8B) (h) (agonist radioligand)	-1	-7
D1 (h) (antagonist radioligand)	-1	27
D2S (h) (antagonist radioligand)	-3	4
delta (DOP) (h) (agonist radioligand)	4	-3
EP4 (h) (agonist radioligand)	1	-1
ETA (h) (agonist radioligand)	12	12
GABA (non-selective) (agonist radioligand)	3	-4
GAL2 (h) (agonist radioligand)	1	5
H1 (h) (antagonist radioligand)	-4	-11
H2 (h) (antagonist radioligand)	-4	-4
kappa (h) (KOP) (agonist radioligand)	10	7
KV channel (antagonist radioligand)	6	3
M1 (h) (antagonist radioligand)	0	-4
M2 (h) (antagonist radioligand)	-3	-1
M3 (h) (antagonist radioligand)	-7	4
MC4 (h) (agonist radioligand)	5	5
MT1 (ML1A) (h) (agonist radioligand)	25	21
mu (MOP) (h) (agonist radioligand)	-2	-8
Na+ channel (site 2) (antagonist radioligand)	4	-17
NK2 (h) (agonist radioligand)	**67**	39
NK3 (h) (antagonist radioligand)	-27	-20
NOP (ORL1) (h) (agonist radioligand)	-18	-24
NTS1 (NT1) (h) (agonist radioligand)	9	-7
SKCa channel (antagonist radioligand)	-1	-5
sst (non-selective) (agonist radioligand)	-9	-15
Transporter: 5-HT (h) (antagonist radioligand)	-14	-9
Transporter: DA (h) (antagonist radioligand)	10	4
Transporter: NET (h) (antagonist radioligand)	-6	-4
V1a (h) (agonist radioligand)	7	10
VPAC1 (VIP1) (h) (agonist radioligand)	-4	-2
Y1 (h) (agonist radioligand)	8	5
Y2 (h) (agonist radioligand)	1	-1

Figure S3. Selectivity profiles for (+)-JRT and (–)-JRT across 55 central nervous system targets. The effects of (+)-JRT (10 μM) and (–)-JRT (10 μM) on a wide range of targets were assessed by Eurofins Discovery. Data represent % inhibition of the binding of a radioactively labeled ligand specific for each target. Assays were conducted in duplicate and the results were averaged. Targets with ≥50% inhibition are highlighted in blue.

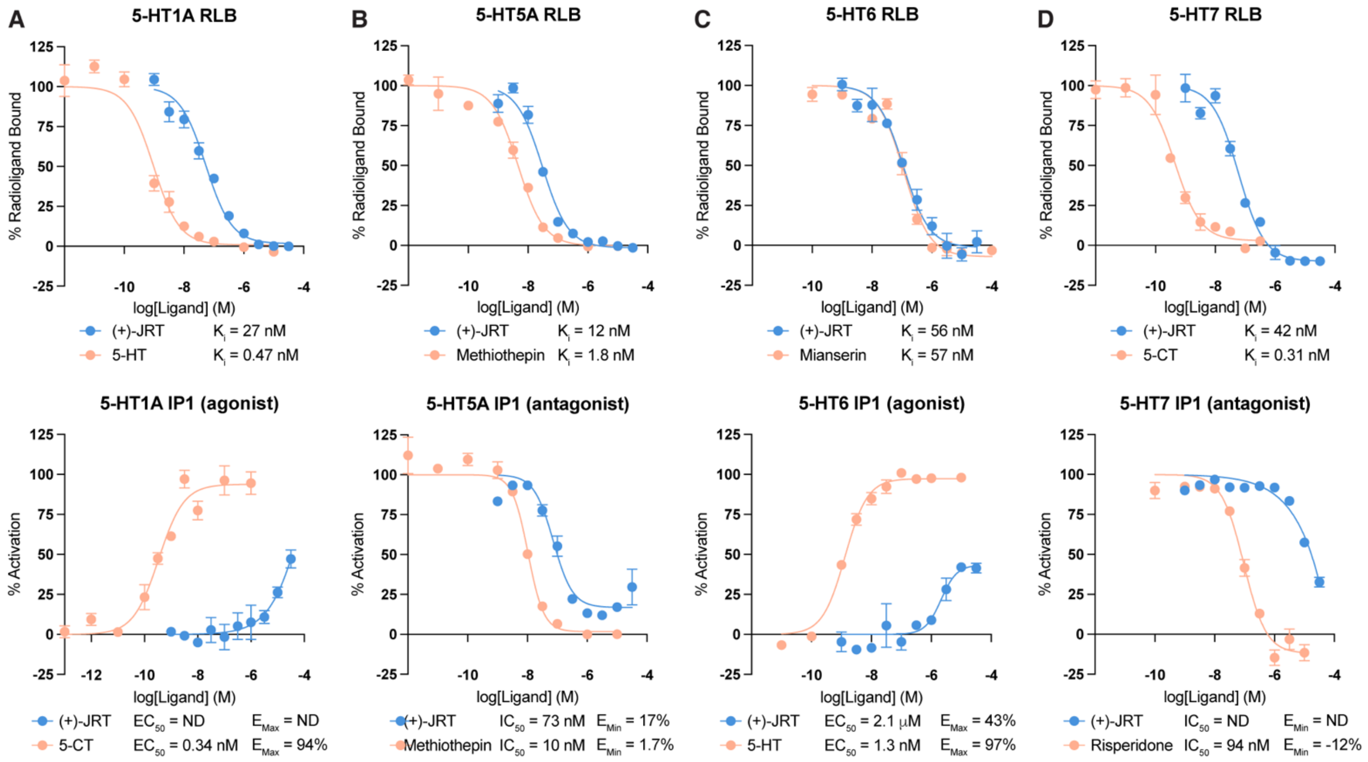

Figure S4. (+)-JRT is a potent ligand of several serotonin receptors. Radioligand binding (RLB) (top) and IP1 functional assays (bottom) for 5-HT1A (**A**), 5-HT5a (**B**), 5-HT6 (**C**), and 5-HT7 (**D**) receptors. Agonist or antagonist mode is indicated in the title of each graph. Data represent the mean ± SEM of 2 technical replicates. 5-HT = serotonin; 5-CT = 5-carboxamidotryptamine; ND = not determined for incomplete curves.

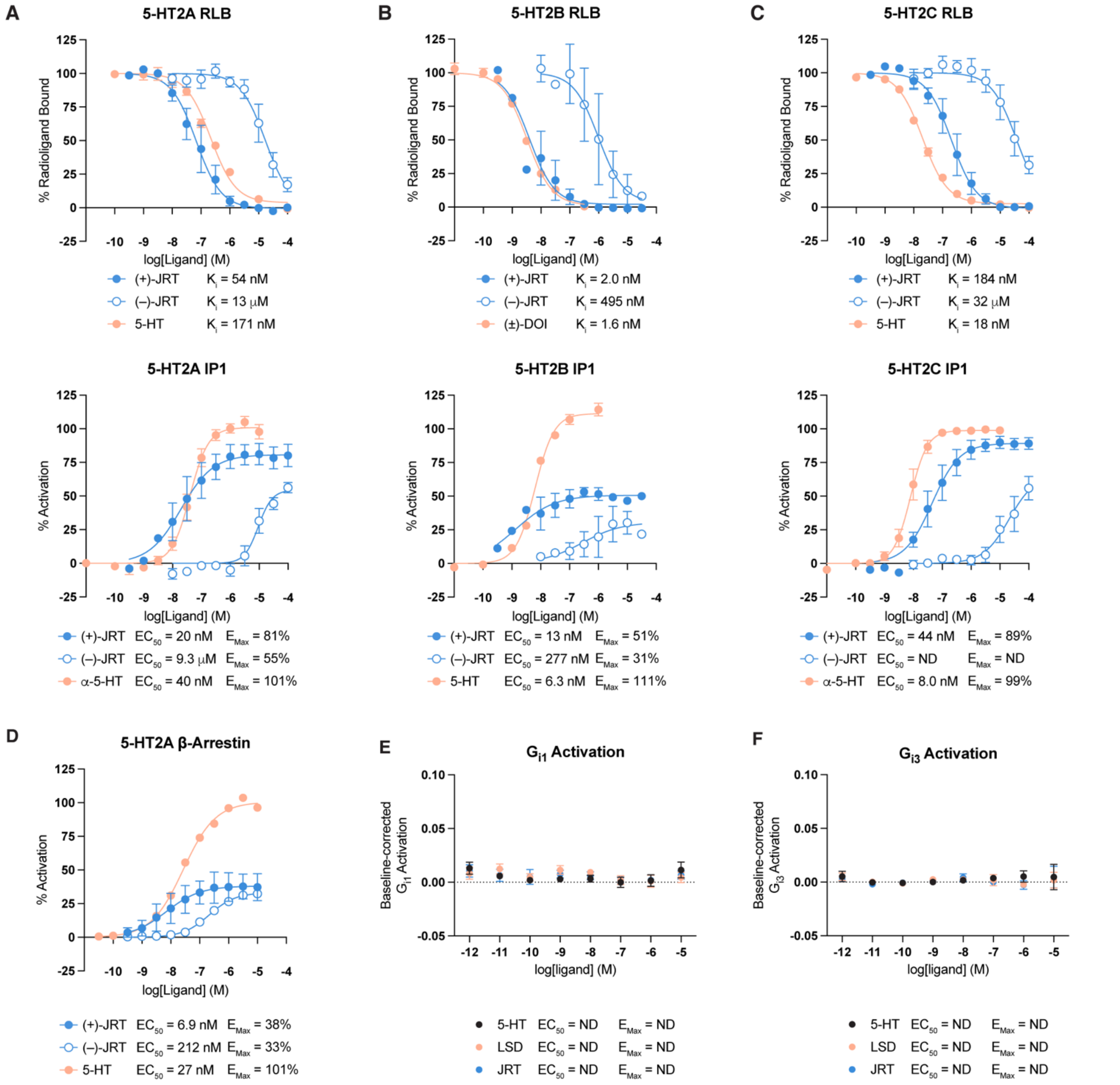

Figure S5. (+)-JRT is a potent agonist of 5-HT2 receptors. Radioligand binding (RLB) (top) and IP1 functional assays (bottom) for 5-HT2A (**A**), 5-HT2B (**B**), and 5-HT2C (**C**) receptors. (**D**) The PathHunter® β-arrestin assay was used to demonstrate that (+)-JRT is a potent partial agonist of the β-arrestin pathway in U2OS cells expressing 5-HT2ARs. (**E**–**F**) BRET-based assays of G_{i1} (**E**) and G_{i3} (**F**) activation indicate that (+)-JRT does not activate G_i following stimulation of 5-HT2A receptors. Data represent the mean ± SEM of 2–6 biological replicates (performed in duplicate), with the sole exception that the β-arrestin data for (–)-JRT represents a single biological replicate averaged from 2 technical replicates. 5-HT = serotonin; α-5-HT = α-methylserotonin; DOI = 2,5-dimethoxy-4-iodoamphetamine; ND = not determined for incomplete curves.

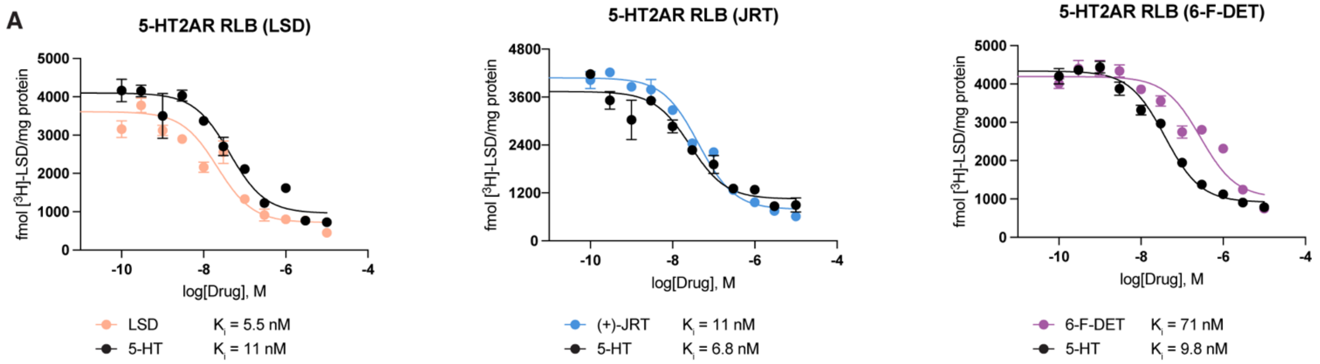

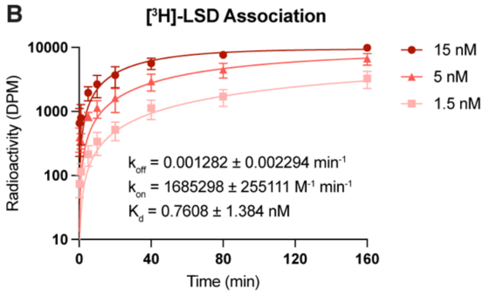

C

Compound	K_i (nM)	Concentrations Tested (nM)	k_{off} ± SEM (min^{-1})	k_{on} ± SEM (M^{-1} min^{-1})
LSD	5.5	1.7, 5.5, 7.2	0.001459 ± 0.002206	7949911 ± 1545685
(+)-JRT	11	3.2, 10.9, 14.2	0.01153 ± 0.004518	6974561 ± 1912729
6-F-DET	71	53.4, 71.2, 92.6	0.08170 ± 0.02385	2953653 ± 1402848

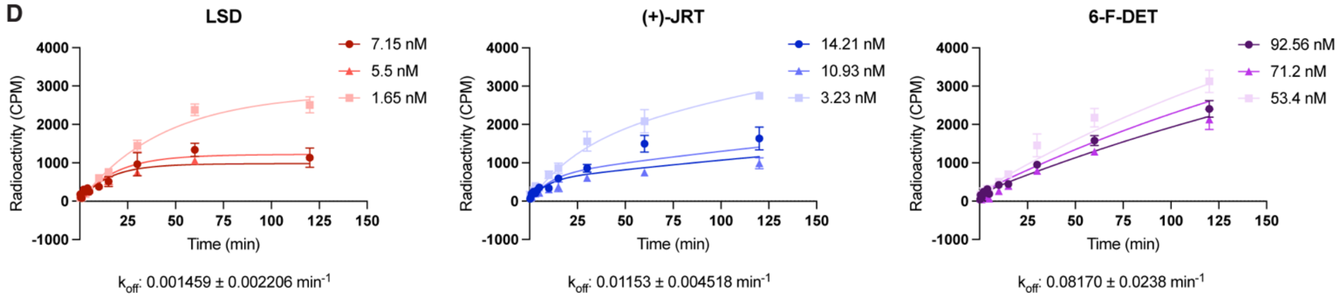

Figure S6. Ligand binding kinetics at the 5-HT2AR. (**A**) Radioligand competition binding assays using membrane preparations from PSYLI2 cells were performed to determine the K_i values (nM) of LSD, (+)-JRT, and 6-F-DET. Serotonin (5-HT) was used as a plate control. Data represent the mean ± SEM of 2–3 biological replicates. (**B**) Association binding assays using membrane preparations from PSYLI2 cells enabled the determination of dissociation (k_{off}) and association (k_{on}) rate constants for [^{3}H]-LSD. Data represent the mean ± SEM of 2–4 biological replicates. (**C**–**D**) Kinetics of competition binding assays enabled the determination of dissociation (k_{off}) and association (k_{on}) rate constants for the unlabeled compounds, LSD, (+)-JRT, and 6-F-DET. Data represent the mean ± SEM of 2–4 biological replicates.

Acceptable	Borderline	Unacceptable

Liver Microsome
$T_{1/2}$ (% remaining at 60 min)

>30 min	10–30 min	<10 min

Human	>129 min (79%)
Mouse	29 min (44%)
Rat	64 min (57%)
Dog	>120 min (74%)
Cyno	31 min (27%)

Hepatocyte Clearance
$T_{1/2}$ (% remaining at 120 min)

>30 min	10–30 min	<10 min

Human	91 min (39%)
Mouse	37 min (15%)
Rat	107 min (48%)
Dog	>240 min (76%)
Cyno	59 min (19%)

CYP Isozyme Inhibition
IC_{50} (μM)

>10 μM	1–10 μM	<1 μM

3A4	>30 μM
2D6	>30 μM
1A2	>30 μM
2C9	>30 μM
2C19	>30 μM

Plasma Protein Binding
(% fraction unbound)

>5%	0.5–5%	<0.5%

Human	9%
Mouse	8%
Rat	11%
Dog	12%
Cyno	4%

CYP2D6 Utilization
$T_{1/2}$ (min)

>60 min	10–60 min	<10 min

>60 min

Brain Protein Binding
(% fraction unbound)

>0.5%	0.5–0.05%	<0.05%

Rat	9%
Mouse	9%

MDCK-MDR1
P_{app} A→B (cm/s)

>30 x 10^{-6}	5–30 x 10^{-6}	<5 x 10^{-6}

52 x 10^{-6}

hERG Inhibition
IC_{50} (μM)

>10 μM	1–10 μM	<1 μM

>30 μM

Figure S7. (+)-JRT exhibits excellent ADME properties and does not inhibit hERG channels. (+)-JRT does not exhibit any unacceptable ADME or hERG inhibition properties that would preclude it from clinical development.

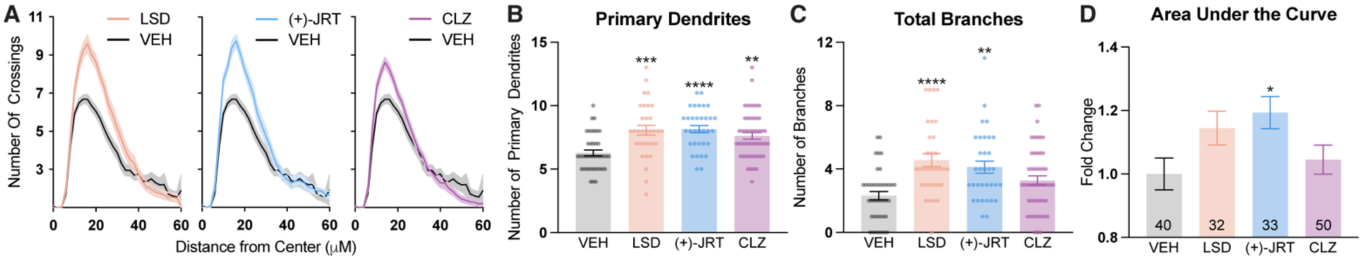

Figure S8. (+)-JRT promotes cortical neuritogenesis in vitro. (**A**) Sholl analysis of embryonic day 18 (E18) rat cortical neurons (DIV6) treated with compounds (1 μM) demonstrates that (+)-JRT increases dendritic arbor complexity in vitro. (**B**–**C**) Cortical neuronal cultures treated with LSD and (+)-JRT display an increase in the number of primary dendrites (**B**) and total branches (**C**), while CLZ only increases the number of primary dendrites. (**D**) Area under the curves of the Sholl plots in (**A**) demonstrates that JRT promotes growth compared to the VEH control. N values are shown within the bars. VEH = vehicle; LSD = lysergic acid diethylamide; CLZ = clozapine. *$p < 0.05$, **$p < 0.01$, ***$p < 0.001$, ****$p < 0.0001$, as compared to VEH control. See Methods and SI Appendix, Table S1 for full details on statistics.

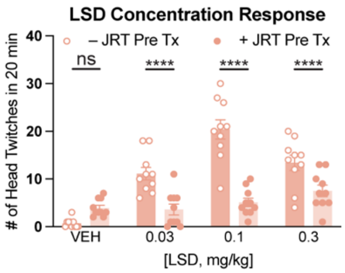

Figure S9. Pretreatment with (+)-JRT attenuates LSD-induced HTR. Pretreatment of (+)-JRT (1 mg/kg, IP) did not produce a significant HTR on its own, but attenuated the response induced by LSD (IP) at all doses tested. VEH = vehicle; LSD = lysergic acid diethylamide; ns = not significant. *p < 0.05, **p < 0.01, ***p < 0.001, ****p < 0.0001, as compared to the indicated comparator. See Methods and SI Appendix, Table S1 for full details on statistics.

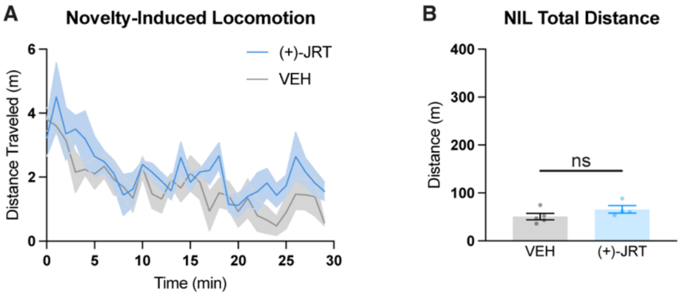

Figure S10 Administration of (+)-JRT does not impact novelty-induced locomotion. (**A**) Locomotor activity was tracked for 30 min in a novel open field following administration of (+)-JRT (1 mg/kg, IP) to male and female mice. (**B**) Quantification of the total distance traveled over 30 min indicated that (+)-JRT does not impact locomotion in the open field compared to the vehicle control. VEH = vehicle; ns = not significant.

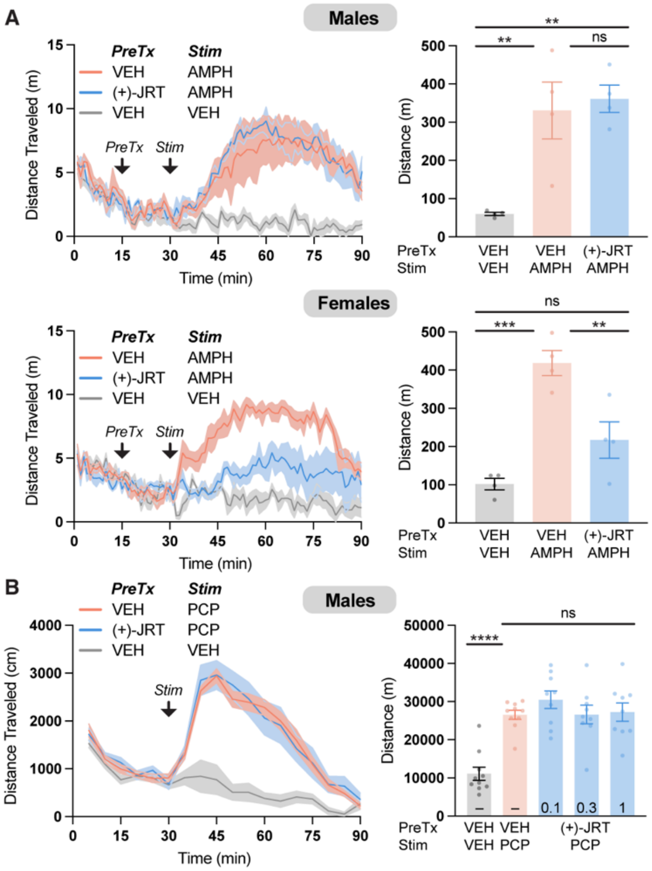

Figure S11. (+)-JRT does not exacerbate AMPH or PCP induced hyperactivity. (**A**) Pretreatment with (+)-JRT (1 mg/kg, IP) can block AMPH-induced hyperlocomotion in female, but not male, mice. (**B**) Pretreatment with (+)-JRT (1 mg/kg, IP) does not exacerbate PCP-induced hyperlocomotion in mice. AMPH = (+)-amphetamine; PCP = phencyclidine; VEH = vehicle; ns = not significant. *p < 0.05, **p < 0.01, ***p < 0.001, ****p < 0.0001, as compared to the comparator(s) indicated by a horizontal bar. See Methods and SI Appendix, Table S1 for full details on statistics.

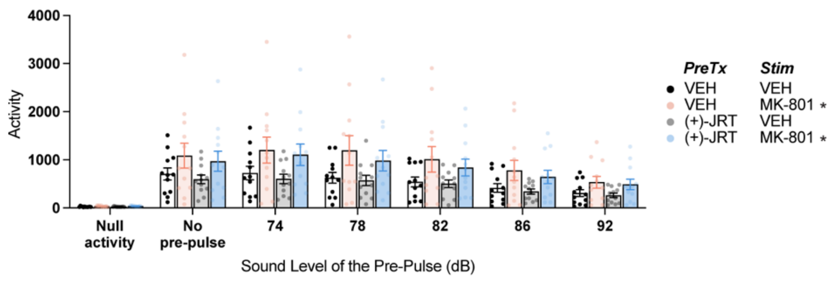

Figure S12. (+)-JRT does not impact startle response. Pretreating mice with (+)-JRT (1 mg/kg, IP) does not impact startle responses regardless of pre-pulse or stimulation with MK-801. A 3-way ANOVA indicated a significant main effect of sound level and MK-801 treatment, but not (+)-JRT treatment The only significant interaction effects was between sound level and MK-801 treatment. VEH = vehicle. *$p < 0.05$, **$p < 0.01$, ***$p < 0.001$, ****$p < 0.0001$. See Methods and SI Appendix, Table S1 for full details on statistics

(+)-JRT Does Not Promote SCZ-Related Gene Expression

Given that (+)-JRT produced robust changes in structural plasticity in the prefrontal cortex 24 h after a single administration (Figure 4H and J), we were interested in assessing gene expression at this time point (Figure S13A). We performed bulk mRNA sequencing of the prefrontal cortex 24 h after drug administration, yielding high quality expression data (Figure S14). Pairwise comparison of each drug treatment to vehicle identified differential expression signatures (Figure S13B), but relatively subtle differential expression effects overall (Figures S13C and D, SI Appendix, Dataset S2, note: Dataset S2 is an excel file of differential gene expression). While no single genes passed stringent adjusted p-value criteria, LSD and (+)-JRT treatments resulted in 198 and 114 differentially expressed genes (DEGs) compared to the VEH control at uncorrected $p < 0.01$, respectively, with log2 fold changes in gene expression generally between -1 and 1 (Figure S13C). For genes that were differentially expressed at $p < 0.01$, the directional change relative to vehicle was largely concordant between the two drugs (Figure S13D).

To assess differences in magnitude of expression change, we focused on genes with $p < 0.01$ and absolute log2 FC > 0.2 in one of the two drug treatments, categorizing these as downregulated or upregulated. No genes had log2 FC > 0.2 in one drug and log2 FC < -0.2 in the other. Of the 48 genes in the upregulated group and 45 in the downregulated group by LSD, 22 and 15 were in the same category in the (+)-JRT treatment experiment (Figure S13E). The remaining 26 upregulated and 30 downregulated genes did not pass the fold change threshold (sub-threshold category). Similarly, of the 18 DEGs in the upregulated group and 34 genes in the downregulated group by (+)-JRT, 7 and 16 were modulated in the same direction following LSD treatment, with the remainder exhibiting sub-threshold changes (Figure S13F). These data reflect an overall shared DEG signature, but with differences in gene-level response driven by the two drugs.

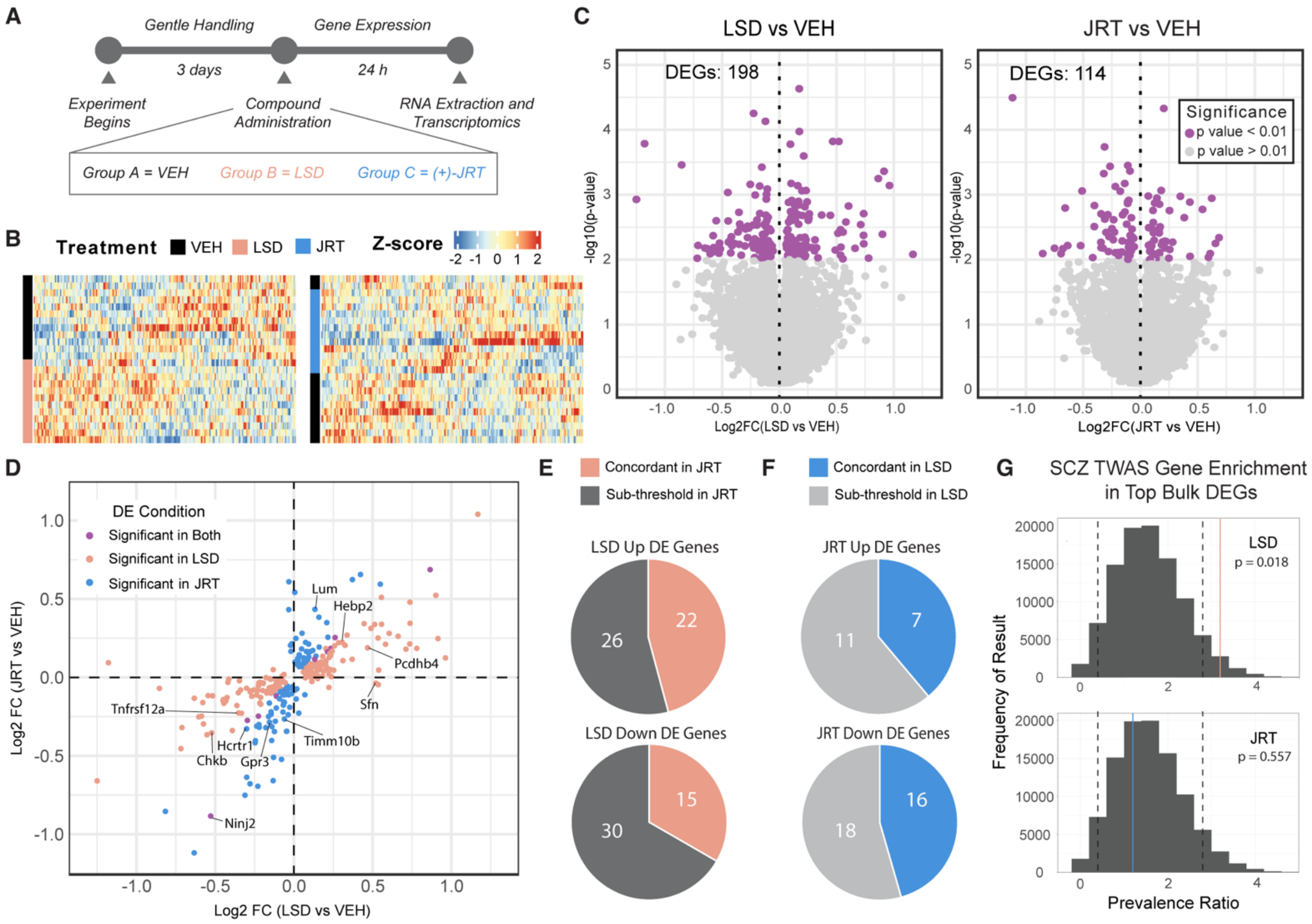

Figure S13. Transcriptomic responses in the prefrontal cortex after administration of LSD or (+)-JRT. (**A**) Mice (male and female) were gently handled for 3 days prior to compound administration (1 mg/kg, IP). After 24 h, RNA was extracted. (**B**) A heatmap shows subject level variation of DEGs. Clustering on both genes and samples demonstrates that each compound produces a distinct effect on gene expression. (**C**) Volcano plots demonstrate that relatively few genes are differentially regulated by LSD or (+)-JRT relative to the VEH control. Positive log2FC indicates higher expression in treatment relative to VEH. Purple dots on the plot and count in the upper left represent genes with unadjusted $p < 0.01$. (**D**) A scatter plot of log2FC for DEG ($p < 0.01$) shows the similarity of effect for each drug. The top 10 genes by magnitude of

FC that are present in a recent schizophrenia TWAS (*49*) are labeled. (**E–F**) Pie charts indicate which DEGs ($p < 0.01$) by LSD (E) or (+)-JRT (F) are modulated in the same direction by (+)-JRT and LSD, respectively. Genes were categorized as downregulated (log2FC < -0.2), upregulated (log2FC > 0.2), or sub-threshold (log2FC > -0.2 and < 0.2). No genes were differentially regulated by LSD and (+)-JRT in opposite directions. (**G**) Permutation tests show enrichment of the top 250 SCZ TWAS genes (FDR < 0.01 ranked by absolute log2FC) in the top 250 DEGs ($p < 0.1$, ranked by absolute log2FC) for each treatment. Dotted lines represent the 5th and 95th percentiles of the distribution for prevalence ratio of TWAS genes in randomly sampled gene sets. Orange and blue lines represent observed SCZ gene prevalence ratio for each drug. VEH = vehicle; DEG = differentially expressed gene; FC = fold change; FDR = false discovery rate. See also Figure S14.

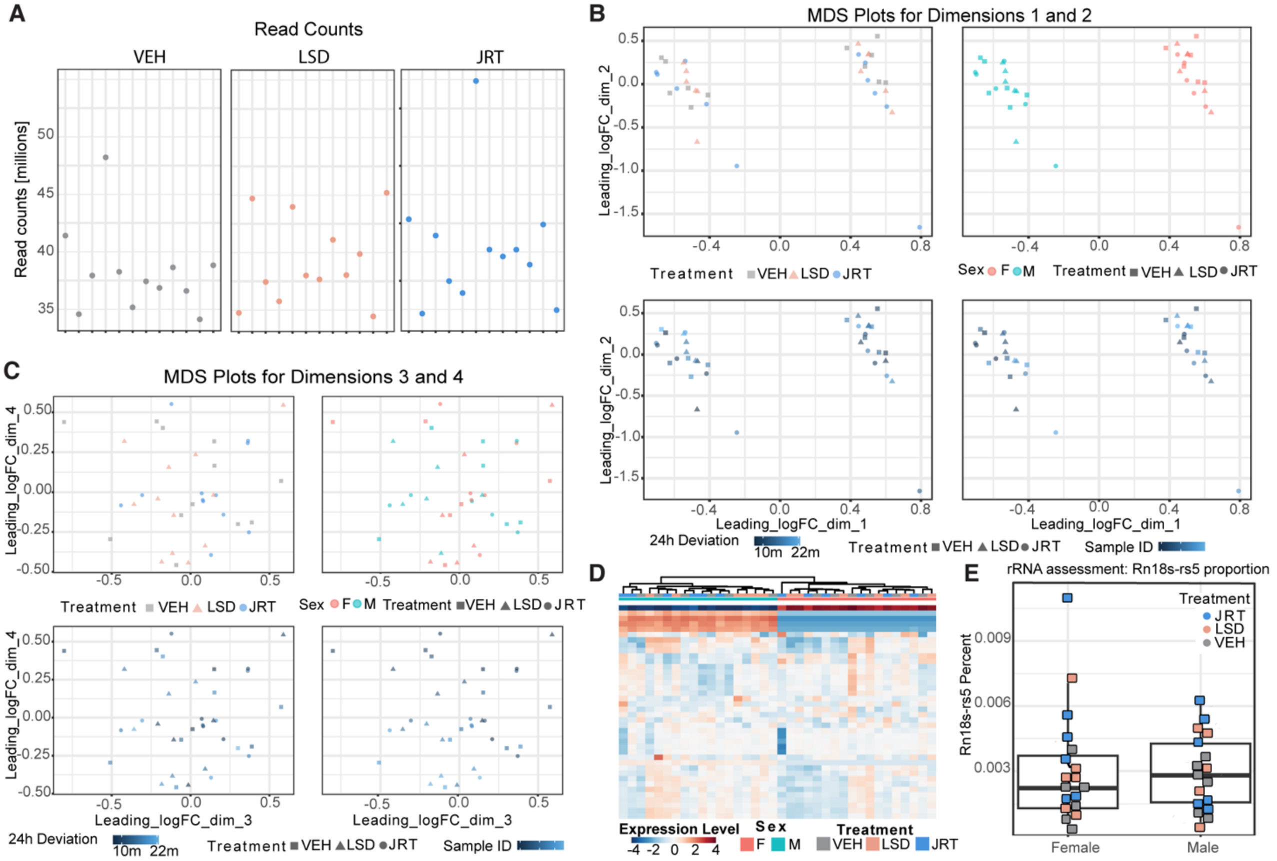

Figure S14. Transcriptomics quality control. (**A**) Samples show adequate sequencing depth and similar distribution of total RNA read counts for each biological replicate by treatment group prior to filtering. (**B–C**) Multidimensional scaling (MDS) plot of each biological replicate using the top 500 genes with highest standard deviation between samples shows that sex is strongest contributor to gene expression sample-level variance and separated along MDS dimension 1, with a weaker relationship between sample ID (processing order) and MDS dimension 4. There was no obvious separation among the top four dimensions for treatment group or sample collection time deviance (minutes) from the intended 24 h timepoint. The log2 fold change from mean MDS across samples of the first and second dimensions (B) or the third and fourth dimensions (C) are plotted on the x and y axis, respectively. Graphs were created using R (2016 release of the limma package v3.28.14). (**D**) Heatmap of top 40 variable genes across samples ordered by inter-individual variance shows highly variable genes are not treatment-associated and not driven by specific samples. Each column is a sample and each row is a gene, except for the top two rows which represent treatment and sex. Values are colored by row-level z-score of expression. Samples are grouped by the complete clustering method with euclidean distances. The samples cluster based on sex-linked gene expression (five genes at the top of plot), which have the highest overall variance due to male versus female differences. Graphs were created using R (2019 release of the pheatmap package v1.0.12). (**E**) The percent of total reads of each replicate that are mapped to Rn18s-rs5 are plotted, showing no systematic issues with ribosomal RNA.

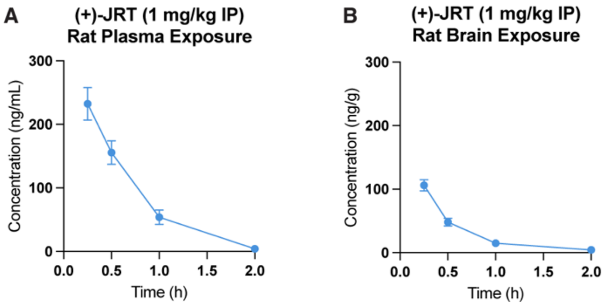

C

	Plasma	***Brain***
T_{max}	0.250 h	0.250 h
C_{max}	232 ng/mL	106 ng/g
$T_{1/2}$	0.282 h	0.446 h

Figure S15. Pharmacokinetic studies of (+)-JRT. (**A**) Time course showing the concentration of (+)-JRT in the plasma of rats following a 1 mg/kg IP administration of (+)-JRT fumarate (N = 3). (**B**) Time course showing the concentration of (+)-JRT in the brains of rats following a 1 mg/kg IP administration of (+)-JRT fumarate (N = 3 for the 0.25, 0.5, and 1.0 h time points, N = 1 for the 2.0 h time point). (**C**) Table depicting major estimated pharmacokinetic parameters. At 4 h, levels of (+)-JRT in either plasma or the brain were below the limit of quantification.

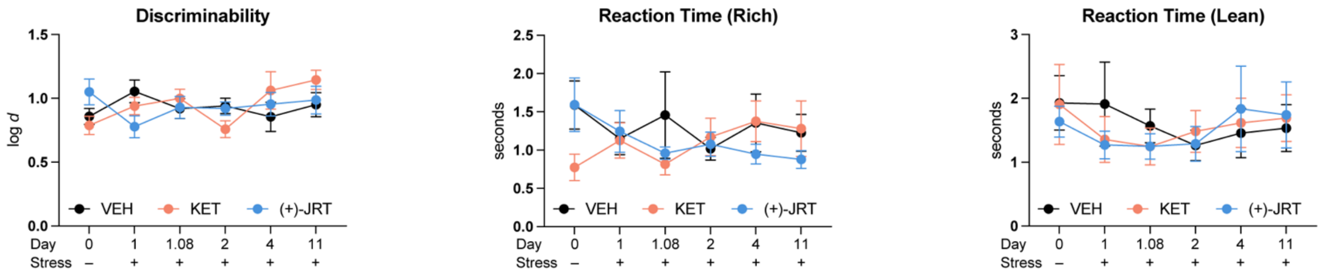

Figure S16. Discriminability and reaction times are stable over time in the probabilistic reward task. A 2-way ANOVA did not reveal any statistically significant effects of time or treatment for discriminability, reaction times for the rich trial types, or reaction times for the lean trial types. See Methods and SI Appendix, Table S1 for full details on statistics.

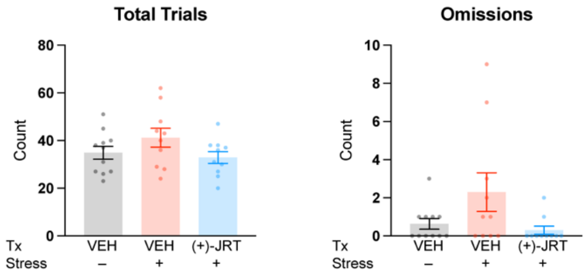

Figure S17. Total and omission trials for cognitive flexibility experiments. Data indicate the total number of trials each mouse completed (combining the discrimination and reversal periods, left) as well as the number of omissions during the reversal phase of the assay (right). No comparisons were statistically significant. See Methods and SI Appendix, Table S1 for full details on statistics.

Table S1. Statistical Parameters

Figure	Mean ± S.E.M	N	P value	Significance	F	t	Test method
**Figure 4B**		**Neurons**					
VEH vs. LSD	6.675 ± 0.2231 | 9.594 ± 0.4396	40 | 32	<0.0001	****	20.18		One-way ANOVA with Tukey's post hoc test
VEH vs. (+)-JRT	6.675 ± 0.2231 | 9.758 ± 0.3400	40 | 33	<0.0001	****			
VEH vs. CLZ	6.675 ± 0.2231 | 8.620 ± 0.2554	40 | 50	<0.0001	****			
LSD vs. (+)-JRT	9.594 ± 0.4396 | 9.758 ± 0.3400	32 | 33	0.986	ns			
LSD vs. CLZ	9.594 ± 0.4396 | 8.620 ± 0.2554	32 | 50	0.1181	ns			
(+)-JRT vs. CLZ	9.758 ± 0.3400 | 8.620 ± 0.2554	33 | 50	0.0451	*			
**Figure 4D**		**Neurons**					
VEH vs. LSD	3.192 ± 0.1825 | 4.396 ± 0.2199	26 | 28	0.0002	***	15.94		One-way ANOVA with Tukey's post hoc test
VEH vs. (+)-JRT	3.192 ± 0.1825 | 5.034 ± 0.1860	26 | 31	<0.0001	****			
VEH vs. CLZ	3.192 ± 0.1825 | 3.959 ± 0.1775	26 | 30	0.0322	*			
LSD vs. (+)-JRT	4.396 ± 0.2199 | 5.034 ± 0.1860	28 | 31	0.0875	ns			
LSD vs. CLZ	4.396 ± 0.2199 | 3.959 ± 0.1775	28 | 30	0.3757	ns			
(+)-JRT vs. CLZ	5.034 ± 0.1860 | 3.959 ± 0.1775	31 | 30	0.0005	***			
**Figure 4E**		**Neurons**					
Interaction	–		0.0007	***	7.905		Two-way ANOVA with Šídák's
Blocking	–		<0.0001	****	20.09		

Treatment	–		0.0548	ns	3.006		multiple comparisons test
VEH + (+)-JRT vs. VEH + LSD	11.789 ± 0.560 | 11.041 ± 0.608	15 | 15	0.7663	ns		0.8751	
VEH + (+)-JRT vs. VEH	11.789 ± 0.560 | 8.116 ± 0.791	15 | 15	0.0001	***		4.295	
VEH + LSD vs. VEH	11.041 ± 0.608 | 8.116 ± 0.791	15 | 15	0.0029	**		3.420	
KETSN + (+)-JRT vs. KETSN + LSD	7.839 ± 0.492 | 7.822 ± 0.451	15 | 15	>0.9999	ns		0.01959	
KETSN + (+)-JRT vs. KETSN + VEH	7.839 ± 0.492 | 8.648 ± 0.662	15 | 15	0.7213	ns		0.9461	
KETSN + LSD vs. KETSN + VEH	7.822 ± 0.451 | 8.648 ± 0.662	15 | 15	0.7085	ns		0.9657	
(+)-JRT + VEH vs. KETSN + VEH	11.789 ± 0.560 | 7.839 ± 0.492	15 | 15	<0.0001	****		4.620	
LSD + VEH vs. KETSN + VEH	11.041 ± 0.608 | 7.822 ± 0.451	15 | 15	0.0003	***		3.764	
VEH + VEH vs. KETSN + VEH	8.116 ± 0.791 | 8.648 ± 0.662	15 | 15	0.5361	ns		0.6212	
**Figure 4H**		**Fields**					
VEH vs. (+)-JRT	13.43 ± 0.8030 | 19.58 ± 1.606	24 | 24	0.0153	*			Unpaired Mann-Whitney test
**Figure 4J**		**Fields**					
VEH vs. (+)-JRT	1.435 ± 0.06204 | 1.690 ± 0.04906	18 | 18	0.0028	**	1.599	3.225	Unpaired t test
**Figure 4K**		**Fields**					
VEH vs. (+)-JRT	0.003441 ± 0.0001939 | 0.003639 ± 0.0001362	18 | 18	0.41	ns	2.027	0.8342	Unpaired t test
**Figure 4N**		**Mice**					
Interaction	–		0.0197	*	5.933		Two-way ANOVA with uncorrected
Stress	–		0.0016	**	11.48		

Treatment	–		0.2706	ns	1.250		Fisher's LSD
GH<br>VEH vs. JRT	12.17 ± 0.5278 | 11.36 ± 0.8451	11 | 10	0.3551	Ns		0.9361	
CORT<br>VEH vs. JRT	8.570 ± 0.4350 | 10.77 ± 0.5664	9 | 12	0.0168	*		2.501	
VEH<br>GH vs. CORT	12.17 ± 0.5278 | 8.570 ± 0.4350	11 | 9	0.0003	***		4.022	
JRT<br>GH vs. CORT	11.36 ± 0.8451 | 10.77 ± 0.5664	10 | 12	0.4938	ns		0.6909	
**Figure 5A Agonism**		**Mice**					
					4.954		One-way ANOVA with Dunnett's post hoc test (compared to VEH)
(+)-JRT 1mg/kg vs VEH	1.938 ± 0.5545 | 1 ± 0.2315	8 | 8	0.9606	ns			
(+)-JRT 0.2mg/kg vs VEH	2.75 ± 0.8504 | 1 ± 0.2315	8 | 8	0.6323	ns			
(+)-JRT 0.04mg/kg vs VEH	1.438 ± 0.3946 | 1 ± 0.2315	8 | 8	0.9992	ns			
LSD 1mg/kg vs VEH	5.5 ± 0.5089 | 1 ± 0.2315	8 | 8	0.0098	**			
LSD 0.2mg/kg vs VEH	6.375 ± 2.195 | 1 ± 0.2315	8 | 8	0.0015	**			
LSD 0.04mg/kg vs VEH	1.286 ± 0.3595 | 1 ± 0.2315	7 | 8	>0.9999	ns			
**Figure 5A Antagonism**		**Mice**					
VEH + VEH vs VEH + LSD	1 ± 0.2315 | 6.786 ± 0.8854	8 | 7	<0.0001	****	34.37		One-way ANOVA with Tukey's post hoc test
VEH + VEH vs (+)-JRT + LSD	1 ± 0.2315 | 1.500 ± 0.3450	8 | 7	0.79	ns			
VEH + LSD vs (+)-JRT + LSD	6.786 ± 0.8854 | 1.500 ± 0.3450	7 | 7	<0.0001	****			
**Figure 5B**		**Mice**					
dB	–	12	<0.0001	****	106.7		Three-way ANOVA
JRT	–	12	0.3860	ns	0.7666		
MK801	–	12	0.0038	**	9.369		
dB x JRT	–	12	0.9888	ns	0.07856		
dB x MK801	–	12	0.7943	ns	0.4197		
JRT x MK801	–	12	0.9281	ns	0.008236		

dB x JRT x MK801	–	12	0.8329	ns	0.3655		
**Figure 6B Immobility**		**Rats**					
VEH vs Ketamine 10mg/kg	37.20 ± 1.867 | 25.00 ± 1.265	10 | 10	<0.0001	****			
VEH vs (+)-JRT 0.1mg/kg	37.20 ± 1.867 | 28.70 ± 1.334	10 | 10	0.0072	**	6.569		One-way ANOVA with Dunnett's post hoc test (compared to VEH)
VEH vs (+)-JRT 0.3mg/kg	37.20 ± 1.867 | 27.20 ± 2.546	10 | 10	0.0014	**			
VEH vs (+)-JRT 1.0mg/kg	37.20 ± 1.867 | 27.70 ± 1.850	10 | 10	0.0024	**			
**Figure 6B Swimming**		**Rats**					
VEH vs Ketamine 10mg/kg	22.10 ± 1.929 | 34.10 ± 1.187	10 | 10	0.0001	***			
VEH vs (+)-JRT 0.1mg/kg	22.10 ± 1.929 | 29.60 ± 1.352	10 | 10	0.0194	*	6.372		One-way ANOVA with Dunnett's post hoc test (compared to VEH)
VEH vs (+)-JRT 0.3mg/kg	22.10 ± 1.929 | 31.70 ± 2.534	10 | 10	0.002	**			
VEH vs (+)-JRT 1.0mg/kg	22.10 ± 1.929 | 31.50 ± 1.778	10 | 10	0.0025	**			
**Figure 6D**		**Mice**					
Baseline vs. post-CORT	0.7980 ± 0.03533 | 0.5563 ± 0.08424	20	0.0074	**		2.997	Paired t test
**Figure 6E**		**Mice**					
Time x Treatment Group	–	9	0.6260	ns	0.6584		
Time	–	9	0.0077	**	4.307		Two-way repeated measures ANOVA
Treatment Group	–	9	0.0364	*	6.664		
**Figure 6G**		**Mice**					
Time x Treatment Group	–	8	0.1217	Ns	1.583		
Time	–	8	<0.0001	****	8.928		
Treatment Group	–	8	0.0617	ns	3.191		Two-way repeated measures ANOVA with Dunnett's post hoc test
JRT Day 1 vs. Day 0	0.131 ± 0.082 |	8	0.0005	***			

	0.453 ± 0.027						
JRT Day 1 vs. Day 1.08	0.131 ± 0.082 | 0.491 ± 0.071	8	<0.0001	****			
JRT Day 1 vs. Day 2	0.131 ± 0.082 | 0.406 ± 0.069	8	0.0034	**			
JRT Day 1 vs. Day 3	0.131 ± 0.082 | 0.474 ± 0.112	8	0.0002	***			
JRT Day 1 vs. Day 11	0.131 ± 0.082 | 0.290 ± 0.061	8	0.1728	ns			
VEH Day 1 vs. Day 0	0.167 ± 0.033 | 0.386 ± 0.031	8	0.0280	*			
VEH Day 1 vs. Day 1.08	0.167 ± 0.033 | 0.199 ± 0.061	8	0.9925	ns			
VEH Day 1 vs. Day 2	0.167 ± 0.033 | 0.227 ± 0.052	8	0.9071	ns			
VEH Day 1 vs. Day 3	0.167 ± 0.033 | 0.279 ± 0.038	8	0.4756	ns			
VEH Day 1 vs. Day 11	0.167 ± 0.033 | 0.203 ± 0.052	8	0.9879	ns			
KET Day 1 vs. Day 0	0.120 ± 0.042 | 0.343 ± 0.037	8	0.0247	*			
KET Day 1 vs. Day 1.08	0.120 ± 0.042 | 0.457 ± 0.070	8	0.0002	***			
KET Day 1 vs. Day 2	0.120 ± 0.042 | 0.330 ± 0.035	8	0.0381	*			
KET Day 1 vs. Day 3	0.120 ± 0.042 | 0.304 ± 0.091	8	0.0861	Ns			
KET Day 1 vs. Day 11	0.120 ± 0.042 | 0.266 ± 0.086	8	0.2366	ns			
**Figure 7B Discrimination**		**Mice**					

VEH – Stress vs VEH + Stress	21.27 ± 2.516 | 20.20 ± 1.843	11 | 10	0.9377	ns	0.06302			One-way ANOVA with Tukey's post hoc test
VEH – Stress vs (+)-JRT + Stress	21.27 ± 2.516 | 20.50 ± 2.212	11 | 10	0.9671	ns				
VEH + Stress vs (+)-JRT + Stress	20.20 ± 1.843| 20.50 ± 2.212	10 | 10	0.9952	ns				
**Figure 7B Reversal**		**Mice**						
VEH – Stress vs VEH + Stress	13.64 ± 1.744 | 21.00 ± 2.385	11 | 11	0.0200	*	6.371			One-way ANOVA with Tukey's post hoc test
VEH – Stress vs (+)-JRT + Stress	13.64 ± 1.744 | 12.40 ± 1.118	11 | 10	0.8796	ns				
VEH + Stress vs (+)-JRT + Stress	21.00 ± 2.385| 12.40 ± 1.118	10 | 10	0.0075	**				
**Figure S8B**		**Neurons**						
VEH vs. LSD	6.275 ± 0.2292 | 8.063 ± 0.3835	40 | 32	0.0002	***	8.969			One-way ANOVA with Tukey's post hoc test
VEH vs. (+)-JRT	6.275 ± 0.2292 | 8.152 ± 0.2824	40 | 33	<0.0001	****				
VEH vs. CLZ	6.275 ± 0.2292 | 7.620 ± 0.2586	40 | 50	0.0027	**				
LSD vs. (+)-JRT	8.063 ± 0.3835 | 8.152 ± 0.2824	32 | 33	0.9971	ns				
LSD vs. CLZ	8.063 ± 0.3835 | 7.620 ± 0.2586	32 | 50	0.6894	ns				
(+)-JRT vs. CLZ	8.152 ± 0.2824 | 7.620 ± 0.2586	33 | 50	0.5422	ns				
**Figure S8C**		**Neurons**						
VEH vs. LSD	2.325 ± 0.2641 | 4.563 ± 0.4089	40 | 32	<0.0001	****	8.499			One-way ANOVA with Tukey's post hoc test
VEH vs. (+)-JRT	2.325 ± 0.2641 | 4.121 ± 0.3838	40 | 33	0.0015	**				

VEH vs. CLZ	2.325 ± 0.2641 | 3.280 ± 0.2858	40 | 50	0.1269	ns				
LSD vs. (+)-JRT	4.563 ± 0.4089 | 4.121 ± 0.3838	32 | 33	0.82	ns				
LSD vs. CLZ	4.563 ± 0.4089 | 3.280 ± 0.2858	32 | 50	0.0314	*				
(+)-JRT vs. CLZ	4.121 ± 0.3838 | 3.280 ± 0.2858	33 | 50	0.2607	ns				
**Figure S8D**		**Neurons**						
VEH vs. LSD	1.000 ± 0.05010 | 1.144 ± 0.05330	40 | 32	0.2074	ns	3.008		One-way ANOVA with Tukey's post hoc test	
VEH vs. (+)-JRT	1.000 ± 0.05010 | 1.193 ± 0.05057	40 | 33	0.0437	*				
VEH vs. CLZ	1.000 ± 0.05010 | 1.045 ± 0.04563	40 | 50	0.9027	ns				
LSD vs. (+)-JRT	1.144 ± 0.05330 | 1.193 ± 0.05057	32 | 33	0.9199	ns				
LSD vs. CLZ	1.144 ± 0.05330 | 1.045 ± 0.04563	32 | 50	0.4932	ns				
(+)-JRT vs. CLZ	1.193 ± 0.05057 | 1.045 ± 0.04563	33 | 50	0.1481	ns				
**Figure S9**		**Mice**						
Interaction	-		<0.0001	****	18.68		Two-way ANOVA with Šídák's multiple comparisons test	
Concentration	-		<0.0001	****	26.49			
Pretreatment	-		<0.0001	****	52.70			
VEH -JRT PreTx vs. +JRT Pre Tx	0.7 ± 0.3 | 3.9 ± 0.567	10 | 10	0.2603	Ns		1.822		
LSD (0.03 mg/kg) -JRT PreTx vs. +JRT Pre Tx	11.1 ± 1.32 | 3.6 ± 1.147	10 | 10	0.0002	***		4.270		
LSD (0.1 mg/kg) -JRT PreTx vs. +JRT Pre Tx	20.4 ± 2.012 | 5.1 ± 0.888	10 | 10	<0.0001	****		8.711		
LSD (0.3 mg/kg) -JRT PreTx vs. +JRT Pre Tx	13.4 ± 1.607 | 7.5 ± 1.204	10 | 10	0.0050	**		3.359		

Figure S10B		Mice					
VEH vs (+)-JRT		5 | 4	0.1772	ns	1.110	1.500	Unpaired t test
**Figure 11A Males**		**Mice**					
VEH + VEH vs VEH + AMPH	59.80 ± 4.206 | 330.5 ± 74.31	4 | 4	0.0076	**	12.13		One-way ANOVA with Tukey's post hoc test
VEH + VEH vs (+)-JRT + AMPH	59.80 ± 4.206 | 361.2 ± 35.54	4 | 4	0.004	**			
VEH + AMPH vs (+)-JRT + AMPH	330.5 ± 74.31 | 361.2 ± 35.54	4 | 4	0.893	ns			
**Figure 11A Females**		**Mice**					
VEH + VEH vs VEH + AMPH	101.9 ± 15.04 | 418.5 ± 32.97	4 | 4	0.0003	***	21.47		One-way ANOVA with Tukey's post hoc test
VEH + VEH vs (+)-JRT + AMPH	101.9 ± 15.04 | 217.2 ± 47.70	4 | 4	0.0982	ns			
VEH + AMPH vs (+)-JRT + AMPH	418.5 ± 32.97 | 217.2 ± 47.70	4 | 4	0.0066	**			
**Figure 11B**		**Mice**					
VEH + VEH vs. VEH + PCP	11101 ± 1709 | 26552 ± 1179	10 | 10	<0.0001	****	14.75		One-way ANOVA with Tukey's post hoc test
VEH + VEH vs. (+)-JRT (0.1)+ PCP	11101 ± 1709 | 30482 ± 2310	10 | 9	<0.0001	****			
VEH + VEH vs. (+)-JRT (0.3)+ PCP	11101 ± 1709 | 26618 ± 2425	10 | 9	<0.0001	****			
VEH + VEH vs. (+)-JRT (1)+ PCP	11101 ± 1709 | 27253 ± 2401	10 | 9	<0.0001	****			
VEH + PCP vs. (+)-JRT (0.1)+ PCP	26552 ± 1179 | 30482 ± 2310	10 | 9	0.6428	ns			
VEH + PCP vs. (+)-JRT (0.3)+ PCP	26552 ± 1179 | 26618 ± 2425	10 | 9	>0.9999	ns			
VEH + PCP vs. (+)-JRT (1)+ PCP	26552 ± 1179 | 27253 ± 2401	10 | 9	0.9992	ns			

(+)-JRT (0.1)+ PCP vs. (+)-JRT (0.3)+ PCP	30482 ± 2310 | 26618 ± 2425	9 | 9	0.6783	ns			
(+)-JRT (0.1)+ PCP vs. (+)-JRT (1)+ PCP	30482 ± 2310 | 27253 ± 2401	9 | 9	0.8024	ns			
(+)-JRT (0.3)+ PCP vs. (+)-JRT (1)+ PCP	26618 ± 2425 | 27253 ± 2401	9 | 9	0.9995	ns			
**Figure S12**		**Mice**					
dB	–	12	<0.0001	****	61.59		Three-way ANOVA
JRT	–	12	0.5262	ns	0.4082		
MK801	–	12	0.0187	*	5.965		
dB x JRT	–	12	0.9065	ns	0.3552		
dB x MK801	–	12	0.0002	***	4.471		
JRT x MK801	–	12	0.8613	ns	0.03087		
dB x JRT x MK801	–	12	0.9745	ns	0.2066		
**Figure S16 Discriminability**		**Mice**					
Time x Treatment Group	–	8	0.0313	*	2.093		Two-way ANOVA
Time	–	8	0.2774	ns	1.281		
Treatment Group	–	8	0.9544	ns	0.04681		
**Figure S16 Reaction Time (Rich)**		**Mice**					
Time x Treatment Group	–	8	0.1250	ns	1.572		Two-way ANOVA
Time	–	8	0.7519	ns	0.5316		
Treatment Group	–	8	0.6788	ns	0.3947		
**Figure S16 Reaction Time (Lean)**		**Mice**					
Time x Treatment Group	–	8	0.8808	ns	0.5085		Two-way ANOVA
Time	–	8	0.3264	ns	1.175		
Treatment Group	–	8	0.9679	ns	0.03272		
**Figure S17 Total Trials**		**Mice**					
VEH vs. Stress	34.91 ± 2.661 | 41.20 ± 3.972	11 | 10	0.3287	ns			One-way ANOVA with Tukey's post hoc test
VEH vs. Stress + (+)-JRT	34.91 ± 2.661 | 32.90 ± 2.479	11 | 10	0.8886	ns			
Stress vs. Stress + (+)-JRT	41.20 ± 3.972 | 32.90 ± 2.479	10 | 10	0.1655	ns			
**Figure S17 Omissions**		**Mice**					

VEH vs. Stress	0.6364 ± 0.2787 | 2.3 ± 1.012	11 | 10	0.1388	ns			One-way ANOVA with Tukey's post hoc test
VEH vs. Stress + (+)-JRT	0.6364 ± 0.2787 | 0.3 ± 0.2134	11 | 10	0.9166	ns			
Stress vs. Stress + (+)-JRT	2.3 ± 1.012 | 0.3 ± 0.2134	10 | 10	0.0705	ns			

Dataset S1. Radioligand Binding Studies for LSD. The associated .xlsx file contains literature K_i values for LSD binding to various target receptors. The radioligand employed, receptor species, and titles of the associated references are also shown.

Dataset S2. Gene Expression Data for LSD and (+)-JRT. The associated .csv file contains information on fold change and *p* values for differentially expressed genes in the mPFC 24 h after treatment (1 mg/kg, IP) with LSD or (+)-JRT.

Materials and Methods

Data Analysis and Statistics. Treatments were randomized, and data were analyzed by experimenters blinded to treatment conditions. Statistical analyses were performed using GraphPad Prism (version 10.0.3) unless noted otherwise. All comparisons were planned prior to performing each experiment. No data were excluded. Data are represented as mean ± SEM, unless noted otherwise, with asterisks indicating *$p < 0.05$, **$p < 0.01$, ***$p < 0.001$, and ****$p < 0.0001$. Details of all statistical tests are shown in SI Appendix, Table S1.

Molecular Docking. Docking was performed using the program Autodock Vina (version 1.2.3) (*1*) to dock (+)-JRT, (–)-JRT, and (+)-LSD into the 5-HT2A receptors (PDB: 6WGT, 7WC6). In each case, the existing bound (+)-LSD ligand and extraneous molecules provided in the published structure were removed from the protein. For 6WGT, only the central protein copy was used. The bound (+)-LSD ligand (for RMSD comparison of atomic coordinates of docked poses) was similarly obtained from the 5-HT2A receptor crystal structure. The (+)-JRT, (–)-JRT, and (+)-LSD PDB structures used were generated by conversion of ChemDraw structures to PDB files using UCSF ChimeraX (version 1.5), developed by the Resource for Biocomputing, Visualization, and Informatics at the University of California, San Francisco, with support from National Institutes of Health R01-GM129325 and the Office of Cyber Infrastructure and Computational Biology, National Institute of Allergy and Infectious Diseases (*2,3*). Requisite ligand and receptor PDBQT files for docking via Autodock Vina were generated by conversion of PDB files to PDBQT files using AutoDockTools (version 1.5.7) with polar hydrogens added. All ligand PDB files were assigned a common carbon, oxygen, and nitrogen numbering scheme via text editor prior to docking, which is necessary for accurate RMSD scoring of output poses. All docked poses of the ligands were compared to the bound (+)-LSD ligand by non-hydrogen RMSD calculation in AutoDockTools, and the lowest RMSD values were considered the most viable ligand poses, shown in the representative images. The ligands (+)-JRT, (–)-JRT, and (+)-LSD were docked according to the following parameters: The binding pocket search region was defined as a 40 x 40 x 40 grid with a spacing of 0.375 Å at coordinates x = 7, y = 3, z = 51 for 6WGT, and x = -28, y = -11, z = 142 for 7WC6 (centered over the bound (+)-LSD location in either structure) as designated in AutoDockTools with an exhaustiveness setting of 20. An improved set of empirical parameters (*4*) were used as follows: Gauss1 = -0.049811, Gauss2 = -0.007218, Repulsion = 0.756221, Hydrophobic = -0.469951, Rotation = 0.025722. Generated conformations were analyzed and exported using AutoDockTools. Three dimensional structures and images were produced using UCSF ChimeraX.

Drugs. Many of the drugs used in these studies were purchased from commercial sources including (+)-amphetamine sulfate (Sigma Aldrich, 1180004), ketamine hydrochloride (Spectrum, K1068), and ketanserin (APExBIO, B2248). Lysergic acid diethylamide (LSD) hemitartrate was generously provided by the NIH Drug Supply Program. Both (+)-JRT and (–)-JRT were synthesized in-house and judged to be analytically pure based on NMR and LC-MS data. For cell culture experiments, VEH = 0.1% (agonist studies) or 0.2% (antagonist studies) molecular biology grade dimethyl sulfoxide (Sigma-Aldrich). For in vivo experiments, compounds were administered i.p. at 5 mL/kg using 0.9% saline as the vehicle, unless noted otherwise. VEH = USP grade saline (0.9%). Stock solutions for behavioral assays were prepared fresh before use. Fumarate salts of (+)-JRT and (–)-JRT were used for all biological studies unless noted otherwise.

Animals. All experimental procedures involving animals were approved by the Institutional Animal Care and Use Committee (IACUC) at the University of California, Davis, the Salk Institute, Weill Cornell Medicine, McLean Hospital (Harvard Medical School), or the Contract Research Organization (CRO) where the study was performed. All procedures involving animals adhered to principles described in the National Institutes of Health Guide for the Care and Use of Laboratory Animals. Animals were either obtained from Jackson Laboratory (Sacramento, C.A.) or bred in-house unless noted otherwise. Power analyses were conducted to ensure appropriate sample sizes for all experiments involving animals. Animals were housed 2–5 animals of the same sex per cage on a 12h light/dark cycle and were given ad libitum access to food and water unless noted otherwise. The University of California, Davis, the Salk Institute, and Weill Cornell Medicine are accredited by the Association for Assessment and Accreditation of Laboratory Animal Care International (AAALAC). Whenever possible, both sexes were utilized. However, a single sex was utilized in some cases when 1) a contract research organization had only validated the assay with a single sex, or 2) previous data indicated that both sexes respond similarly in the assay.

Radioligand Binding Selectivity Panel. Competitive radioligand binding studies for (+)-JRT (10 μM) and (–)-JRT (10 μM) were performed across a panel of receptors at Eurofins Discovery. Experiments were performed using the free bases of (+)-JRT and (–)-JRT. LSD K_i values were obtained from previous reports, and whenever possible, were matched for radioligand and source of receptor (*5,6,7,8,9,10,11,12*). These data are shown in SI Appendix, Dataset S1.

Radioligand Binding Assays (5-HT2AR and 5-HT2CR). The 5-HT2AR and 5-HT2CR competitive radioligand binding assays were performed at Epics Therapeutics S.A. (Belgium, FAST-0505B) using conventional methods. Experiments were performed using the free bases of (+)-JRT and (–)-JRT. Briefly, competition binding was performed in duplicate in the wells of a 96-well plate (Master Block, Greiner, 786201) containing binding buffer, membrane extracts, radiotracer [^{3}H]-DOI and test compound. Nonspecific binding was determined by co-incubation with 200-fold excess of cold competitor DOI. The samples were incubated in a final volume of 0.1 mL at a temperature and for a duration optimized for either the 5-HT2AR

or 5-HT2CR and then filtered over filter plates. Filters were washed six times with 0.5 ml of ice-cold washing buffer (optimized for 5-HT2AR) and 50 µl of Microscint 20 (Packard) were added in each well. The plates were incubated for 15 min on an orbital shaker and then counted with a TopCountTM for 1 min/well.

Radioligand Binding Assays (5-HT2BR). The 5-HT2BR competitive radioligand binding assays were performed at Eurofins Cerep SA (Celle l'Evescault, France) using conventional methods (Catalog #1333) as described previously.[13] Experiments were performed using [^{125}I]-()-DOI and the free bases of (+)-JRT and (–)-JRT.

IP1 Assay (5-HT2AR and 5-HT2CR). The 5-HT2AR and 5-HT2CR IPOne HTRF assays were performed at Epics Therapeutics S.A. (Belgium, FAST-0505I) using conventional methods. Experiments were performed using the free bases of (+)-JRT and (–)-JRT. Briefly, CHO-K1 cells expressing human recombinant 5-HT2AR grown to mid-log phase in culture media without antibiotics were detached with PBS-EDTA, centrifuged, and resuspended in medium without antibiotics buffer. Then, 20,000 cells were distributed in a 96-well plate and incubated overnight at 37ºC with 5% CO_2. For agonist testing, the medium was removed and 20 µl of assay buffer plus 20 µl of test compound or reference agonist (α-Me-5-HT) were added to each well. The plate was incubated for 60 min at 37ºC with 5% CO_2. After addition of the lysis buffer containing IP1-d2 and anti-IP1 cryptate detection reagents, plates were incubated for 1 h at room temperature and fluorescence ratios were measured according to the manufacturer's specifications using the HTRF kit.

IP1 Assay (5-HT2BR). The 5-HT2BR IP1 assays were performed at Eurofins Cerep SA (Celle l'Evescault, France) using conventional methods (Catalog #3344) as described previously.[14] Experiments were performed using the free bases of (+)-JRT and (–)-JRT. Briefly, CHO cells transfected with the human 5-HT2B receptor were suspended in a buffer containing 10 mM Hepes/NaOH (pH 7.4), 4.2 mM KCl, 146 mM NaCl, 1 mM $CaCl_2$, 0.5 mM $MgCl_2$, 5.5 mM glucose and 50 mM LiCl. Then the cells were distributed in microplates at a density of ~2.104 cells/well and incubated for 30 min at 37°C in the presence of buffer (basal control), test compound, or reference agonist. For stimulated control measurement, separate assay wells contain 1 µM 5-HT were used. Following incubation, the cells are lysed and the fluorescence acceptor (D2-labeled IP1) and fluorescence donor (anti-IP1 antibody labeled with europium cryptate) were added. After 60 min at room temperature, the fluorescence transfer was measured at λex = 337 nm and λem = 620 and 665 nm using a microplate reader (Envision, Perkin Elmer). The IP1 concentration was determined by dividing the signal measured at 665 nm by that measured at 620 nm (ratio). The results are expressed as a percent of the control response to 1 µM 5-HT.

β-Arrestin Activation (PathHunter®). The 5-HT2AR PathHunter® β-arrestin agonist assay was performed at Eurofins DiscoverX (Frement, CA, Catalog # 86-0001P-2090AG). Experiments were performed using the free bases of (+)-JRT and (–)-JRT. The PathHunter® β-arrestin assay monitors the activation of a GPCR in a homogenous, non-imaging assay format using a technology developed by DiscoverX called Enzyme Fragment Complementation (EFC) with β-galactosidase (β-Gal) as the functonal reporter. The enzyme is split into two inactive complementary portions: a small peptide, called ProLink™ (PK) and a larger protein, called Enzyme Acceptor (EA). PK and EA are then expressed as fusion proteins in U2OS cells, with PK fused to the GPCR of interest, and EA fused to β-arrestin. When the target GPCR is activated and β-arrestin is recruited to the receptor, PK and EA complementation occurs, restoring β-Gal activity which is measured using chemiluminescent PathHunter® Detection Reagents.

BRET-Based GPCR Assays. HEK293T cells were seeded into 50 µg/mL poly-D-lysine-coated white optical bottom 96-well Nunclon Delta plates at 35,000 cells/well and incubated in 200 µL of DMEM + 10% FBS for 48 h at 37℃ and 5% CO_2. Transfection complexes were prepared at component ratios of 9 µL optiMEM:100 µg total plasmid mix:0.3 µL TransIT®-293 reagent per well, where the total plasmid mix was made up of a 1:1:1:1 mixture of 5-HT2A-mcherry, Gγ9-GFP2, Gβ3, and either G**α**q-rLuc8, G**α**i1-rLuc8, or G**α**i3-rLuc8 containing plasmids. Media was replaced with 92 µL of fresh pre-warmed DMEM + 10% FBS and 8 µL/well transfection complex was added. Plates were incubated for 48 h at 37℃ and 5% CO_2 prior to performing the assay. Next, plates were washed 3 x 100 µL HBSS and replaced with 80 µL/well of assay buffer (HBSS + 5 mM HEPES, pH = 7.4). Cells were treated with 10 µL/well of compound stock solutions to produce varying concentrations of serotonin and/or (+)-JRT or LSD (final solutions contained 0.1% DMSO) and incubated for 55 minutes at 37℃ and 5% CO_2 prior to addition of RLuc substrate prolume purple (10 µL/well of a 10x stock solution consisting of a 1:150 dilution of a 2 mM prolume purple solution made up in NanoFuel Solvent (NanoLight Technology) into assay buffer). Then, 5 min after addition of substrate, bioluminescence donor emission (410–480 nm) and BRET acceptor GFP emission (515 nm) were measured on a Pherastar FSX (BMG Labtech, USA) at 37℃ every minute for 5 minutes using a BRET2 optic module. The average of 5 consecutive reads were used to calculate BRET2 ratios and transformed to a baseline-corrected measure of G**α** protein activation using the following equation:

$$G\ protein\ activation\ =\ -1 \times ((BRET2\ ratio_{treatment} - BRET2\ ratio_{DMSO})$$

Where BRET2 ratio is GFP emission signal divided by bioluminescence emission signal, and BRET2 $ratio_{treatment}$ represents the BRET ratio produced by the drug-treated wells and BRET2 $ratio_{DMSO}$ represents the BRET ratio produced by the DMSO-

treated wells (i.e., vehicle control). For Gαq activation, a further normalization step was added to represent data at a percentage between vehicle control (0%) and the signal produced by the 10 µM serotonin plate control (100%).

Ligand Kinetics. Binding buffer consisted of 50 mM Tris HCl, 10 mM $MgCl_2$, 0.1 mM EDTA disodium dihydrate in autoclaved deionized water. The solution was adjusted to pH 7.4 with 10 M $NaOH_{(aq)}$ and stored at 4 °C. This solution was used in membrane preparation generation as well as competition and kinetics assays.

For the generation of membrane preparations, PSYLI2 cells (HEK293T cell line stably expressing psychLight2; mycoplasma free, see *15*) were grown in 15-cm plates with Dulbecco's Modified Eagle Media (DMEM) containing 10% fetal bovine serum and 1% penicillin/streptomycin. When the cells were >90% confluent, the media was removed, and the cells were washed with Dulbecco's Phosphate Buffered Saline (DPBS) (15 mL). Next, DMEM (15 mL) lacking fetal bovine serum and penicillin/streptomycin was added, and the cells were serum-starved for 4–12 hours. Roughly 10 mL of the DMEM was removed, leaving ~5 mL of media in the plates before placing them on ice. Once on ice, the plates were scraped and the contents were transferred to a pre-chilled 50 mL Falcon tube and then centrifuged (2,000 g, 10 min, 4 °C). The supernatant was discarded, and the pellet was resuspended in ice-cold binding buffer (2 mL per 15-cm plate). The cells in the pellet were lysed by 60 s of repeated pipetting with a 10 mL serological pipet on ice, the mixture was then centrifuged (2,000 g, 10 min, 4 °C), the supernatant was discarded, and the pellet was resuspended in ice-cold binding buffer (2 mL per 15-cm plate). The suspension was transferred in 1.0 mL aliquots to pre-chilled Eppendorf tubes, saving a single suspension aliquot of 0.25 mL to use for quantification of protein concentration via a Bradford assay with a Coomassie protein assay reagent. All aliquots were then centrifuged (20,000 g, 20 min, 4 °C). The supernatant was discarded, and the pellets were stored at -80 °C for later use.

Competition binding assays to determine the K_i values of unlabeled compounds, association binding assays to determine k_{on} and k_{off} of [^{3}H]-LSD (PerkinElmer), and kinetics of competition binding experiments to determine k_{off} and k_{on} values of unlabeled compounds were carried out using Millipore MultiScreen HTS FB filter plates that had been pre-soaked with 0.3% $polyethylenimine_{(aq)}$ (PEI) for at least 30 min on a Millipore Multiscreen HTS vacuum manifold. All compounds, radioligands, and membrane preparations were diluted in binding buffer.

For competition binding experiments, [^{3}H]-LSD (2.5X solution in binding buffer, 50 µL, 37.5 nM) and various concentrations of unlabeled compounds (5X solution in binding buffer, 25 µL, 0–50 µM, 0.5% DMSO) were added to the wells. Membrane preparations were thawed on ice before being resuspended in binding buffer. Next, the membrane preparation suspension (50 µL, 0.2 µg/µL, 10 µg) was added, bringing the final volume in each well to 125 µL comprised of [^{3}H]-LSD (15.0 nM), unlabeled compounds (0–10 µM), and a suspension of membrane preparation protein (10 µg). All incubations were carried out in the dark at ambient temperature for 60 min before separation of bound and free radioligand was conducted via rapid vacuum filtration followed by washing with ice-cold binding buffer (3 x 200 µL). Plates were dried and the filter screens were transferred to 7 mL scintillation vials containing 1 mL PerkinElmer Ultima Gold liquid scintillation cocktail and incubated at ambient temperature overnight prior to analysis.

For association binding experiments, total and nonspecific binding was determined by adding binding buffer (2.5X solution, 50 µL, 0.25% DMSO) or mianserin hydrochloride dissolved in binding buffer (250 µM mianserin hydrochloride, 2.5X solution, 50 µL, 0.25% DMSO), respectively, to the wells followed by [^{3}H]-LSD (2.5X solutions, 50 µL, 37.5, 12.5, or 3.75 nM). Membrane preparations were thawed on ice prior to suspension in binding buffer (50 µL, 0.2 µg/µL, 10 µg) and added as the final component in each well at time points spanning 0.25–160 min, bringing the final volume in each well to 125 µL comprised of binding buffer vehicle (0.1% DMSO) or mianserin hydrochloride (100 µM, 0.1% DMSO), [^{3}H]-LSD (15.0, 5.0, or 1.5 nM), and a suspension of membrane preparation protein (10 µg). All incubations were carried out in the dark at ambient temperature before separation of bound and free radioligand was conducted via rapid vacuum filtration followed by washing with ice-cold binding buffer (3 x 200 µL). Plates were dried and the filter screens were transferred to 7 mL scintillation vials containing 1 mL PerkinElmer Ultima Gold liquid scintillation cocktail and incubated at ambient temperature overnight prior to analysis.

For kinetics of competition binding experiments, either unlabeled compounds at concentrations of 0.75*K_i, 2.5*K_i, or 3.25*K_i for their respectively determined K_i values (2.5X solutions, 50 µL, 0.25% DMSO) to determine total binding or mianserin hydrochloride (2.5X solution, 50 µL, 250 µM, 0.25% DMSO) to determine nonspecific binding and [^{3}H]-LSD (2.5X solution, 50 µL, 12.9 nM) were added to the wells. Membrane preparations were thawed on ice prior to suspension in binding buffer (25 µL, 0.4 µg/µL, 10 µg) and added as the final component in each well at time points spanning 0.25–120 min, bringing the final volume in each well to 125 µL comprised of either unlabeled compounds (0.3*K_i, K_i, or 1.3*K_i, 0.1% DMSO) or mianserin hydrochloride (100 µM, 0.1% DMSO), [^{3}H]-LSD (5.16 nM), and a suspension of membrane preparation protein (10 µg). All incubations were carried out in the dark at ambient temperature before separation of bound and free radioligand was conducted via rapid vacuum filtration followed by washing with ice-cold binding buffer (3 x 200 µL). Plates were dried and

the filter screens were transferred to 7 mL scintillation vials containing 1 mL PerkinElmer Ultima Gold liquid scintillation cocktail and incubated at ambient temperature overnight prior to analysis.

Analysis of the radioactivity was conducted using a Beckman LS 6000 liquid scintillation counter. Competition binding data were analyzed in fmol [^{3}H]-LSD/mg protein using the "One site – Fit Ki" nonlinear regression on GraphPad Prism version 9.3.1, constraining HotKdNM to the K_d value of the radioligand determined via saturation binding assays using PSYLI2 membrane preparations (K_d = 5.16 nM) and HotNM to the concentration of radioligand employed in the assays (15.0 nM). To analyze the data using this method, the 0.1% DMSO vehicle condition was set to an arbitrarily dilute concentration of 10^{-25} M to be included on the logarithmic scale of the X-axis. Association binding data were analyzed in specific binding (total binding – nonspecific binding) disintegrations per minute (DPM) using the "Association kinetics - Two or more conc. of hot." nonlinear regression on GraphPad Prism version 9.3.1. Kinetics of competition binding data were analyzed in specific binding (total binding – nonspecific binding) counts per minute (CPM) using the "Kinetics of competitive binding" nonlinear regression on GraphPad Prism version 9.3.1, constraining K1 and K2 to the k_{on} and k_{off} values of the radioligand determined in the association binding assays (1685298 M^{-1} min^{-1} and 0.001282 min^{-1}, respectively) and L to the concentration of the radioligand employed in the assays (5.16 nM).

PsychLight Assays. Psychlight assays were performed using a previously published method (*15*) with slight modifications. Briefly, a solution of PSYLI2 cells suspended in DMEM containing 10% FBS with 5% penicillin-streptomycin was added to poly-D-lysine-coated glass bottom 96-well plates. The cells were plated at a density of 60,000 cells/well for the inner 60 wells and 90,000 cells/well for the outer 36 wells and incubated at 37°C with 5% CO_2 for 18–24 h prior to each experiment. On the day of each experiment, the DMEM solution was replaced with 100 μL/well Hoechst solution (2 μM in HBSS) and incubated for 30 min at 37°C with 5% CO_2. Plates were then washed 3x 200 μL HBSS and replaced with 160 μL/well of assay buffer (HBSS with 0.5 mM HEPES buffer, adjusted to pH = 7.4 with 1 M NaOH) and incubated for 30 min (37°C, 5% CO_2). The cells were then imaged on a Thermo Scientific CellInsight CX7 LZR High Content Analysis Platform at 10X (37°C, 5% CO_2). Images from nine regions of interest (ROI) per well were obtained following the default ROI pattern with no bias to location and no overlap of the ROIs. Baseline images were collected prior to drug addition. Following baseline imaging, cells were treated with 40 μL of drugs at the indicated concentrations (final solutions contained 0.2% DMSO) and incubated (37°C, 5% CO_2) for 1 h before treatment images were acquired using identical parameters. In the case of antagonist mode experiments, cells were co-treated with 10 μM 5-HT and drugs at the indicated concentrations. PsychLight activation was quantified using the built-in *Target Validation Assay (version 6.0.1.4021)* on the Thermo Scientific HCS Studio Cellomics data analysis program. Primary objects were identified and validated using the Hoechst stain and secondary objects (psychLight signal) were defined as signal within 12 pixels of the primary objects. PsychLight signal was calculated as a ΔF/F according to the following equation:

$$\frac{\Delta F}{F} = \frac{\text{psychLight activation (treatment)} - \text{psychLight activation (baseline)}}{\text{psychLight activation (baseline)}}$$

The ΔF/F values were normalized between VEH (0.2% DMSO in assay buffer) (0%) and 10 μM 5-HT (100%).

PsychLight S242A$^{5.46}$ plasmid construction and transfection. To introduce the serine to alanine mutation in psychLight2 at position 5.46 (corresponding to serine 242 at position 5.46 in the wild-type human 5-HT2A receptor), site-directed mutagenesis was performed using the Q5® Site-Directed Mutagenesis Kit according to the manufacturer's protocol (New England Biolabs). In short, primers were designed to incorporate the desired missense mutation during PCR amplification, the resulting linear PCR product was processed using a kinase-ligase-DpnI reaction, and the resulting circularized plasmid was transformed into E. coli. The sequence of the plasmid construct was confirmed using Plasmid-EZ Whole Plasmid Sequencing by Genewiz®.

Primers:

Direction	Sequence	Annealing Temperature
Forward	CAGCTTCGTCgcgTTCTTCATTCCTTTG	57℃
Reverse	CCAATAAGCACGAAATTG	

HEK293T cells were seeded on CellVis glass bottom 96-well plates coated with 50 μg/mL poly-d-lysine at 35,000 cells per well and incubated in 200 μL of DMEM + 10% FBS for 48 h at 37℃ and 5% CO_2. Transfection was carried out using TransIT®-293 transfection reagent according to the manufacturer's protocol (Mirus Bio) and allowed to incubate for 24 h. PsychLight assays were performed as described above.

Comparison Between WT and S242A$^{5.46}$ psychLight Variants. Activity in WT and S242A$^{5.46}$ mutant forms of PsychLight was quantified by deriving ligand activity ratios (log(E_{max}/EC_{50})). First, concentration-response agonist curves for 5-HT, LSD and (+)-JRT were fit to a log(agonist) vs. response (three parameters) curve. The E_{max}/EC_{50} analysis was performed as previously described in detail by others (*16*):

$$Response = Basal + \frac{E_{max} - Basal}{1 + 10^{\log(EC_{50}) - \log[A]}}$$

where E_{max} and Basal represent the top and bottom asymptotes of the curve fit, respectively; EC_{50} represents the concentration that gives a response halfway between E_{max} and Basal; and Log[A] is the logarithm of the agonist concentration. Then, ligand activity ratios, log(E_{max}/EC_{50}) were calculated for each test ligand and statistical comparisons made between each other and between genotypes using a two-way ANOVA and significant interaction effects followed by a Šídák's multiple comparisons test.

ADME and hERG Inhbition Assays. Liver microsome half-life, CYP isozyme inhibition, CYP2D6 utilization, plasma protein binding, hepatocyte clearance half-life, and MDCK-MDR1 assays were conducted at Eurofins. Brain protein binding and hERG inhibition assays were conducted at ChemPartner and Apconix, respectively.

Neuritogenesis Assays. Neuritogenesis Assays were performed as previously described (*17*) with slight modifications. Briefly, 96 well plates were coated with poly-D-lysine and approximately 15,000 cells/well. Neurons were treated after 3 days in vitro (DIV3) for 1 h followed by a complete media change. Cells were incubated for an additional 3 days before fixing and staining for MAP2 on DIV6. Neurites were visualized using a chicken anti-MAP2 antibody (1:10,000; EnCor, CPCA-MAP2) and an anti-chicken IgG secondary antibody conjugated to Alexa Fluor 488 (Life Technologies, 1:500). Plates were imaged in DPBS on a Thermo Scientific CellInsight CX7 High-Content Screening Platform at 20x (N.A. = 0.45). Images were analyzed using the Simple Neurite Tracer and Sholl analysis plug-ins for ImageJ Fiji (version 1.51N). Sholl analysis circle radii = 2 μm increments. All images were taken and analyzed by an experimenter blinded to treatment conditions.

Spinogenesis Assays Using Cultured Rat Cortical Neurons. Spinogenesis experiments were performed as previously described (18). Briefly, 24-well plates with glass coverslips were coated with poly-D-lysine and plated at a density of approximately 35,000 cells/well. Neurons were treated after 18 days in vitro (DIV18) and fixed 24 h after treatment on DIV19 (final concentration of DMSO = 0.1%). For spinogenesis experiments using antagonists, cells were pretreated with ketanserin (10 μM) for 30 min prior to the addition of compounds. For these experiments, the final concentration of DMSO was 0.2% across all wells. Neurites were visualized using a chicken anti-MAP2 antibody (1:10,000; EnCor, CPCA-MAP2) and an anti-chicken IgG secondary antibody conjugated to Alexa Fluor 568 (Life Technologies, 1:500). Spines were visualized with phalloidin conjugated to Alexa Fluor 488 (Life Technologies, 1:40). The images were taken on a Nikon High Content Spinning Disk Confocal microscope with a 100x oil objective (N.A. = 1.45). Images were analyzed using ImageJ Fiji (version 1.51N). All images were taken and analyzed by an experimenter blinded to treatment conditions.

In Vivo Spinogenesis and Synaptogenesis. Materials were sourced from Electron Microscopy Sciences (Hatfield, PA) unless noted otherwise. Postnatal day 62 female C57BL/6J mice (Jackson Laboratory, Sacramento, C.A.) were treated with VEH (saline) or (+)-JRT (n = 3/group). After 24 h, the animals were sacrificed via transcardial perfusion with warmed (37°C) oxygenated Ringer's solution followed by warmed fixative (2% paraformaldehyde, 2.5% glutaraldehyde, 3 mM calcium chloride in 0.1 M cacodylate buffer). Brains were carefully removed from the skull and post-fixed in fresh fixative at 4°C overnight. Brains were then rinsed with PBS and 100 μm coronal sections spanning the prefrontal cortex were collected using a vibrating microtome (Leica VT1000). Sections were incubated in a cryoprotectant (30% glycerol, 30% ethylene glycol, in 1× PBS) overnight at 4°C before storage at -20°C until further processing (*19*).

Subregions spanning from the medial cortical surface to the corpus collosum of the infralimbic cortex were microdissected according to the Allen brain atlas (http://mouse.brain-map.org/) and processed further for electron microscopy as described elsewhere (*20,21*) with some modifications. Briefly, samples were stained with buffered 1.5% reduced osmium tetroxide for 45 min, rinsed thoroughly, further stained with 1% aqueous uranyl acetate overnight at 4°C, serially dehydrated in ethanol and embedded in Eponate 12 epoxy resin (hard formulation, Ted Pella, #18012). A blockface that spanned from the medial cortical surface to the corpus collosum with a height of approximately 150 μm was produced using a 90° diamond trim tool (Diatome), and ribbons of 130–270 serial ultrathin serial sections of 55 nm thickness were collected onto 30 × 6 mm silicon chips partially submerged in a Diatome Histo diamond knife (Diatome) on a Leica UC7 ultramicrotome. Serial sections on silicon chips were labeled with diamond scribes and mounted onto aluminum stubs with carbon tape before they were loaded into a Zeiss Sigma VP scanning electron microscope (SEM) for imaging using a backscattered electron detector (Gatan) and ATLAS5 control software (FIBICS) for mapping and imaging of serial sections (*21,22*).

Briefly, for each dataset the ribbon of serial sections was located in the SEM, and low- (500 nm/px) and medium- (80 nm/px) resolution maps were made of the ribbon and each section, respectively. The apical region of cortical layer 1 was identified as the acellular region spanning from the interface with the meninges to roughly 200 µm below the cortical surface, with most dendrites in this region having a diameter of less than 2 µm. A region of interest (ROI) composed mostly of neuropil (e.g. minimal vasculature, cell bodies, etc.) was identified through the medium-resolution series. The ROI was then imaged at high resolution on each section: images from consecutive 55 nm sections were collected with a lateral pixel size of 8 nm, an accelerating voltage of 3kV in high current mode, using a 30 µm aperture, and a working distance of 6 mm. Image stacks were collated and rigidly aligned using TrakEM2 in Fiji (*23*) and cropped to a minimum contiguous volume of aligned data with minimal padding. Fine image stack alignment was accomplished using SWiFT-IR (*24*) as deployed on 3DEM.org using the TACC compute resource Stampede 2 (*25*). The continuous 3DEM datasets for each animal constituted volumes of at least 1000 μm^3 in dimension.

For spinogenesis analysis, spiny dendrites found in cross-section in a central XY-plane of each dataset were identified (N = 8 per animal). The centerline of each dendrite and those of each of their contiguous spines were manually segmented from the dendritic interface to the post synaptic density using VAST Lite (*26*). The lengths of traced dendritic segments and the number of spines per dendrite were counted, providing a measure of spine density along the dendritic segment. Representative skeletons were exported from VAST Lite as .obj files and visualized using Blender software (version 2.79; *27*).

For synapse density analysis, non-overlapping sub-volumes of 3 × 3 × 3 µm (27 μm^3; N = 6 per S3EM dataset) were randomly sampled from the S3EM datasets. Sub-volumes were visually inspected and volumes containing prominent occurrences of vasculature, neuronal somas and artifacts were excluded to ensure subsequent analyses were limited to dense neuropil regions. All postsynaptic densities (PSDs) in the sampled sub-volumes were identified and manually segmented in VAST Lite. As described elsewhere (*28*), PSDs were defined as regions of post-synaptic membrane with darker staining, directly apposed to a cluster of presynaptic vesicles. Synapse count per μm^3 and synapse volumes were calculated from the annotated volumes.

In Vivo Spinogenesis after chronic corticosterone administration. Male and female Thy1-EGFP mice were given 1 week to acclimate to the new vivarium, then transferred to new cages (individually housed). Once individually housed, the mice were administered corticosterone (20 mg/kg; IP) in DMSO (2 mL/kg) or gently handled daily for 20 days. On day 21, mice were treated with VEH (saline) or (+)-JRT (1 mg/kg; IP). After 24 h, mice were anesthetized with isoflurane and transcardially perfused with paraformaldehyde (PFA, 4% in PBS, 7.4 pH). After extraction of the brains, samples were fixed in 4% PFA at 4°C overnight. The brains were cryo-protected by immersion in a 15% sucrose solution (15% sucrose in PBS w/v) overnight. Next, samples were placed in 30% sucrose solution (30% sucrose in PBS w/v) for at least 1 day. Samples were then embedded in optimal cutting temperature compound (OCT), sliced into 100 µm coronal sections using a cryostat, and mounted on slides which were left in the dark overnight to dry. Coverslips were mounted onto microscope slides using ProLong Gold. Slides were imaged on a Nikon HCA Spinning Disc confocal microscope equipped with a 100x/NA 1.45 oil objective. Images of dendrites and dendritic spines within layer 2/3 of the mPFC were captured and manually counted by a trained experimenter blinded to treatment conditions.

Head-Twitch Response Assay. The HTR assay was performed using equal numbers of male and female C57BL/6J mice. The mice were obtained from The Jackson Laboratory (Sacramento, CA) and were approximately 8 – 12 weeks old at the time of the experiments. After compound administration, animals were placed into an empty arena (40 cm x 40 cm) and filmed for 20 min. The arena was cleaned with 70% ethanol between trials. Animals were given a one-week washout period before being tested again. Animals were tested a maximum of 4 times. All drug treatments were randomized and no animal received the same drug and dose twice (*29*). For the blocking experiments, animals were administered (+)-JRT (1 mg/kg) or vehicle (saline) via IP injection and placed in an empty cage for 15 min. Animals were then administered LSD (0.2 mg/kg, IP), placed in the test arena, and filmed for 20 min. Videos were scored later by two blinded observers, and the results were averaged (Pearson correlation coefficient > 0.9).

Head-Twitch Antagonism of an LSD Dose Response. The HTR assay was performed by Transpharmation using male C57BL/6J mice (8 – 9 weeks old). Animals were habituated to a test cage (standard cage used to house mice) for 20 min prior to the test. Next, mice were administered vehicle (1% DMSO) or LSD at various doses (IP, 10 mL/kg), placed immediately in the test cage, and observed for 20 min to quantify head-twitch response. For blocking studies, 0.9% saline (vehicle) or (+)-JRT (1 mg/kg, IP, 10 mL/kg) was administered 30 min prior to LSD or 1% DMSO.

Novelty-Induced Locomotion. The novelty-induced locomotion assay was performed using male and female C57BL/6J mice (23–33 weeks old) bred in-house (University of California, Davis). Animals were administered (+)-JRT (1 mg/kg) or 0.9% saline VEH (5 mL/kg, i.p) and were placed in a novel test arena (40 cm x 40 cm) for 30 min. Locomotion during the experiment was quantified using ANYmaze Video Tracking System, version 7.30 (Stoelting Co.). The test arena was cleaned thoroughly with 70% ethanol between trials.

Amphetamine-Induced Locomotion. The amphetamine-induced hyperlocomotion assay was performed using male and female C57BL/6J mice that were approximately 8 weeks old at the time of the experiments. Animals were first placed in the test arena (40 cm x 40 cm) for 15 min to obtain a basal reading of locomotion and habituate them to the testing arena. Next, animals were administered (+)-JRT (1 mg/kg) or VEH (2.5 mL/kg, i.p) and placed back in the test arena. After 15 min, the animals were administered (+)-amphetamine (3 mg/kg) or VEH (2.5 mL/kg) and placed back in the test arena for 60 minutes. Locomotion during the entire experiment was quantified using ANYmaze Video Tracking System, version 7.07 (Stoelting Co.). The test arena was cleaned thoroughly with 70% ethanol between trials.

PCP-Induced Locomotion. The PCP-induced hyperlocomotion assay was performed by Psychogenics using male C57BL/6J mice that were approximately 8 weeks old at the time of the experiments. The experiment was performed during the animal's light cycle phase. On the day of testing, animals were acclimated to the experimental room for at least 1 h prior to treatment. Animals were then administered with VEH or JRT (IP, 10 mL/kg) and placed in holding cages for 30 min, after which mice were placed in the open field chambers (27.3 x 27.3 x 30.3; Med Associates Inc., St Albans, VT). Baseline activity was recorded for 30 min. Next, mice received either PCP (5 mg/kg, IP, 10 ml/kg) or VEH and were returned to the open field chambers for a 60 min test session. Locomotion was assessed using consecutive infrared beam breaks. At the end of each session, the chambers were thoroughly cleaned.

Prepulse Inhibition. Male and female C57BL/6J mice (9–10 weeks old) were obtained from the Jackson Laboratory (Sacramento, CA, USA) and housed in a temperature- and humidity-controlled vivarium at the University of California, Davis. Mice were allowed to habituate to the vivarium for at least one week upon arrival prior to drug administration and assessment of prepulse inhibition (PPI). Animals received a pretreatment of (+)-JRT (1 mg/kg, IP, 5 mL/kg) or vehicle (0.9% saline, IP, 5 mL/kg) and were individually housed in a holding cage. After 15 min the animals were treated with vehicle or MK-801 (3 mg/kg, IP, 5 mL/kg) and were immediately placed into the PPI chamber to initiate the first phase of the PPI assessment. The experiment started with 6 min of no sound to habituate the animals to the chambers and assess baseline movement. Next, startle response was measured in response to five 40 ms sound bursts of 80, 90, 100, 110, or 120 dB (random order, fixed intertrial interval). Treatment did not impact startle response. To measure pre-pulse inhibition of the acoustic startle, mice were presented with each of seven trial types across six discrete blocks of trials for a total of 42 trials, over 10.5 min (fixed intertrial interval). Two of the trial types were no stimulus and a 110 dB sound burst lasting for 40 ms, similar to block 1, to measure minimum and maximum startle responses within the block. The remaining five trial types included a 40 ms, 110 dB sound burst presented 100 ms following a 20 ms pre-pulse acoustic stimulus of varying intensities: 74, 78, 82, 86, and 92 dB. The trial types were presented in pseudorandom order such that each trial type was presented once within a trial block. Percent PPI was calculated according to the formula: 100 – [(pre-pulse + pulse)/pulse alone) × 100.

Transcriptomics. After three days of handling, LSD (1 mg/kg, IP), (+)-JRT (1 mg/kg, IP), or vehicle (saline) were administered to 36 C57BL/6 mice (n = 36 total = 6 per sex per treatment) ensuring cage matching. Twenty-four hours after treatment, animals were sacrificed, and the prefrontal cortex was dissected. Next, RNA was extracted from one hemisphere per animal (hemisphere laterality was randomly selected but we ensured equal representation across groups). Total RNA was isolated with Invitrogen RNAqueous Total RNA Isolation Kit (AM1912) and submitted to Novogene for library preparation and sequencing following polyA enrichment to a target of 35 million 150 bp paired end reads per sample, with all samples passing both their external and our internal quality control pipelines. Reads were aligned to the GRCm39 genome assembly using STAR, processed with Picard, and counted with the Subread software. Transcript inclusion criteria consisted of a minimum raw expression level of 25 transcripts in a minimum of 4 samples. Surrogate variable analysis was conducted with the sva package 2023 release v3.5.0 in R and normalization and differential expression analysis was performed with DESeq2 v4.3 with a baseMean threshold of 10. Heatmaps were created with the pheatmap v1.0.12 with sample-wise z-scores plotted and both row and column grouping allowed using pearson's distances and ward.D for clustering. Gene-wise selection criteria for heatmaps were a baseMean expression threshold of 50 to focus on robustly expressed genes, p value < 0.05, and presence in the top 250 genes sorted by absolute value of log2FC. Additionally, z score was capped at 2 for visualization purposes. Schizophrenia gene sets were extracted from a recent TWAS study (*30*). For Figure S8G, the TWAS set was filtered for FDR < 0.01. Permutation testing was conducted to quantify the overlap of top genes from schizophrenia TWAS with the perm_test function from the bulkWarehouse package 2023 release v1.2.7. Briefly, the intersection of top differentially expressed genes and top TWAS genes is compared to an empirical distribution created by calculating the intersection with our top DEGs when randomly sampling candidate genes from our dataset equal to the size of the TWAS sample. Selection criteria from the TWAS include an expression level of 25 transcripts in a minimum of 4 samples in our dataset, FDR < 0.01 from the TWAS and top 250 sorted by absolute value of log2FC. Selection criteria from our gene set were an expression level of 25 transcripts in a minimum of 4 samples, p value < 0.1 and top 250 sorted by absolute value of log2FC.

Rat Forced Swim Test (FST). This study was performed by Psychogenics (Paramus, NJ). Male Sprague Dawley rats were purchased from Envigo (Indianapolis, IN), and were 8 weeks old at the time of the experiments. Upon receipt, they were assigned unique identification numbers (tail marked) and group housed in ventilated cages with 3 rats per cage. All animals

remained housed in groups of three for the remainder of the study. All rats were acclimated to the colony room for up to one week prior to dosing. During the period of acclimation, rats were examined and handled daily, and weighed to assure adequate health and suitability. The room temperature was maintained between 20ºC and 23ºC with a relative humidity of 30%–70%. Lab Rodent Diet 5001 (W.F. Fisher, Cat # 11015) and water were provided ad libitum. Animals were randomly assigned across treatment groups. All testing was performed during the light phase of the light/dark cycle. Behavioral testing was conducted according to established protocols approved by the IACUC committee and PGI Standard Operation Procedures (SOP). Each forced swim chamber was constructed of clear acrylic (height = 40 cm; diameter = 20.3 cm). Only one rat was placed in the swim chamber at a time for each swim test. The water was changed and the chamber cleaned between each animal. The water depth was 16 cm in the first swim session (pre-test) and 30 cm in the second swim session (test). The water was maintained at 23ºC ± 1ºC for all swim sessions. At the end of each swim test rats were dried with paper towels and returned to the home cage. All animals were carefully monitored to ensure their safety in the swim test and any animal unable to maintain a posture with its nose above water was immediately removed from the water and not used further in the study. Animals were first subjected to a pre-test for 15 min. Immediately after the pre-test, they were administered compounds or VEH via i.p. injection (1 ml/kg). Racemic ketamine hydrochloride (10 mg/kg) was used as a positive control. A salt correction factor of 1.36 was used when preparing formulations of (+)-JRT to ensure that the dose corresponded to that of the free base. A second FST was conducted 24 h after compound administration. This test lasted for 5 min and was video recorded. A blinded experimenter manually scored the videos for swimming, climbing, and immobility behavior. Scoring of the forced swim test was performed by trained technicians using a time sampling technique in which the animal in the video recorded test was viewed every 5 seconds and the behavior observed was noted (e.g., immobile, swimming, or climbing). A total of 60 behaviors were noted per subject per session.

Sucrose Preference Test (SPT). Male and female C57BL/6J mice (9–10 weeks old) were obtained from the Jackson Laboratory (Sacramento, CA, USA) and housed in a temperature- and humidity-controlled vivarium at the University of California, Davis. Mice were given ad libitum access to food and water and were maintained on a 12 h light/dark cycle. Animals were allowed to habituate to the vivarium for at least one week upon arrival prior to chronic corticosterone (CORT, Spectrum Chemical, Gardena, CA, USA) administration and were then individually housed to establish baseline responsiveness to sucrose prior to CORT administration. Mice remained individually housed until the experiment was completed. On Day 0, animals were given access to sucrose (2%) and water so that preference could be assessed 24 h later on Day 1. Sucrose preference was calculated as the amount of sucrose consumed divided by the total amount of liquid consumed during a 24 h period. A score of 0.5 indicates no preference for sucrose, above 0.5 indicates a preference for sucrose, and below 0.5 indicates a preference for water. Fresh solutions of sucrose were prepared for each drinking session. Animals displaying a baseline preference for sucrose prior were considered responders (10/15 male and 10/15 female) and subsequently received daily administration of CORT (20 mg/kg, IP, 2 mL/kg) dissolved in USP-grade DMSO (Spectrum Chemical, Gardena, CA, USA) on Days 1–10. On Day 11, CORT was not administered and sucrose preference was assessed on Day 12. No significant change in sucrose preference was observed on day 12, so the animals received an additional 10 days of CORT administration on Days 12–21. On Day 23, sucrose preference was assessed and reductions below 0.5 were classified as CORT-sensitive (5/10 males and 4/10 females). On Day 23, mice that were CORT-sensitive were administered (+)-JRT (1 mg/kg, IP, 3 males and 2 females) or VEH (2 male and 2 female)and sucrose preference was assessed on Day 25. The animals received a second dose on Day 25 and sucrose preference was assessed again on Day 27 and Day 35.

Probabilistic Reward Task (PRT). Twenty-four adult male Long Evans rats obtained from Charles River Laboratories (Wilmington, MA) weighing between 250 and 300 g were used in the study. Subjects were maintained at approximately 80% of their free-feeding weight via post-session portions of rodent chow and had unrestricted access to water in their home cage. Schematics of the rodent touch-sensitive experimental chamber have been reported previously (*31*). All experimental events and data collection were programmed in E-Prime Professional 2.0 (Psychology Software Tools, Inc., Sharpsburg, PA). Subjects were trained to engage with the touchscreen and discriminate long versus short lines using previously published protocols (*32*). Trials began with presentation of a long (30 x 7 cm) or short (15 x 7 cm) white line presented 5 cm above two 5 x 5 cm blue response boxes left and right of center in a quasi-random fashion across 100-trial sessions (50 trials of each length). Subjects learned to respond to the left or right response box depending on the length of the white line to earn 0.1 mL of 30% sweetened condensed milk, which was paired with an 880 ms yellow screen flash and 440 Hz tone and followed by a 5 s blackout period. Training continued until accuracies for both line lengths were >75% correct for 3 consecutive sessions. Next, probabilistic reinforcement schedules were introduced based on the human PRT protocol (*33*); that is, a 3:1 rich:lean probabilistic schedule such that 60% of correct responses to one of the line lengths and 20% of correct responses to the other line length were rewarded. These probabilistic contingencies were assessed across 2 consecutive sessions to define baseline control values prior to initiation of drug testing. Next, subjects were exposed to a chronic inescapable ice-water stress protocol by placing subjects daily in a tank of water, iced to 10°C, which the average adult male rat is able to swim in for approximately 6-10 min before sinking. Immediately following submersion, the subject is rescued from the cold-water tank. Two and one-half h later, PRT testing begins. This protocol continued daily until a blunting in log *b* values was observed (usually following ~5-7 days). Next, subjects received acute administrations of either vehicle (saline), 10 mg/kg ketamine, or 1 mg/kg (+)-JRT 30 min following cold-water rescue to avoid drug treatment under

hypothermic states. Next, 2 h following drug treatment, PRT testing commenced to examine each drug treatment's ability to restore responsivity to reward. Chronic stress continued for 7 d post-drug treatment and also included PRT sessions at 24 h, 3 d, and 7 d post-drug treatment to document time course of drug effects observed on PRT outcomes. The PRT yields two signal detection metrics (*34*), response bias (log *b*) and task discriminability (log *d*), that are quantified by examining the number of *Correct* and *Incorrect* responses in *Rich* and *Lean* trial types as follows:

$$\log b = \frac{1}{2}\log\left(\frac{(\text{Rich}_{\text{Correct}} + 0.5) * (\text{Lean}_{\text{Incorrect}} + 0.5)}{(\text{Rich}_{\text{Incorrect}} + 0.5) * (\text{Lean}_{\text{Correct}} + 0.5)}\right)$$

$$\log d = \frac{1}{2}\log\left(\frac{(\text{Rich}_{\text{Correct}} + 0.5) * (\text{Lean}_{\text{Correct}} + 0.5)}{(\text{Rich}_{\text{Incorrect}} + 0.5) * (\text{Lean}_{\text{Incorrect}} + 0.5)}\right)$$

All data (log *b*, log *d*, rich reaction time, lean reaction time) were subject to repeated-measures 2-way analysis of variance (ANOVA).

Pharmacokinetics Studies. Pharmacokinetics studies were performed at Shanghai ChemPartner Co., Ltd (Shanghai, China). Male Sprague Dawley rats (190 – 206 g) were purchased from Shanghai JiHui Laboratory Animal Co., Ltd (Shaghai, China) and were administered (+)-JRT fumarate via IP injection (1 mg/kg, 5 mL/kg) after overnight fasting. Animals were sacrificed and plasma and brain samples were collected at time points of 0.25, 0.5, 1, 2, and 4 hours post dose (N = 3 per time point). Approximately 150 µL of blood was collected into a K2EDTA tube via jugular vein. Blood samples were put on wet ice and centrifuged to obtain plasma samples (2000 g, 5 min, 4 °C) within 15 min. After blood collection, a mid-line incision was made into the scalp and the skin retracted. The skull overlaying the brain was removed, and the whole brain was collected, rinsed with cold saline, dried on filter paper, weighed, and snap frozen via dry ice. Plasma and brain samples were stored at approx. -70 °C until analysis.

For plasma samples, an aliquot of 30 µL sample was mixed with 200 µL internal standard (IS) (propranolol, 40 ng/mL in MeCN). The mixture was vortexed for 1 min and centrifuged at 5800 rpm for 10 min. The supernatant was collected and 0.5 µL was injected for LC-MS/MS analysis. Brain samples were homogenized with 2 volumes (v/w) of PBS (dilution factor of 3). The procedure for diluted brain samples was the same as the plasma samples. Plasma concentrations are reported as ng/mL and brain concentrations, with a dilution factor of 3 applied, are reported as ng/g. Standards of (+)-JRT were prepared as working solutions in 50:50 (v:v) MeCN:H_2O, and 3 µL of working solution was mixed with 57 µL of blank rat plasma or blank rat brain homogenate at a concentration range of 1.00 – 3000 ng/mL (plasma) or ng/g (brain homogenate). The procedure for the standards was the same as the plasma samples, and the respective calibration curves were linear.

LC-MS/MS analysis was performed using a SCIEX Triple Quad 6500+. Separation was performed on a Waters ACQUITY UPLC BEH C18 (2.1 x 50 mm, 1.7 µm) column. Mobile phases of H_2O with 0.025% formic acid and 1 mM NH_4OAc (A) and MeOH with 0.025% formic acid and 1mM NH_4OAc (B) were pumped at a flow rate of 0.60 mL/min and column temperature of 60 °C. The gradient system was as follows: 100:0 A/B (0 – 0.15 min), 95:5 A/B (0.15 – 0.90 min), 50:50 A/B (0.90 – 1.50 min), 30:70 A/B (1.50 – 1.51 min), 5:95 (1.51 – 2.01 min), 95:5 (2.01 – 2.50 min); the retention time of (+)-JRT and IS were 1.61 min and 1.43 min respectively, and the analytes were ionized under a positive ion spray mode and detected through multiple-reaction-monitoring (MRM) of mass transition pairs at m/z 324.40→223.00 for (+)-JRT and 260.10→116.20 for IS.

4-Odor Discrimination and Reversal. A 4-odor discrimination and reversal behavioral assay was performed as described previously (*35,36*) with slight modifications. Food restriction was begun five days before the discrimination and reversal tests to reduce animals' body weight to ~80% of their starting weight. A total of 31 C57BL/6J mice 8–12 weeks of age were used in this assay. Eleven mice (5 males, 6 females) served as VEH/unstressed controls, 10 animals (4 males, 6 females) as a VEH/stress group, and 10 animals (4 males, 6 females) as a treatment/stress group. Both the VEH/stress and treatment/stress groups were subjected to a 7-day unpredictable mild stress protocol using previously validated stressors (*35,37,38*) Two stressors were delivered during the day, with additional overnight stressors on 4 of the days. In brief, the following stressors were used: Day 1: AM = 30 min of predator order, PM = instability (wet bedding + tilt for 30 min), Overnight = tilted cage; Day 2: AM = overcrowding/social interaction (30 min), PM = restraint stress (30 min), Overnight = none; Day 3: AM = restraint stress (30 min), PM = predator odor (30 min), Overnight = none; Day 4: AM = exposure to a new room (30 min) + tail suspension (6 min), PM = restraint stress (30 min), Overnight = tilted cage; Day 5: AM = instability (wet bedding + tilt for 30 min), PM = overcrowding/social interaction (30 min), Overnight = none; Day 6: AM = white noise/room change, PM = instability (wet bedding + tilt for 30 min), Overnight = tilted cage; Day 7: AM = restraint stress (30 min), PM = overcrowding/social interaction (30 min), Overnight = light exposure.

Following unpredictable mild stress, the animals underwent habituation/shaping/training on Days 7 and 8 before discrimination and reversal testing on day 9. The behavioral apparatus was a 12" x 12" x 9" (length x width x height) opaque white acrylic box with 3" long transparent acrylic internal walls in the center of each exterior wall to create 4 quadrants and

a removable transparent cylinder of diameter 6” that fit in the center of the box. On the first training day (Day 7), mice were habituated to the apparatus and four 4-oz white ceramic pots (Yachi, www.amazon.com), which were each placed in a corner of one of the box’s quadrants with a piece (~0.015 g) of Honey Nut Cheerio (General Mills, Golden Valley, MN) as food reward inside. For this first day of habituation, mice were placed in the center cylinder, and the cylinder was then removed to begin each round of habituation; mice were allowed to explore freely until all 4 pots’ food rewards were consumed or 10 minutes had passed. Mice were then returned to the cylinder and pots were rebaited as necessary, for a total of 6 rounds of habituation during day 1.

On the second training day (Day 8), VEH (saline) or (+)-JRT (1 mg/kg) was administered via IP injection. After a short recovery period, shaping was performed with one pot. The food reward was delivered with increasing amounts of pine shavings (Living World, www.amazon.com) covering it which required the mouse to dig to obtain the reward. The pot was moved between the four apparatus quadrants so that each position was rewarded equally; after finding and consuming the food reward, the mouse was returned to the center cylinder between trials. Trials began with the food reward placed in an empty dish (4 trials), then with a dusting of pine bedding added (4 trials), followed by trials with the dish a quarter full (4 trials), half full (4 trials) and full (12 trials) with pine shavings.

On testing day (Day 9), four pots were filled with shavings and had a piece of filter paper scented with a drop of essential oil (LorAnn Oils, Lansing, MI) attached to the inner rim. Rosemary, thyme, clove, and nutmeg were used in the initial discrimination phase, with rosemary serving as the rewarded odor which indicated which pot contained the food reward. The mouse was placed in the center cylinder and then allowed to explore the arena after the cylinder was removed at the start of each trial. Trials ended after either the food reward was located and eaten, digging was initiated in an unrewarded pot, or three minutes passed without digging occurring. The mouse was then returned to the center cylinder, the rewarded pot was rebaited if necessary, and pots were repositioned so that no one pot remained in the same quadrant during consecutive trials. A trial in which no digging was observed was recorded as an omission; after two consecutive omissions, a pot was placed in the center cylinder with pine shavings and a food reward was placed in a well within the shavings, in order to potentiate digging. Mice passed this discrimination phase when they successfully located and ate the food reward in 8 of 10 consecutive trials. Immediately following discrimination, pine shavings were replaced in all pots and the thyme odorant was replaced with a novel cinnamon odorant. The rewarded odorant was changed from rosemary to clove. Mice were again considered to have passed this reversal phase after successfully locating and eating the food reward in 8 of 10 consecutive trials.

General Information for Chemical Synthesis

All reagents were obtained from commercial sources and reactions were performed using oven-dried glassware (120 °C) under an inert N_2 atmosphere unless otherwise noted. Air- and moisture-sensitive liquids and solutions were transferred via syringe or stainless-steel cannula. Organic solutions were concentrated under reduced pressure (~5 Torr) by rotary evaporation. Solvents were purified by passage under 12 psi N_2 through activated alumina columns. Chromatography was performed using Fisher Chemical™ Silica Gel Sorbent (230–400 Mesh, Grade 60). Compounds purified by chromatography were typically applied to the adsorbent bed using the indicated solvent conditions with a minimum amount of added dichloromethane as needed for solubility. Thin layer chromatography (TLC) was performed on Merck silica gel 60 F254 plates (250 µm). Visualization of the developed chromatogram was accomplished by fluorescence quenching or by staining with aqueous potassium permanganate or Ehrlich's reagent.

Nuclear magnetic resonance (NMR) spectra were acquired on a Bruker 400 operating at 400 and 100 MHz for ^{1}H and ^{13}C, respectively, and are referenced internally according to residual solvent signals. Data for ^{1}H NMR are recorded as follows: chemical shift (δ, ppm), multiplicity (s, singlet; d, doublet; t, triplet; q, quartet; quint, quintet; m, multiplet), coupling constant (Hz), and integration. Data for ^{13}C NMR are reported in terms of chemical shift (δ, ppm). Infrared spectra were recorded using a Thermo Nicolet iS10 Fourier transform infrared (FT-IR) spectrometer with a Smart iTX Accessory [diamond attenuated total reflection (ATR)] and are reported in the frequency of absorption (ν, cm^{-1}). Liquid chromatography-mass spectrometry (LC-MS) was performed using a Waters LC-MS with an ACQUITY Arc QDa detector. Specific rotation measurements were performed on an AUTOPOL IV Automatic Digital Polarimeter.

Detailed Synthetic Procedures and Experimental Data for All Compounds

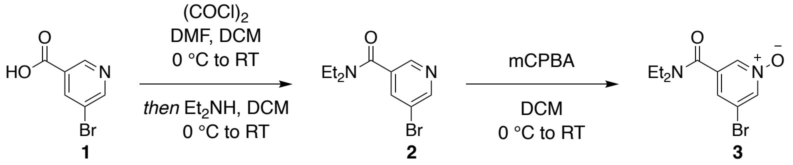

3-bromo-5-(diethylcarbamoyl)pyridine 1-oxide (3)

To a 0°C cooled mixture of 5-bromonicotinic acid (10.000 g, 49.503 mmol, 1.0 equiv) in DCM (250 mL) was added oxalyl chloride (6.37 mL, 74.2 mmol, 1.5 equiv) slowly. Then, DMF (0.5 mL) was added to the suspension dropwise. The mixture was warmed to ambient temperature and stirred for 1 h. The mixture was cooled to 0 °C and a solution of diethylamine (25.61 mL, 247.5 mmol, 5.0 equiv) in DCM (250 mL) was added slowly via cannula. The mixture was warmed to ambient temperature and stirred for 30 min. Next, H_2O (500 mL) was added followed by 2M HCl (40 mL) until the pH = 1–2. The layers were separated, and the aqueous layer was further extracted with DCM (3 x 200 mL). The organic extracts were combined and washed sequentially with saturated aqueous $NaHCO_3$ (1 x 250 mL) and brine (1 x 250 mL), dried over Na_2SO_4, and concentrated under reduced pressure.

To a 0°C cooled solution of the resulting brown oil in DCM (200 mL) was added mCPBA (70–75% balance) (22.781 g, 99.006 mmol, 2.0 equiv). The mixture was warmed to ambient temperature and stirred for 18 h. To the solution was added saturated aqueous $NaHCO_3$ (500 mL) and then 1M NaOH (500 mL). The layers were separated, and the aqueous layer was further extracted with 10% isopropanol in DCM (3 x 200 mL). The organic layers were combined, dried over Na_2SO_4, filtered, and concentrated under reduced pressure. The residue was purified via chromatography on silica gel (EtOAc then 12% MeOH in EtOAc) and concentrated under reduced pressure. The resulting pale-yellow oil was dissolved in DCM (50 mL), and hexanes (500 mL) were slowly added to the solution with vigorous stirring. The resulting suspension was cooled to 0 °C, filtered, and washed with 100 mL ice-cold hexanes to afford **3** (11.041 g, 82%) as a white solid.

^{1}H NMR (400 MHz, $CDCl_3$) δ = 8.32 (t, *J* = 1.5 Hz, 1H), 8.09 (t, *J* = 1.3 Hz, 1H), 7.35 (t, *J* = 1.3 Hz, 1H), 3.54 – 3.46 (m, 2H), 3.29 – 3.21 (m, 2H), 1.24 – 1.12 (m, 6H) ppm.
^{13}C NMR (100 MHz, $CDCl_3$) δ = 164.3, 141.0, 136.3, 135.9, 126.4, 120.7, 43.5, 39.9, 14.4, 12.8 ppm.
LRMS (ES$^+$) *m/z* [M + H]$^+$ calcd for $C_{10}H_{14}BrN_2O_2^+$ 273.02; Found 273.12.
IR (diamond, ATR) ν 3445, 3068, 2973, 2934, 1633 cm^{-1}.

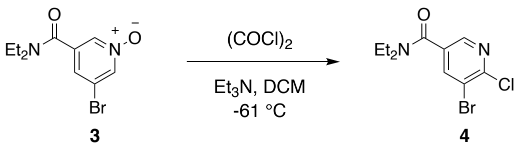

5-bromo-6-chloro-*N*,*N*-diethylnicotinamide (4)

A solution of **3** (9.900 g, 36.395 mmol, 1.0 equiv) and Et_3N (10.15 mL, 72.79 mmol, 2.0 equiv) in DCM (180 mL) was cooled to -61 °C ($CHCl_3$/dry ice) prior to the dropwise addition of oxalyl chloride (6.24 mL, 72.8 mmol, 2.0 equiv). The mixture was stirred for 30 min, then MeOH (5 mL) was added slowly before warming the solution to ambient temperature and adding saturated aqueous $NaHCO_3$ (25 mL). The solution was poured into 1M NaOH (600 mL) and the layers were separated. The aqueous layer was further extracted with DCM (3 x 150 mL). The organic extracts were combined, washed with brine (250 mL), dried over Na_2SO_4, and concentrated under reduced pressure. The residue was purified by chromatography on silica gel (25% EtOAc in hexanes) to afford **4** (9.442 g, 89%) as a crystalline white solid.

^{1}H NMR (400 MHz, $CDCl_3$) δ = 8.34 (d, *J* = 2.04 Hz, 1H), 7.96 (d, *J* = 2.04 Hz, 1H), 3.59 – 3.42 (m, 2H), 3.36 – 3.18 (m, 2H), 1.28 – 1.10 (m, 6H) ppm.
^{13}C NMR (100 MHz, $CDCl_3$) δ = 166.0, 151.5, 145.3, 140.7, 133.0, 120.5, 43.6, 39.9, 14.4, 12.9 ppm.
LRMS (ES$^+$) *m/z* $[M + H]^+$ calcd for $C_{10}H_{13}BrClN_2O^+$ 290.99; Found 291.00.
IR (diamond, ATR) ν 2974, 2935, 1627, 1574 cm^{-1}.

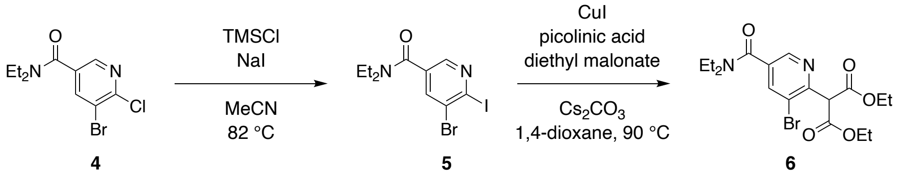

diethyl 2-(3-bromo-5-(diethylcarbamoyl)pyridin-2-yl)malonate (6)

To a vigorously stirred mixture of **4** (5.000 g, 17.243 mmol, 1.0 equiv) and NaI (20.676 g, 137.94 mmol, 8.0 equiv) in acetonitrile (40 mL) was added TMSCl (3.28 mL, 25.86 mmol, 1.5 equiv) slowly. The mixture was stirred at ambient temperature for 30 min, then heated at reflux for 1 h, with ¼ of the reaction volume removed and collected in a Dean-Stark receiver during this time period. The resulting yellow suspension was cooled to ambient temperature, diluted with DCM (150 mL), and added to a saturated aqueous solution of $NaHCO_3$ (250 mL). With vigorous stirring, a saturated aqueous solution of $Na_2S_2O_3$ (100 mL) was added, followed by 1M NaOH (80 mL). The resulting clear solution was transferred to a separatory funnel and the layers were separated. The aqueous layer was further extracted with DCM (3 x 100 mL). The organic extracts were combined, washed with brine (200 mL), dried over Na_2SO_4, and concentrated under reduced pressure.

The resulting pale orange solid was added to a sealable screw cap flask along with copper(I) iodide (0.164 g, 0.861 mmol. 0.05 equiv), picolinic acid (0.212 g, 1.72 mmol, 0.1 equiv), and Cs_2CO_3 (16.854 g, 51.729 mmol, 3.0 equiv). Next, 1,4-dioxane (43 mL) and diethyl malonate (5.26 mL, 34.5 mmol, 2.0 equiv) were added and the flask was capped. The mixture was heated at 90 °C and stirred for 16 h. The mixture was cooled to ambient temperature and filtered over celite. The filter cake was washed with EtOAc (200 mL). The filtrate was added to H_2O (500 mL), then 1M HCl (10 mL) was added, and the layers were separated. The aqueous layer was further extracted with EtOAc (2 x 200 mL). The organic extracts were combined, washed with brine (250 mL), dried over Na_2SO_4, and concentrated under reduced pressure. The residue was purified by chromatography on silica gel (gradient elution: 20% EtOAc in hexanes to 50% EtOAc in hexanes) to afford **6** (5.461 g, 76%) as a pale-yellow oil.

^{1}H NMR (400 MHz, $CDCl_3$) δ = 8.52 (d, *J* = 1.8 Hz, 1H), 7.92 (d, *J* = 1.8 Hz, 1H), 5.22 (s, 1H), 4.34 – 4.23 (m, 4H), 3.62 – 3.44 (m, 2H), 3.38 – 3.19 (m, 2H), 1.32 – 1.10 (m, 12H) ppm.
^{13}C NMR (100 MHz, $CDCl_3$) δ = 166.62, 166.58, 152.5, 142.2, 138.9, 133.5, 121.9, 62.3, 59.9, 43.6, 39.8, 14.5, 14.1, 12.9 ppm.
LRMS (ES$^+$) *m/z* $[M + H]^+$ calcd for $C_{17}H_{24}BrN_2O_5^+$ 415.09; Found 415.19.
IR (diamond, ATR) ν 2980, 2937, 1735, 1631 cm^{-1}.

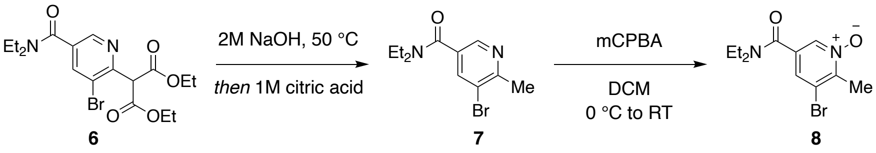

3-bromo-5-(diethylcarbamoyl)-2-methylpyridine 1-oxide (8)

To a solution of **6** (5.350 g, 12.92 mmol, 1.0 equiv) in MeOH (130 mL) was added 2M aqueous NaOH (32 mL). The solution was heated at 50°C and stirred for 16 h. To the resulting suspension, 1M aqueous citric acid (45 mL) was added to adjust the pH to 4, and the solution was stirred and heated at 60°C for 24 h. The solution was cooled to ambient temperature and the MeOH was removed by concentration under reduced pressure. The solution was added to H_2O (250 mL) and extracted with DCM (3 x 200 mL). The organic layers were combined, washed with brine (250 mL), dried over Na_2SO_4, and concentrated under reduced pressure.

To a 0°C cooled solution of the resulting residue in DCM (50 mL) was added mCPBA (70-75% balance) (5.946 g, 25.84 mmol, 2.0 equiv) slowly. The solution was warmed to ambient temperature and stirred for 22 h. The solution was added to 1M NaOH (150 mL) and the layers were separated. The aqueous layer was further extracted with 10% isopropyl alcohol in DCM (3 x 100 mL). The organic extracts were combined, dried over Na_2SO_4, filtered, and concentrated under reduced pressure. The residue was purified by column chromatography on silica gel (EtOAc then 10% MeOH in EtOAc) to afford **8** (3.469 g, 94%) as a white solid.

^{1}H NMR (400 MHz, $CDCl_3$) δ = 8.19 (d, *J* = 0.9 Hz, 1H), 7.4 (d, *J* = 0.9 Hz, 1H), 3.58 – 3.39 (m, 2H), 3.36 – 3.18 (m, 2H), 2.66 (s, 3H), 1.24 – 1.10 (m, 6H) ppm.
^{13}C NMR (100 MHz, $CDCl_3$) δ = 164.6, 150.2, 136.2, 133.0, 127.0, 122.1, 43.5, 39.9, 17.4, 14.4, 12.8 ppm.
LRMS (ES$^+$) *m/z* $[M + H]^+$ calcd for $C_{11}H_{16}BrN_2O_2^+$ 287.04; Found 287.12.
IR (diamond, ATR) ν 3455, 2972, 2935, 1632 cm^{-1}.

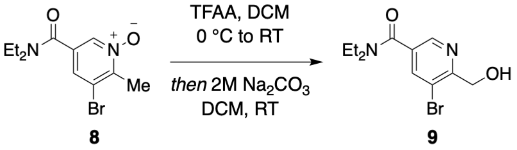

5-bromo-*N,N*-diethyl-6-(hydroxymethyl)nicotinamide (9)

To a 0°C cooled solution of **8** (1.301 g, 4.531 mmol, 1.0 equiv) in DCM (22.6 mL) was added trifluoroacetic anhydride (1.57 mL, 11.3 mmol, 2.5 equiv) dropwise. The solution was warmed to ambient temperature and stirred for 4 h before concentrating under reduced pressure. The residue was re-dissolved in DCM (22.6 mL) and 2M aqueous Na_2CO_3 (45.2 mL) was added. The biphasic solution was stirred vigorously at ambient temperature for 18 h, then poured into H_2O (100 mL). The layers were separated, and the aqueous layer was further extracted with DCM (3 x 50 mL). The organic extracts were combined, washed with brine (100 mL), dried over Na_2SO_4, and concentrated under reduced pressure. The residue was purified by column chromatography on silica gel (100% EtOAc) to afford **9** (1.119 g, 86%) as a yellow oil.

^{1}H NMR (400 MHz, $CDCl_3$) δ = 8.53 (d, *J* = 1.7 Hz, 1H), 7.90 (d, *J* = 1.7 Hz, 1H), 4.77 (d, *J* = 4.7 Hz, 2H), 4.23 (t, *J* = 4.7 Hz, 1H), 3.64 – 3.46 (m, 2H), 3.38 – 3.18 (m, 2H), 1.32 – 1.08 (m, 6H) ppm.
^{13}C NMR (100 MHz, $CDCl_3$) δ = 166.8, 157.6, 144.1, 138.6, 133.2, 118.7, 63.4, 43.6, 39.9, 14.5, 12.9 ppm.
LRMS (ES$^+$) *m/z* $[M + H]^+$ calcd for $C_{11}H_{16}BrN_2O_2$ 287.04; Found 287.12.
IR (diamond, ATR) ν 3412, 2972, 2934, 1624, 1588 cm^{-1}.

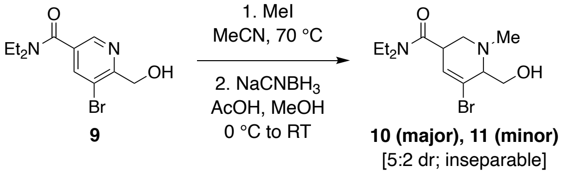

5-bromo-*N*,*N*-diethyl-6-(hydroxymethyl)-1-methyl-1,2,3,6-tetrahydropyridine-3-carboxamide (10, major diastereomer)

5- bromo-*N*,*N*-diethyl-6-(hydroxymethyl)-1-methyl-1,2,3,6-tetrahydropyridine-3-carboxamide (11, minor diastereomer)

To a solution of **9** (0.980 g, 3.413 mmol, 1.0 equiv) in MeCN (4.25 mL) was added MeI (1.28 mL, 20.5 mmol, 6.0 equiv). The vial was capped, and the solution was heated with stirring at 70°C for 24 h then subsequently cooled to ambient temperature. To the mixture was added EtOAc (8.5 mL) followed by hexanes (8.5 mL) with vigorous stirring. The suspension was cooled to 0°C, filtered, and washed with hexanes (2 x 5 mL). The resulting yellow solid was dried under reduced pressure and used directly in the next step.

To a 0°C cooled solution of the resulting methyl pyridinium salt (1.285 g, 2.995 mmol, 1.0 equiv) in MeOH (30 mL) was added AcOH (0.51 mL, 8.9 mmol, 3.0 equiv) followed by the dropwise addition of $NaCNBH_3$ (0.565 g, 8.98 mmol, 3.0 equiv) in MeOH (6 mL). The solution was warmed to ambient temperature and stirred for 16 h, then concentrated under reduced pressure. The residue was dissolved in EtOAc (100 mL) and added to 1M NaOH (200 mL). The layers were separated, and the aqueous layer was further extracted with EtOAc (3 x 100 mL). The organic extracts were combined and washed with brine (150 mL), dried over Na_2SO_4, and concentrated under reduced pressure. The residue was purified by column chromatography on silica gel (3% MeOH in DCM) to afford an inseparable mixture of diastereomers **10 (major diastereomer)** and **11 (minor diastereomer)** (0.726 g, 70%, 5:2 dr) as a pale yellow oil.

^{1}H NMR (400 MHz, $CDCl_3$) δ = 6.21† (s, 1H), 6.11* (d, *J* = 2.8 Hz, 0.4H), 3.94† (dd, *J* = 2.0 Hz, 1H), 3.88 – 3.78 (m, 1.4H), 3.70 – 3.64* (m, 0.4H), 3.59* (dd, *J* = 8.8, 11.6 Hz, 0.4H), 3.54 – 3.47† (m, 1H), 3.34 (quint, *J* = 7.3 Hz, 5.6H), 3.21* (dd, *J* = 9.4, 13.6 Hz, 0.4H), 3.14 – 3.08* (m, 0.4H), 3.02 – 2.92† (m, 2H), 2.88 – 2.80† (m, 1H), 2.76* (dd, *J* = 5.1, 14 Hz, 0.4H), 2.55* (s, 1.2H), 2.45† (s, 3H), 2.24 – 2.14 (m, 4.2H), 1.09 (t, *J* = 7.1 Hz, 4.2H)
^{13}C NMR (100 MHz, $CDCl_3$) δ = 170.22, 170.15, 130.4, 127.6, 123.3, 122.9, 68.6, 67.6, 60.9, 59.6, 53.6, 47.0, 43.5, 42.9, 42.2, 42.1, 41.3, 40.7, 40.4, 36.7, 15.1, 14.9, 13.2, 13.1 ppm.
LRMS (ES$^+$) *m/z* $[M + H]^+$ calcd for $C_{12}H_{22}BrN_2O_2^+$ 305.09; Found 305.14.
IR (diamond, ATR) ν 3418, 2970, 2934, 2799, 1629 cm^{-1}.

†denotes ^{1}H NMR signal arising exclusively from the major diastereomer; *denotes ^{1}H NMR signal arising exclusively from the minor diastereomer; undesignated signals arise from a mixture of both.

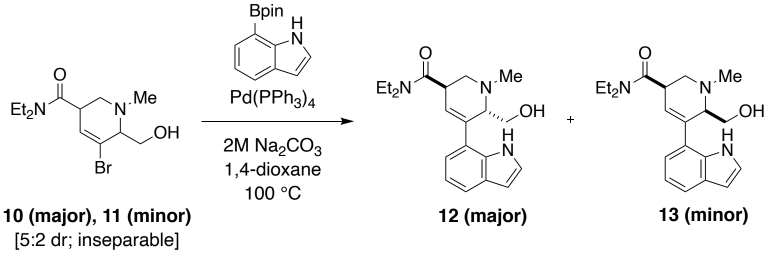

***N*,*N*-diethyl-6-(hydroxymethyl)-5-(1*H*-indol-7-yl)-1-methyl-1,2,3,6-tetrahydropyridine-3-carboxamide (12, major diastereomer, assigned anti stereochemistry based on the crystal structure of 16)**

***N*,*N*-diethyl-6-(hydroxymethyl)-5-(1*H*-indol-7-yl)-1-methyl-1,2,3,6-tetrahydropyridine-3-carboxamide (13, minor diastereomer, assigned syn stereochemistry based on the assignment of 12)**

A mixture of diastereomers **10** and **11** (5:2 dr) (0.698 g, 2.29 mmol, 1.0 equiv), 1,4-dioxane (22.9 mL), indole-7-boronic acid pinacol ester (0.834 g, 3.43 mmol, 1.5 equiv), and 2M aqueous Na_2CO_3 (2.29 mL) were added to a vial and the solution was sparged with N_2 for 10 min before the addition of $Pd(PPh_3)_4$ (0.132 g, 0.114 mmol, 0.05 equiv). The vial was capped and the mixture was heated with stirring at 100°C in a preheated oil bath for 4 h. The mixture was cooled to ambient temperature, added to H_2O (400 mL), and extracted with EtOAc (3 x 150 mL). The organic extracts were combined, washed

with brine (200 mL), dried over Na_2SO_4, and concentrated under reduced pressure. The residue was purified by column chromatography on silica gel (gradient elution: 2% MeOH in DCM to 10% MeOH in DCM) to afford **12 (major diastereomer, assigned anti stereochemistry)** (0.398 g, 51%) and **13 (minor diastereomer, assigned syn stereochemistry)** (0.148 g, 19%) as off-white semi-solids.

(Major Diastereomer, 12)
^{1}H NMR (400 MHz, $CDCl_3$) δ = 9.46 (s, 1H), 7.56 (d, *J* = 7.8 Hz, 1H), 7.22 (t, *J* = 2.8 Hz, 1H), 7.06 (t, *J* = 7.3 Hz, 1H), 7.00 (dd, *J* = 0.9, 8.4 Hz, 1H), 6.53 (dd, *J* = 2.1, 3.2 Hz, 1H), 5.92 (s, 1H), 3.79 (dd, *J* = 3.0, 11.2 Hz, 1H), 3.74 – 3.66 (m, 1H), 3.50 – 3.28 (m, 5H), 3.26 – 3.08 (m, 2H), 3.07 – 2.96 (m, 1H), 2.78 (br s, 1H), 2.52 (s, 2H), 1.23 (t, *J* = 7.1 Hz, 3H), 1.10 (t, *J* = 7.1 Hz, 3H) ppm.
^{13}C NMR (100 MHz, $CDCl_3$) δ = 171.7, 137.3, 135.4, 128.11, 128.05, 124,9, 124.1, 121.4, 119.9, 119.4, 102.4, 66.8, 59.1, 54.0, 43.2, 42.0, 40.3, 39.3, 14.9, 13.1 ppm.
LRMS (ES$^+$) *m/z* $[M + H]^+$ calcd for $C_{20}H_{28}N_3O_2^+$ 342.22; Found 342.32.
IR (diamond, ATR) ν 3267, 2970, 2932, 1615 cm^{-1}.

(Minor Diastereomer, 13)
^{1}H NMR (400 MHz, $CDCl_3$) δ = 9.86 (s, 1H), 7.55 (d, *J* = 7.8 Hz, 1H), 7.29 – 7.24 (m, 1H), 7.07 (t, *J* = 7.4 Hz, 1H), 6.98 (dd, *J* = 0.6, 7.6 Hz, 1H), 6.51 (dd, *J* = 2.1 Hz, 3.2 Hz, 1H), 6.08 – 6.04 (m, 1H0, 3.76 – 3.68 (m, 1H), 3.64 – 3.28 (m, 8H), 3.15 – 3.06 (m 1H), 2.99 (dd, *J* = 5.7, 13.2 Hz, 1H), 2.66 (s, 3H), 1.26 (t, *J* = 7.2 Hz, 3H), 1.17 (t, *J* = 7.1 Hz, 3H) ppm.
^{13}C NMR (100 MHz, $CDCl_3$) δ = 173.0, 135.7, 135.0, 128.4, 125.6, 125.4, 123.8, 120.0, 119.4, 119.0, 102.1, 64.8, 60.9, 48.6, 42.7, 42.3, 40.6, 34.8, 15.1, 13.3 ppm.
LRMS (ES$^+$) *m/z* $[M + H]^+$ calcd for $C_{20}H_{28}N_3O_2^+$ 342.22; Found 342.32.
IR (diamond, ATR) ν 3270, 2973, 2934, 1613 cm^{-1}.

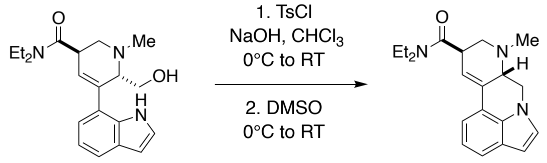

12 14

***N,N*-diethyl-8-methyl-7a,8,9,10-tetrahydro-7*H*-indolo[7,1-*fg*][1,7]naphthyridine-10-carboxamide (14)**

To a 0°C cooled solution of **12** (0.250 g, 0.732 mmol, 1.0 equiv) in $CHCl_3$ (7.3 mL) was added freshly crushed NaOH (0.234 g, 5.86 mmol, 8.0 equiv). A solution of TsCl (0.167 g, 0.878 mmol, 1.2 equiv) in $CHCl_3$ (1.5 mL) was added dropwise over 10 minutes. The mixture was warmed to ambient temperature and stirred for 1.5 h. The mixture was cooled to 0°C, and DMSO (3.7 mL) was added slowly before warming to ambient temperature and stirring for 1 h. The mixture was partitioned in H_2O (250 mL) and EtOAc (200 mL) and the layers were separated. The aqueous layer was further extracted with EtOAc (3 x 100 mL). The organic extracts were combined, washed with brine (250 mL), dried over Na_2SO_4, and concentrated under reduced pressure. The residue was purified by column chromatography on silica gel (gradient elution: 8% MeOH in EtOAc to 12% MeOH in EtOAc) to afford **14** (0.168 g, 71 %) as an off white semi-solid.

^{1}H NMR (400 MHz, $CDCl_3$) δ = 7.5 (d, *J* = 7.9 Hz, 1H), 7.31 (d, *J* = 7.3 Hz, 1H), 7.08 – 7.04 (m, 2H), 6.46 (d, *J* = 3.0 Hz, 1H), 6.31 (s, 1H), 4.66 (dd, *J* = 5.4, 11.2 Hz, 1H), 3.90 – 3.82 (m, 1H), 3.80 (t, *J* = 11.1 Hz, 1H), 3.54 – 3.40 (m, 5H), 3.05 (dd, *J* = 5.0, 11.2 Hz, 1H), 2.95 (t, *J* = 10.7 Hz, 1H), 2.59 (s, 3H), 1.26 (t, *J* = 7.1Hz, 3H), 1.18 (t, *J* = 7.1Hz, 3H) ppm.
^{13}C NMR (100 MHz, CDCl) δ = 171.2, 133.2, 132.5, 126.3, 126.2, 120.3, 120.0, 118.91, 118.88, 114.9, 101.3, 60.5, 55.8, 48.0, 44.0, 42.1, 40.3, 39.9, 15.0, 13.2 ppm.
LRMS (ES$^+$) *m/z* $[M + H]^+$ calcd for $C_{20}H_{26}N_3O^+$ 324.21; Found 324.29.
IR (diamond, ATR) ν 2972, 2869, 2798, 1636 cm^{-1}.

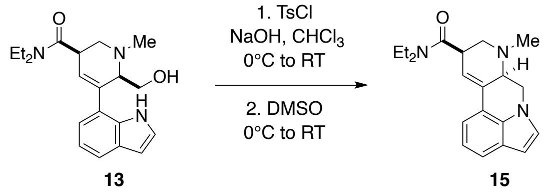

N,*N*-diethyl-8-methyl-7a,8,9,10-tetrahydro-7*H*-indolo[7,1-*fg*][1,7]naphthyridine-10-carboxamide (15)

To a 0°C cooled solution of **13** (0.130 g, 0.381 mmol, 1.0 equiv) in $CHCl_3$ (3.8 mL) was added freshly crushed NaOH (0.122 g, 3.05 mmol, 8.0 equiv). A solution of TsCl (0.087 g, 0.46 mmol, 1.2 equiv) in $CHCl_3$ (0.76 mL) was added dropwise over 10 minutes. The mixture was warmed to ambient temperature and stirred for 1.5 h. The mixture was cooled to 0°C, and DMSO (1.9 mL) was added slowly before warming to ambient temperature and stirring for 3 h. The mixture was partitioned in H_2O (200 mL) and EtOAc (150 mL) and the layers were separated. The aqueous layer was further extracted with EtOAc (3 x 50 mL). The organic extracts were combined, washed with brine (100 mL), dried over Na_2SO_4, and concentrated under reduced pressure. The residue was purified by column chromatography on silica gel (gradient elution: 8% MeOH in EtOAc to 12% MeOH in EtOAc) to afford **15** (0.044 g, 36%) as a brown semi-solid.

^{1}H NMR (400 MHz, $CDCl_3$) δ = 7.48 (d, *J* = 7.9 Hz, 1H), 7.22 (d, *J* = 7.2 Hz, 1H), 7.08 – 7.02 (m, 2H), 6.45 (d, *J* = 3.0 Hz, 1H), 6.37 (dd, *J* = 2.0, 3.6 Hz, 1H), 4.50 (dd, *J* = 5.5, 11.2 Hz, 1H), 4.02 (t, *J* = 11.2 Hz, 1H), 3.66 – 3.60 (m, 1H), 3.56 – 3.30 (m, 5H), 3.15 (dd, *J* = 5.7, 12.2 Hz, 1H), 2.83 (dd, *J* = 4.8, 12.2 Hz, 1H), 2.62 (s, 3H), 1.27 (t, *J* = 7.0 Hz, 3H), 1.13 (t, *J* = 7.0 Hz, 3H) ppm.
^{13}C NMR (100 MHz, CDCl) δ = 171.5, 133.4, 133.4, 126.5, 126.2, 120.4, 120.1, 119.9, 118.9, 114.2, 101.2, 58.6, 52.5, 47.9, 43.6, 42.0, 40.3, 37.4, 15.0, 13.2 ppm.
LRMS (ES$^+$) *m/z* [M + H]$^+$ calcd for $C_{20}H_{26}N_3O^+$ 324.21; Found 324.29.
IR (diamond, ATR) ν 2969, 2932, 2871, 2791, 1634 cm^{-1}.

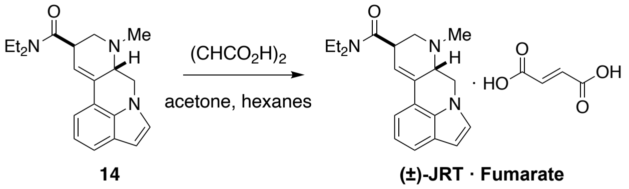

(±)-JRT • Fumarate

To a solution of fumaric acid (0.051 g, 0.438 mmol, 1.05 equiv) in acetone (6 mL) stirring at 50 °C was added **14** (0.135 g, 0.417 mmol, 1.0 equiv.) in acetone (2 mL) slowly. The solution was cooled to room temperature slowly with stirring, and hexanes (15 mL) was added slowly. The suspension was cooled to 0 °C for 1 hour and then subsequently in a -20 °C freezer overnight. The resulting mixture was filtered, washed with ice-cold 1:1 acetone/hexanes (2 mL) and dried in a vacuum oven at 50°C to afford (±)-JRT • fumarate (1:1 salt, 0.126 g, 69%) as a beige solid.

^{1}H NMR (400 MHz, $MeOD_4$) δ = 7.45 (d, *J* = 7.8 Hz, 1H), 7.30 (d, *J* = 7.3 Hz, 1H), 7.21 (d, *J* = 3.1 Hz, 1H), 7.03 (t, *J* = 7.6 Hz, 1H), 6.74 (s, 2H), 6.44 (d, *J* = 3.04 Hz, 1H), 6.36 (s, 1H), 4.94 – 4.86 (m, 1H), 4.08 – 4.00 (m, 1H), 3.80 (t, *J* = 11.1 Hz, 1H), 3.72 – 3.65 (m, 1H), 3.56 (q, *J* = 7.2 Hz, 2H), 3.45 (septet, *J* = 7.6 Hz, 2H), 3.29 – 3.23 (m, 1H), 2.99 (t, *J* = 11.1 Hz, 1H), 2.74 (s, 3H), 1.30 (t, *J* = 7.1 Hz, 3H), 1.18 (t, *J* = 7.1 Hz, 3H) ppm.
^{13}C NMR (100 MHz, $MeOD_4$) δ = 173.0, 168.8, 135.4, 134.4, 133.1, 128.0, 127.8, 121.32, 121.26, 119.2, 119.1, 115.8, 102.4, 61.6, 56.4, 47.7, 43.8, 43.6, 42.0, 40.2, 15.1, 13.3 ppm.
LRMS (ES$^+$) *m/z* [M + H]$^+$ calcd for $C_{20}H_{26}N_3O^+$ 324.21; Found 324.35.
IR (diamond, ATR) ν 2971, 2869, 2799, 1630 cm^{-1}.

Chiral Separation of (+)-JRT and (–)-JRT.

Racemic (±)-JRT (**14**) was separated into its enantiomers by preparatory chiral HPLC using an Agilent 1260 Infinity II (Chiralpak-IC 250×30 mm, 5 μm; eluant: 50:50 mixture of 0.1% diethylamine in n-hexane (v/v) and 50% MeOH in CH_2Cl_2 (v/v); 35.0 mL/min).

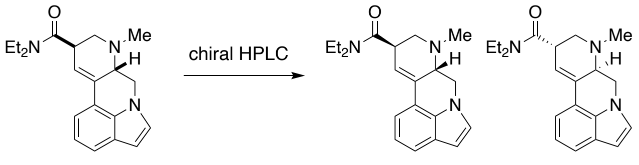

14 **(+)-JRT** **(-)-JRT**

Analytical HPLC of (±)-JRT:

Injection Volume : 15 uL
Data File Name : 01042021B1.lcd
Method File Name : CHIRAL-MET-B50-1.0mL.lcm
Report File Name : Default.lcr
Data Acquired : 01-04-2021 15:50:44
Description : COLUMN::CHIRAL PAK IC(250mmX 4.6mm,5μm)
Mobile Phase A :0.1%DEA in n-Hexane
Mobile Phase B :IPA
A:B:50:50
Flow:1.0mL/min

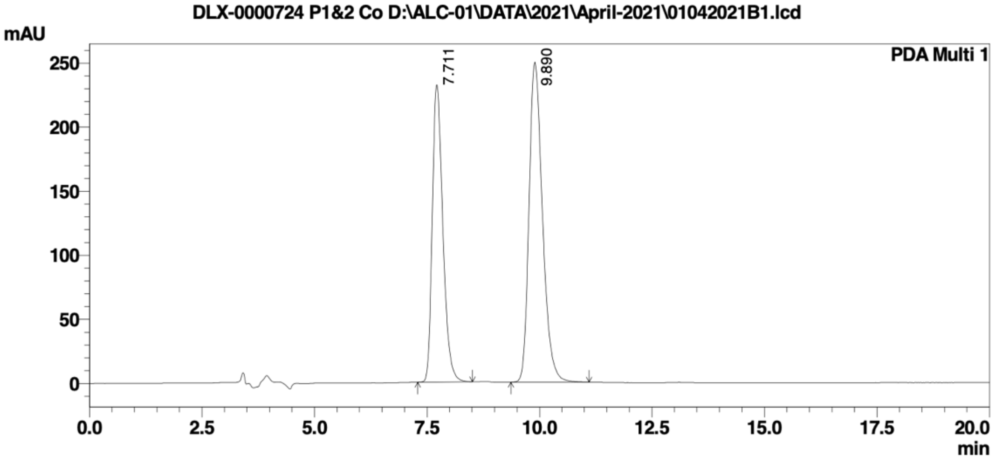

1 PDA Multi 1/240nm 4nm

PeakTable

PDA Ch1 240nm 4nm

Peak#	Ret. Time	Area	Height	Area %
1	7.711	3788998	231995	42.180
2	9.890	5193924	249782	57.820
Total		8982922	481778	100.000

Analytical HPLC of (–)-JRT (first eluting peak):

Injection Volume : 15 uL
Data File Name : 01042021B2.lcd
Method File Name : CHIRAL-MET-B50-1.0mL.lcm
Report File Name : Default.lcr
Data Acquired : 01-04-2021 16:16:22
Description : COLUMN::CHIRAL PAK IC(250mmX 4.6mm,5µm)
Mobile Phase A :0.1%DEA in n-Hexane
Mobile Phase B :IPA
A:B:50:50
Flow:1.0mL/min

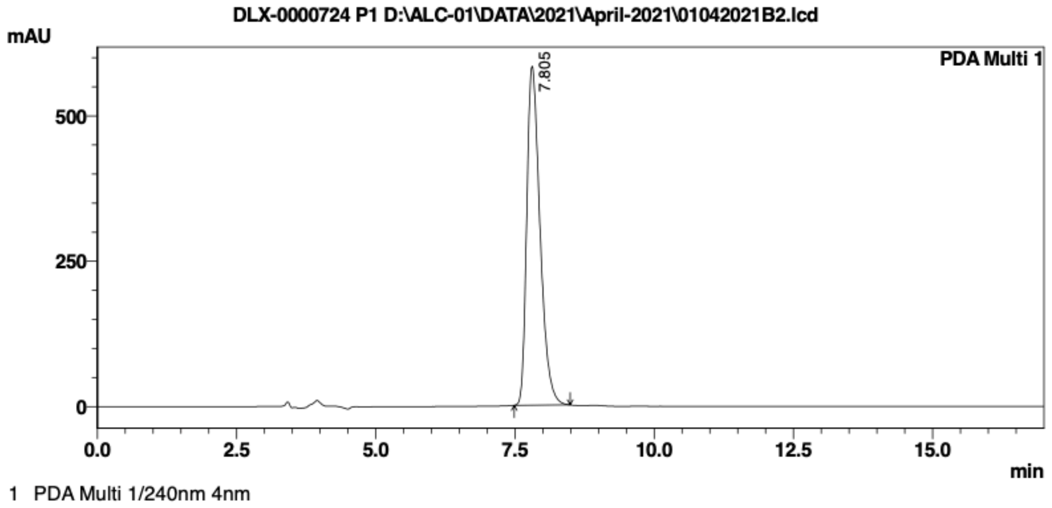

PeakTable

PDA Ch1 240nm 4nm

Peak#	Ret. Time	Area	Height	Area %
1	7.805	9642643	582879	100.000
Total		9642643	582879	100.000

First eluting peak. Analytical chiral HPLC R_t = 7.81 min (Chiralpak-IC 250×4.6 mm, 5 µm; Eluant: 50:50 mixture of 0.1% DEA in n-hexane and isopropanol, 1.0 mL/min). 250 mg, Pale brown semisolid; LC-MS: *m/z* = 324.1 $[M+H]^+$

(–)-JRT • Fumarate

In a sealed tube, fumaric acid (81 mg, 0.69 mmol, 1.0 equiv) in acetone (0.81 mL) was heated to 40°C and stirred for 1 hr. To the resulting clear solution was added (–)-JRT (226 mg, 0.69 mmol, 1.0 equiv) dissolved in acetone (1.13 mL), and the mixture was stirred for 2 h at 40°C. After cooling to room temperature, the volatiles were evaporated to yield a residue which was triturated with diethyl ether followed by n-pentane to afford a pale brown semi solid, which was further lyophilized to yield 270 mg of (–)-JRT as the 1:1 fumarate salt (pale brown solid). The NMR data were consistent with those reported for (±)-JRT.

Specific Rotation $[\alpha]_D^{20}$ -9.9 (c = 0.0011 in ethanol).

Analytical HPLC of (+)-JRT (second eluting peak):

Injection Volume : 10 uL
Data File Name : 01042021B3.lcd
Method File Name : CHIRAL-MET-B50-1.0mL.lcm
Report File Name : Default.lcr
Data Acquired : 01-04-2021 16:34:43
Description : COLUMN::CHIRAL PAK IC(250mmX 4.6mm,5µm)
Mobile Phase A :0.1%DEA in n-Hexane
Mobile Phase B :IPA
A:B:50:50
Flow:1.0mL/min

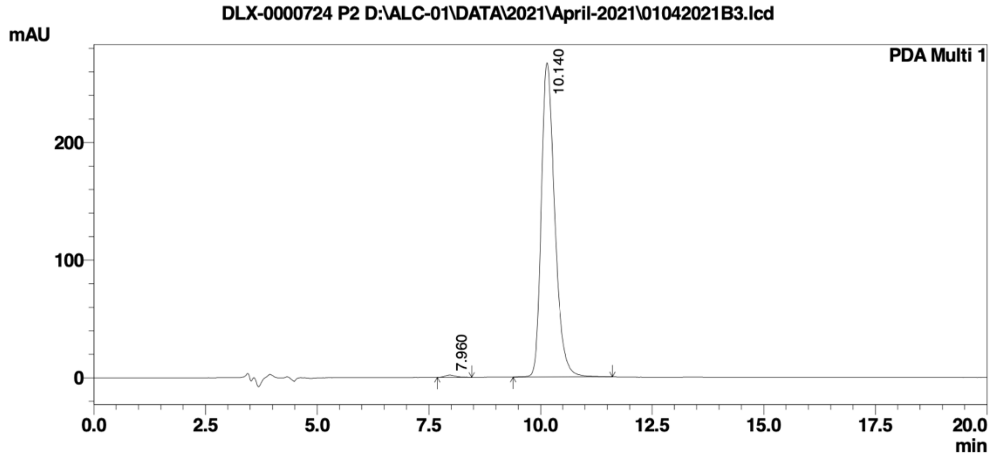

1 PDA Multi 1/240nm 4nm

PeakTable

PDA Ch1 240nm 4nm

Peak#	Ret. Time	Area	Height	Area %
1	7.960	31705	1915	0.561
2	10.140	5619691	267077	99.439
Total		5651396	268993	100.000

Second eluting peak. Analytical chiral HPLC R_t = 10.14 min (Chiralpak-IC 250×4.6 mm, 5 µm; Eluant: 50:50 mixture of 0.1% DEA in n-hexane and isopropanol, 1.0 mL/min). 290 mg, Pale brown semi solid; LC-MS**:** *m/z* = 324.1 $[M+H]^+$

(+)-JRT • Fumarate

In a sealed tube, fumaric acid (95 mg, 0.82 mmol, 1.0 equiv) was added to acetone (0.95 mL), heated to 40°C, and stirred for 1 hr. The resulting clear solution was treated with (+)-JRT (266 mg, 0.82 mmol, 1.0 equiv) dissolved in acetone (1.33 mL) and stirred for 2 hr at 40°C. After cooling to room temperature, the volatiles were evaporated to afford a residue which was triturated with diethyl ether followed by n-pentane to yield a pale brown solid, which was further lyophilized to yield 250 mg of (+)-JRT as the 1:1 fumarate salt (pale brown solid). The NMR data were consistent with those reported for (±)-JRT.

Specific Rotation $[\alpha]_D^{20}$ +8.7 (c = 0.0011 in ethanol).

(7a*S*,10*R*)-10-(diethylcarbamoyl)-8,8-dimethyl-7a,8,9,10-tetrahydro-7*H*-indolo[7,1-*fg*][1,7]naphthyridin-8-ium iodide (16)

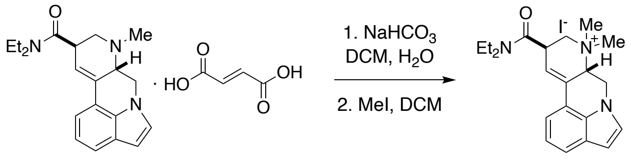

(+)-JRT · Fumarate **16**

A mixture of (+)-JRT • fumarate (0.030 g), saturated aqueous $NaHCO_3$ (30 mL), and DCM (30 mL) was stirred vigorously for 20 min. The layers were separated, and the aqueous layer was further extracted with DCM (2 x 30 mL). The combined organic extracts were washed with brine (1 x 50 mL), dried over Na_2SO_4, and concentrated under reduced pressure. The resulting freebase was dissolved in DCM (4 mL), then methyl iodide (0.4 mL) was added, and the solution was stirred at ambient temperature for 14 hours in a 1 dram vial. Upon reaction completion, the stir bar was removed, and the 1 dram vial was placed inside of a 20 mL scintillation vial containing a solution of hexanes (5 mL) and Et_2O (5 mL) as antisolvents for vapor diffusion. The two-chamber system was sealed by capping only the outer 20 mL scintillation vial and allowed to sit in the dark at ambient temperature for 1 month, with crystals suitable for x-ray diffraction analysis precipitating during this time. A single crystal was used for x-ray analysis, and the remainder was filtered and washed with ice-cold Et_2O (1 mL) and dried in vacuo to afford **16** (0.019 g, 60 %) as a light brown crystalline solid.

^{1}H NMR (400 MHz, $CDCl_3$) δ = 7.55 (d, *J* = 7.0 Hz, 1H), 7.48 (d, *J* = 7.5 Hz, 1H), 7.32 (d, *J* = 2.9 Hz, 1H), 7.12 (t, *J* = 7.7 Hz, 1H), 6.63 (s, 1H), 6.54 (d, *J* = 3.0 Hz, 1H), 5.18 – 5.10 (m, 1H), 5.06 – 4.99 (m, 1H), 4.34 – 4.26 (m, 1H), 4.21 (t, *J* = 11.3 Hz, 1H), 4.06 (t, *J* = 11.4 Hz, 1H), 3.99 – 3.92 (m, 1H), 3.76 – 3.62 (m, 2H), 3.59 (s, 3H), 3.56 – 3.40 (m, 2H), 3.35 (s, 3H), 1.38 (t, *J* = 3.1 Hz, 3H), 1.20 (t, *J* = 3.1 Hz, 3H) ppm.
LRMS (ES$^+$) *m/z* $[M]^+$ calcd for $C_{21}H_{28}N_3O^+$ 338.22; Found 338.45.
IR (diamond, ATR) ν 3456, 2971, 2932, 1633 cm^{-1}.

X-Ray Crystallography

An orange block with approximate orthogonal dimensions 0.248 x 0.448 x 0.594mm^3 was placed and optically centered on the Bruker Duo (*39*) APEXII CCD system at –183°C(90K). Indexing of the unit cell used a random set of reflections collected from three series of 0.5° wide ω-scans, 10 seconds per frame, and 30 frames per series that were well distributed in reciprocal space. Five ω-scan data frame series were collected [MoK_α] with 0.3° wide scans, 15 seconds per frame and 606 frames collected per series at varying φ angles (φ=0°, 72°, 144°, 216°, 288°). The crystal to detector distance was 5.15 cm, thus providing a complete sphere of data to $2\theta_{max}$=61.40°.

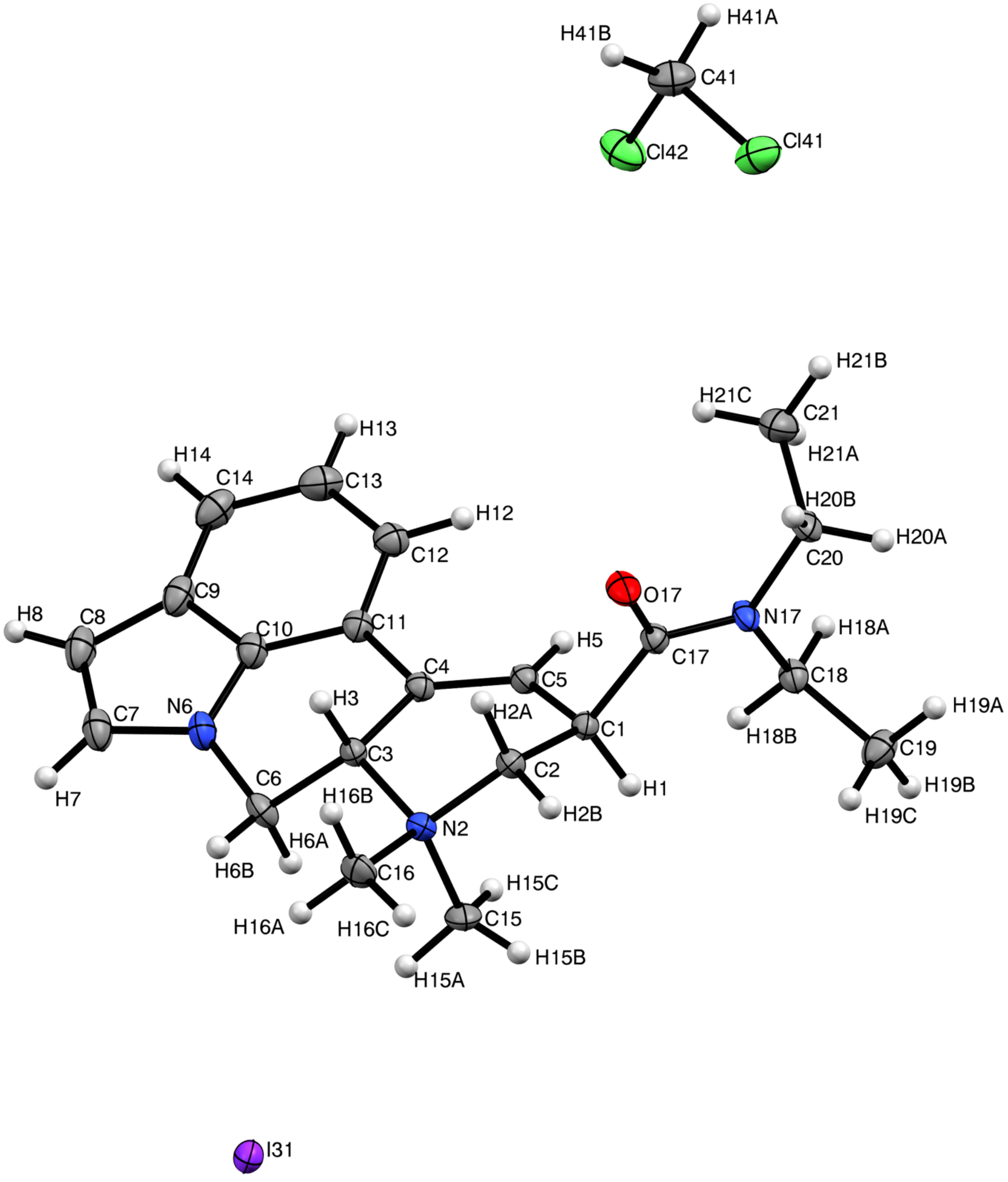

X-Ray Crystal Structure of 16. One molecule of **16** and one solvent molecule of DCM are present. Atomic numbering matches the provided atomic coordinates.

Crystal Data and Structure refinement for **16**

Empirical Formula	$C_{22}H_{30}Cl_2IN_3O$	
Formula Weight	550.29	
Temperature	90(2) K	
Wavelength	0.71073 Å	
Crystal System	Orthorhombic	
Space Group	$P2_12_12_1$	
Unit Cell Dimensions	a = 7.0716(6) Å	α = 90°
	b = 14.4326(12) Å	β = 90°
	c = 23.0876(19) Å	γ = 90°
Volume	2356.4(3) $Å^3$	
Z	4	
ρ_{calcd}	1.551 g/cm^3	
Absorption Coefficient (*μ*)	1.604 mm^{-1}	
F(000)	1112	
Crystal Size	0.594 x 0.448 x 0.248 mm	
Crystal Color and Shape	Orange Block	
Diffractometer	Bruker APEX-II CCD	
θ Range for Data Collection	1.664 to 30.735°	
Index Ranges	-10<=h<=10, -20<=k<=20, -33<=l<=33	
Reflections Collected	28854	
Independent Reflections	7321 [R(int) = 0.0163]	
Observed Reflections (I>2σ(I))	7222	
Completeness to *θ* = 25.242°	99.8 %	
Absorption Correction	Semi-empirical from equivalents	
Max and Min. Transmission	0.6095 and 0.4862	
Solution Method	SHELXT	
Refinement Method	SHELXL-2018/3 Full-matrix least-squares on F^2	
Data / Restraints / Parameters	7321 / 0 / 266	
Goodness-of-fit on F^2	1.081	
Final R Indices [I>2σ(I)]	R1 = 0.0184, wR2 = 0.0493	
R Indices (all data)	R1 = 0.0189, wR2 = 0.0496	
Absolute Structure Parameters	Flack = -0.014(3); Parsons = -0.013(3); Hooft = -0.013(2)	
Largest Diff. Peak and Hole	1.009 and -0.672 e·Å^{-3}	

Non-hydrogen atomic coordinates (x 10^4) and equivalent isotropic displacement parameters ($Å^2$x 10^3) for **16**. U_{eq} is defined as one third of the trace of the orthogonalized U^{ij} tensor.

Label	x	y	z	U_{eq}
C(1)	7844(3)	5098(1)	6685(1)	14(1)
C(2)	7093(3)	4697(1)	7247(1)	15(1)
N(2)	6608(3)	3678(1)	7180(1)	16(1)
C(3)	4913(3)	3611(1)	6779(1)	15(1)
C(4)	5239(3)	4172(1)	6228(1)	15(1)
C(5)	6606(3)	4811(1)	6184(1)	16(1)
C(6)	4371(3)	2587(2)	6656(1)	20(1)
N(6)	2719(3)	2616(1)	6285(1)	21(1)
C(7)	1281(4)	1975(2)	6207(1)	24(1)
C(8)	240(4)	2202(2)	5728(1)	26(1)
C(9)	1056(3)	3026(2)	5484(1)	22(1)
C(10)	2603(3)	3249(2)	5844(1)	19(1)
C(11)	3804(3)	4007(2)	5774(1)	17(1)
C(12)	3393(3)	4572(2)	5301(1)	21(1)
C(13)	1868(4)	4372(2)	4928(1)	25(1)
C(14)	694(4)	3612(2)	5013(1)	26(1)
C(15)	8299(3)	3145(2)	6965(1)	22(1)
C(16)	6084(4)	3316(2)	7770(1)	22(1)
C(17)	7780(3)	6165(1)	6732(1)	16(1)
N(17)	8884(3)	6659(1)	6369(1)	17(1)
O(17)	6664(2)	6533(1)	7070(1)	22(1)
C(18)	10359(3)	6288(2)	5986(1)	17(1)
C(19)	12324(3)	6395(2)	6245(1)	24(1)
C(20)	8679(3)	7674(2)	6374(1)	20(1)
C(21)	7253(4)	8000(2)	5926(1)	29(1)
I(31)	8094(1)	198(1)	7065(1)	19(1)
C(41)	1778(4)	9810(2)	5726(1)	29(1)
Cl(41)	3738(1)	10001(1)	6186(1)	30(1)
Cl(42)	2063(1)	8775(1)	5312(1)	32(1)

Bond lengths (Å) for **16.**

Bond	Length	Bond	Length
C(1)-C(5)	1.510(3)	C(12)-H(12)	0.9500
C(1)-C(2)	1.515(3)	C(13)-C(14)	1.390(4)
C(1)-C(17)	1.544(3)	C(13)-H(13)	0.9500
C(1)-H(1)	1.0000	C(14)-H(14)	0.9500
C(2)-N(2)	1.518(3)	C(15)-H(15A)	0.9800
C(2)-H(2A)	0.9900	C(15)-H(15B)	0.9800
C(2)-H(2B)	0.9900	C(15)-H(15C)	0.9800
N(2)-C(16)	1.505(3)	C(16)-H(16A)	0.9800
N(2)-C(15)	1.507(3)	C(16)-H(16B)	0.9800
N(2)-C(3)	1.517(3)	C(16)-H(16C)	0.9800
C(3)-C(4)	1.526(3)	C(17)-O(17)	1.230(3)
C(3)-C(6)	1.553(3)	C(17)-N(17)	1.350(3)
C(3)-H(3)	1.0000	N(17)-C(18)	1.468(3)
C(4)-C(5)	1.340(3)	N(17)-C(20)	1.471(3)
C(4)-C(11)	1.478(3)	C(18)-C(19)	1.521(3)
C(5)-H(5)	0.9500	C(18)-H(18A)	0.9900
C(6)-N(6)	1.450(3)	C(18)-H(18B)	0.9900
C(6)-H(6A)	0.9900	C(19)-H(19A)	0.9800
C(6)-H(6B)	0.9900	C(19)-H(19B)	0.9800
N(6)-C(10)	1.370(3)	C(19)-H(19C)	0.9800
N(6)-C(7)	1.386(3)	C(20)-C(21)	1.519(3)
C(7)-C(8)	1.369(4)	C(20)-H(20A)	0.9900
C(7)-H(7)	0.9500	C(20)-H(20B)	0.9900
C(8)-C(9)	1.436(4)	C(21)-H(21A)	0.9800
C(8)-H(8)	0.9500	C(21)-H(21B)	0.9800
C(9)-C(14)	1.400(4)	C(21)-H(21C)	0.9800
C(9)-C(10)	1.412(3)	C(41)-Cl(41)	1.767(3)
C(10)-C(11)	1.394(3)	C(41)-Cl(42)	1.785(3)
C(11)-C(12)	1.394(3)	C(41)-H(41A)	0.9900
C(12)-C(13)	1.411(3)	C(41)-H(41B)	0.9900

Bond angles (°) for **16.**

Bonds	Angle	Bonds	Angle
C(5)-C(1)-C(2)	110.39(17)	C(13)-C(12)-H(12)	119.4
C(5)-C(1)-C(17)	108.07(16)	C(14)-C(13)-C(12)	122.1(2)
C(2)-C(1)-C(17)	108.11(16)	C(14)-C(13)-H(13)	118.9
C(5)-C(1)-H(1)	110.1	C(12)-C(13)-H(13)	118.9
C(2)-C(1)-H(1)	110.1	C(13)-C(14)-C(9)	118.6(2)
C(17)-C(1)-H(1)	110.1	C(13)-C(14)-H(14)	120.7
C(1)-C(2)-N(2)	111.23(16)	C(9)-C(14)-H(14)	120.7
C(1)-C(2)-H(2A)	109.4	N(2)-C(15)-H(15A)	109.5
N(2)-C(2)-H(2A)	109.4	N(2)-C(15)-H(15B)	109.5
C(1)-C(2)-H(2B)	109.4	H(15A)-C(15)-H(15B)	109.5
N(2)-C(2)-H(2B)	109.4	N(2)-C(15)-H(15C)	109.5
H(2A)-C(2)-H(2B)	108.0	H(15A)-C(15)-H(15C)	109.5
C(16)-N(2)-C(15)	108.40(17)	H(15B)-C(15)-H(15C)	109.5
C(16)-N(2)-C(3)	109.53(17)	N(2)-C(16)-H(16A)	109.5
C(15)-N(2)-C(3)	113.19(16)	N(2)-C(16)-H(16B)	109.5
C(16)-N(2)-C(2)	107.51(16)	H(16A)-C(16)-H(16B)	109.5
C(15)-N(2)-C(2)	110.45(16)	N(2)-C(16)-H(16C)	109.5
C(3)-N(2)-C(2)	107.62(15)	H(16A)-C(16)-H(16C)	109.5
N(2)-C(3)-C(4)	110.78(16)	H(16B)-C(16)-H(16C)	109.5
N(2)-C(3)-C(6)	111.57(17)	O(17)-C(17)-N(17)	122.42(19)
C(4)-C(3)-C(6)	112.92(17)	O(17)-C(17)-C(1)	119.62(18)
N(2)-C(3)-H(3)	107.1	N(17)-C(17)-C(1)	117.83(18)
C(4)-C(3)-H(3)	107.1	C(17)-N(17)-C(18)	126.27(17)
C(6)-C(3)-H(3)	107.1	C(17)-N(17)-C(20)	117.67(18)
C(5)-C(4)-C(11)	123.47(19)	C(18)-N(17)-C(20)	115.97(17)
C(5)-C(4)-C(3)	122.59(18)	N(17)-C(18)-C(19)	112.04(18)
C(11)-C(4)-C(3)	113.70(17)	N(17)-C(18)-H(18A)	109.2
C(4)-C(5)-C(1)	123.23(18)	C(19)-C(18)-H(18A)	109.2
C(4)-C(5)-H(5)	118.4	N(17)-C(18)-H(18B)	109.2
C(1)-C(5)-H(5)	118.4	C(19)-C(18)-H(18B)	109.2
N(6)-C(6)-C(3)	106.28(18)	H(18A)-C(18)-H(18B)	107.9
N(6)-C(6)-H(6A)	110.5	C(18)-C(19)-H(19A)	109.5
C(3)-C(6)-H(6A)	110.5	C(18)-C(19)-H(19B)	109.5
N(6)-C(6)-H(6B)	110.5	H(19A)-C(19)-H(19B)	109.5
C(3)-C(6)-H(6B)	110.5	C(18)-C(19)-H(19C)	109.5
H(6A)-C(6)-H(6B)	108.7	H(19A)-C(19)-H(19C)	109.5

C(10)-N(6)-C(7)	107.8(2)	H(19B)-C(19)-H(19C)	109.5
C(10)-N(6)-C(6)	120.42(18)	N(17)-C(20)-C(21)	111.67(19)
C(7)-N(6)-C(6)	130.4(2)	N(17)-C(20)-H(20A)	109.3
C(8)-C(7)-N(6)	109.8(2)	C(21)-C(20)-H(20A)	109.3
C(8)-C(7)-H(7)	125.1	N(17)-C(20)-H(20B)	109.3
N(6)-C(7)-H(7)	125.1	C(21)-C(20)-H(20B)	109.3
C(7)-C(8)-C(9)	107.4(2)	H(20A)-C(20)-H(20B)	107.9
C(7)-C(8)-H(8)	126.3	C(20)-C(21)-H(21A)	109.5
C(9)-C(8)-H(8)	126.3	C(20)-C(21)-H(21B)	109.5
C(14)-C(9)-C(10)	117.4(2)	H(21A)-C(21)-H(21B)	109.5
C(14)-C(9)-C(8)	137.0(2)	C(20)-C(21)-H(21C)	109.5
C(10)-C(9)-C(8)	105.6(2)	H(21A)-C(21)-H(21C)	109.5
N(6)-C(10)-C(11)	124.9(2)	H(21B)-C(21)-H(21C)	109.5
N(6)-C(10)-C(9)	109.3(2)	Cl(41)-C(41)-Cl(42)	111.31(14)
C(11)-C(10)-C(9)	125.7(2)	Cl(41)-C(41)-H(41A)	109.4
C(10)-C(11)-C(12)	115.0(2)	Cl(42)-C(41)-H(41A)	109.4
C(10)-C(11)-C(4)	117.59(19)	Cl(41)-C(41)-H(41B)	109.4
C(12)-C(11)-C(4)	127.18(19)	Cl(42)-C(41)-H(41B)	109.4
C(11)-C(12)-C(13)	121.2(2)	H(41A)-C(41)-H(41B)	108.0
C(11)-C(12)-H(12)	119.4		

Anisotropic displacement parameters (Å^2 x 10^3) for **16**. The Anisotropic displacement factor exponent takes the form: $-2\pi^2(h^2 a^{*2} U^{11} + \ldots + 2 h k a^* b^* U^{12})$.

Label	U^{11}	U^{22}	U^{33}	U^{23}	U^{13}	U^{12}
C(1)	12(1)	12(1)	18(1)	-1(1)	2(1)	0(1)
C(2)	15(1)	13(1)	18(1)	0(1)	0(1)	0(1)
N(2)	16(1)	14(1)	18(1)	2(1)	0(1)	1(1)
C(3)	14(1)	12(1)	18(1)	1(1)	1(1)	-2(1)
C(4)	16(1)	13(1)	16(1)	-1(1)	1(1)	0(1)
C(5)	16(1)	13(1)	17(1)	0(1)	2(1)	0(1)
C(6)	25(1)	12(1)	22(1)	0(1)	0(1)	-3(1)
N(6)	21(1)	16(1)	24(1)	-2(1)	1(1)	-6(1)
C(7)	24(1)	18(1)	31(1)	-5(1)	6(1)	-6(1)
C(8)	22(1)	23(1)	34(1)	-10(1)	3(1)	-7(1)
C(9)	17(1)	23(1)	27(1)	-10(1)	2(1)	-2(1)
C(10)	18(1)	18(1)	20(1)	-4(1)	2(1)	-2(1)
C(11)	17(1)	16(1)	18(1)	-3(1)	1(1)	0(1)
C(12)	22(1)	19(1)	21(1)	-3(1)	-1(1)	1(1)
C(13)	27(1)	24(1)	22(1)	-4(1)	-5(1)	6(1)
C(14)	21(1)	31(1)	26(1)	-10(1)	-4(1)	1(1)
C(15)	18(1)	16(1)	32(1)	2(1)	3(1)	6(1)
C(16)	25(1)	21(1)	19(1)	7(1)	0(1)	-1(1)
C(17)	15(1)	13(1)	19(1)	-2(1)	1(1)	-1(1)
N(17)	18(1)	10(1)	23(1)	-1(1)	5(1)	-1(1)
O(17)	22(1)	16(1)	29(1)	-2(1)	9(1)	2(1)
C(18)	18(1)	15(1)	19(1)	-2(1)	5(1)	-2(1)
C(19)	17(1)	26(1)	29(1)	-7(1)	1(1)	1(1)
C(20)	21(1)	11(1)	28(1)	-1(1)	2(1)	-1(1)
C(21)	27(1)	20(1)	39(1)	1(1)	-4(1)	3(1)
I(31)	15(1)	19(1)	25(1)	0(1)	1(1)	-2(1)
C(41)	29(1)	28(1)	30(1)	-2(1)	-1(1)	8(1)
Cl(41)	25(1)	40(1)	24(1)	6(1)	2(1)	8(1)
Cl(42)	40(1)	24(1)	30(1)	2(1)	9(1)	4(1)

Hydrogen coordinates (x 10^4) and isotropic displacement parameters ($Å^2$x 10^3) for **16**.

Label	x	y	z	U_{eq}
H(1)	9172	4886	6619	17
H(2A)	5949	5043	7366	18
H(2B)	8057	4771	7554	18
H(3)	3819	3900	6986	18
H(5)	6806	5098	5818	19
H(6A)	5425	2261	6461	23
H(6B)	4078	2260	7022	23
H(7)	1053	1456	6450	29
H(8)	-827	1876	5584	31
H(12)	4155	5102	5229	25
H(13)	1636	4770	4608	29
H(14)	-332	3493	4758	31
H(15A)	8074	2479	7015	33
H(15B)	9421	3327	7186	33
H(15C)	8502	3280	6554	33
H(16A)	5771	2656	7741	33
H(16B)	4987	3657	7917	33
H(16C)	7153	3397	8035	33
H(18A)	10309	6615	5610	21
H(18B)	10108	5623	5914	21
H(19A)	12585	7053	6313	36
H(19B)	13265	6143	5976	36
H(19C)	12389	6059	6613	36
H(20A)	9921	7963	6293	24
H(20B)	8266	7877	6763	24
H(21A)	7679	7818	5539	43
H(21B)	7138	8676	5945	43
H(21C)	6020	7717	6006	43
H(41A)	1633	10344	5461	35
H(41B)	614	9763	5963	35

Torsion angles (°) for **16**.

Bonds	Angle	Bonds	Angle
C(5)-C(1)-C(2)-N(2)	46.5(2)	C(6)-N(6)-C(10)-C(9)	169.0(2)
C(17)-C(1)-C(2)-N(2)	164.55(16)	C(14)-C(9)-C(10)-N(6)	178.8(2)
C(1)-C(2)-N(2)-C(16)	173.88(17)	C(8)-C(9)-C(10)-N(6)	-0.7(3)
C(1)-C(2)-N(2)-C(15)	55.8(2)	C(14)-C(9)-C(10)-C(11)	0.2(3)
C(1)-C(2)-N(2)-C(3)	-68.2(2)	C(8)-C(9)-C(10)-C(11)	-179.3(2)
C(16)-N(2)-C(3)-C(4)	166.76(17)	N(6)-C(10)-C(11)-C(12)	-178.6(2)
C(15)-N(2)-C(3)-C(4)	-72.2(2)	C(9)-C(10)-C(11)-C(12)	-0.2(3)
C(2)-N(2)-C(3)-C(4)	50.2(2)	N(6)-C(10)-C(11)-C(4)	-3.8(3)
C(16)-N(2)-C(3)-C(6)	-66.5(2)	C(9)-C(10)-C(11)-C(4)	174.6(2)
C(15)-N(2)-C(3)-C(6)	54.6(2)	C(5)-C(4)-C(11)-C(10)	172.5(2)
C(2)-N(2)-C(3)-C(6)	176.89(16)	C(3)-C(4)-C(11)-C(10)	-13.0(3)
N(2)-C(3)-C(4)-C(5)	-15.8(3)	C(5)-C(4)-C(11)-C(12)	-13.5(3)
C(6)-C(3)-C(4)-C(5)	-141.7(2)	C(3)-C(4)-C(11)-C(12)	161.0(2)
N(2)-C(3)-C(4)-C(11)	169.74(16)	C(10)-C(11)-C(12)-C(13)	-0.2(3)
C(6)-C(3)-C(4)-C(11)	43.8(2)	C(4)-C(11)-C(12)-C(13)	-174.3(2)
C(11)-C(4)-C(5)-C(1)	168.57(18)	C(11)-C(12)-C(13)-C(14)	0.5(4)
C(3)-C(4)-C(5)-C(1)	-5.4(3)	C(12)-C(13)-C(14)-C(9)	-0.5(4)
C(2)-C(1)-C(5)-C(4)	-10.0(3)	C(10)-C(9)-C(14)-C(13)	0.2(3)
C(17)-C(1)-C(5)-C(4)	-128.0(2)	C(8)-C(9)-C(14)-C(13)	179.5(3)
N(2)-C(3)-C(6)-N(6)	178.57(16)	C(5)-C(1)-C(17)-O(17)	95.5(2)
C(4)-C(3)-C(6)-N(6)	-55.9(2)	C(2)-C(1)-C(17)-O(17)	-23.9(3)
C(3)-C(6)-N(6)-C(10)	40.8(3)	C(5)-C(1)-C(17)-N(17)	-80.4(2)
C(3)-C(6)-N(6)-C(7)	-154.2(2)	C(2)-C(1)-C(17)-N(17)	160.14(18)
C(10)-N(6)-C(7)-C(8)	-0.9(3)	O(17)-C(17)-N(17)-C(18)	174.7(2)
C(6)-N(6)-C(7)-C(8)	-167.3(2)	C(1)-C(17)-N(17)-C(18)	-9.5(3)
N(6)-C(7)-C(8)-C(9)	0.4(3)	O(17)-C(17)-N(17)-C(20)	-1.8(3)
C(7)-C(8)-C(9)-C(14)	-179.2(3)	C(1)-C(17)-N(17)-C(20)	173.96(19)
C(7)-C(8)-C(9)-C(10)	0.2(3)	C(17)-N(17)-C(18)-C(19)	-99.0(3)
C(7)-N(6)-C(10)-C(11)	179.6(2)	C(20)-N(17)-C(18)-C(19)	77.6(2)
C(6)-N(6)-C(10)-C(11)	-12.4(3)	C(17)-N(17)-C(20)-C(21)	-92.1(2)
C(7)-N(6)-C(10)-C(9)	1.0(3)	C(18)-N(17)-C(20)-C(21)	91.0(2)

Hydrogen bonds for **16** (Å and °).

D-H...A	d(D-H)	d(H...A)	d(D...A)	<(DHA)
C(2)-H(2A)...I(31)#1	0.99	3.15	4.062(2)	153
C(2)-H(2B)...I(31)#2	0.99	2.93	3.825(2)	151
C(3)-H(3)...I(31)#1	1.00	3.18	4.109(2)	155
C(6)-H(6B)...O(17)#3	0.99	2.40	3.391(3)	177
C(7)-H(7)...I(31)#4	0.95	3.11	3.948(2)	148
C(16)-H(16A)...O(17)#3	0.98	2.40	3.245(3)	144
C(16)-H(16B)...I(31)#1	0.98	3.11	4.031(2)	157
C(20)-H(20A)...Cl(42)#5	0.99	2.96	3.775(2)	140
C(41)-H(41B)...I(31)#6	0.99	3.17	4.081(3)	154

Symmetry transformations used to generate equivalent atoms:
#1 -x+1,y+1/2,-z+3/2 #2 -x+2,y+1/2,-z+3/2 #3 -x+1,y-1/2,-z+3/2 #4 x-1,y,z #5 x+1,y,z #6 x-1,y+1,z

Structural determination and Refinement:

All crystallographic calculations were performed on a Surface Pro7 with Intel i7-1065G7 at 1.30GHz with four cores, eight processors and 16GB of extended memory. Data collected were corrected for Lorentz and polarization effects with Saint *(39)* and absorption using Blessing's method and merged as incorporated with the program Sadabs (*40,41*). The SHELXTL (*42*) program package was implemented to determine the probable space group and set up the initial files. System symmetry, systematic absences and intensity statistics indicated the non-centrosymmetric orthorhombic space group $P2_12_12_1$ (no. 19). The structure was determined by direct methods with the molecule being located using the program XT (*43*). The structure was refined with XL (*44*). The 48297 data collected were merged based upon identical indices to 28854, then merged for least squares refinement to 7321 unique data [R(int)=0.0163]. All non-hydrogen atoms were refined anisotropically. Hydrogen atoms were idealized throughout the final refinement stages. The final structure was refined to convergence with R(F)=1.89%, wR(F^2)=4.96%, GOF=1.081 for all 7321 unique reflections [R(F)=1.84%, wR(F^2)=4.93% for those 7222 data with Fo > 4σ(Fo)]. The final difference-Fourier map was featureless indicating that the structure is both correct and complete. An empirical correction for extinction was also attempted and found to be negative and therefore not applied. The structure's absolute structure parameters were determined to be: Flack(x) (*45*), -0.014(3); Hooft(y) (*46*), -0.013(2) and the Parsons(z) *(44)*, -0.013(3) indicating that the structure's absolute configuration has been determined reliably *(47)*; these values would be close to 1.0 if the structure were inverted.

^{1}H and ^{13}C NMR Spectra

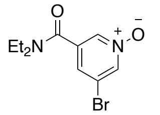
O
Et2N
N
O
Br

3

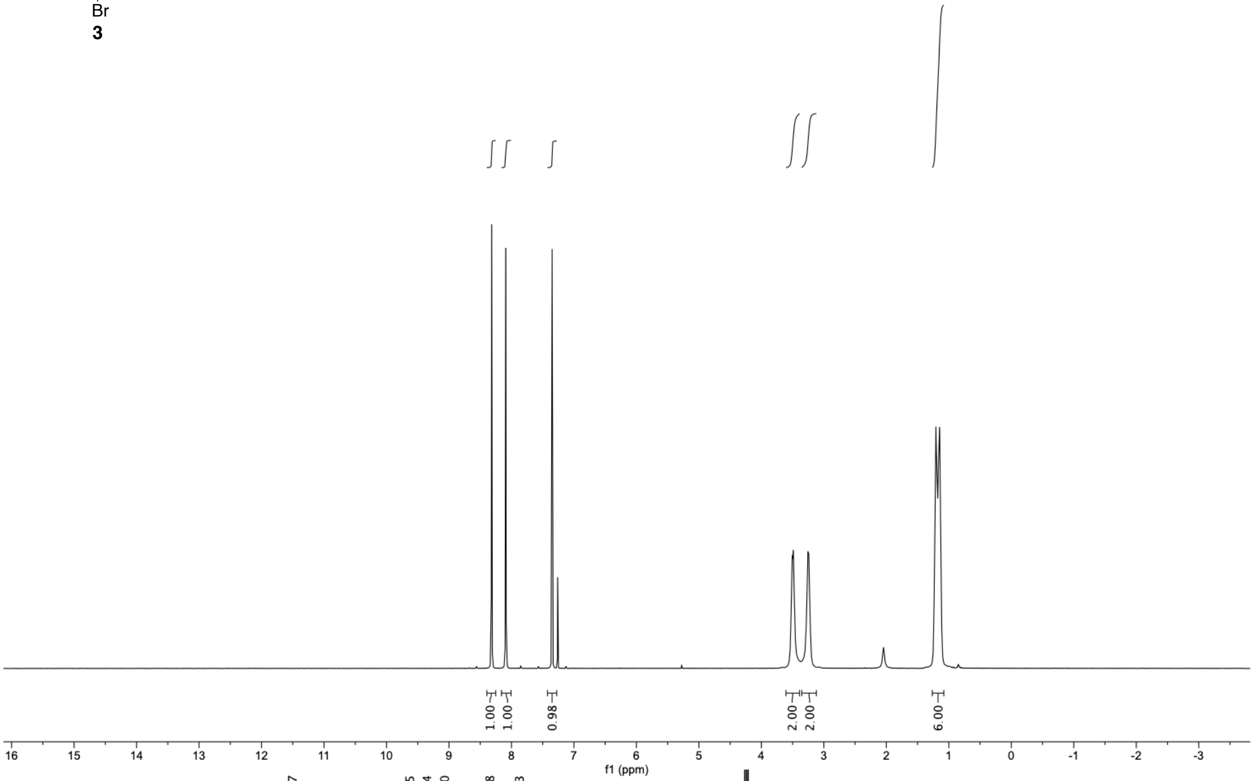
1.00
1.00
0.98
2.00
2.00
6.00
16 15 14 13 12 11 10 9 8 7 6 5 4 3 2 1 0 -1 -2 -3
f1 (ppm)

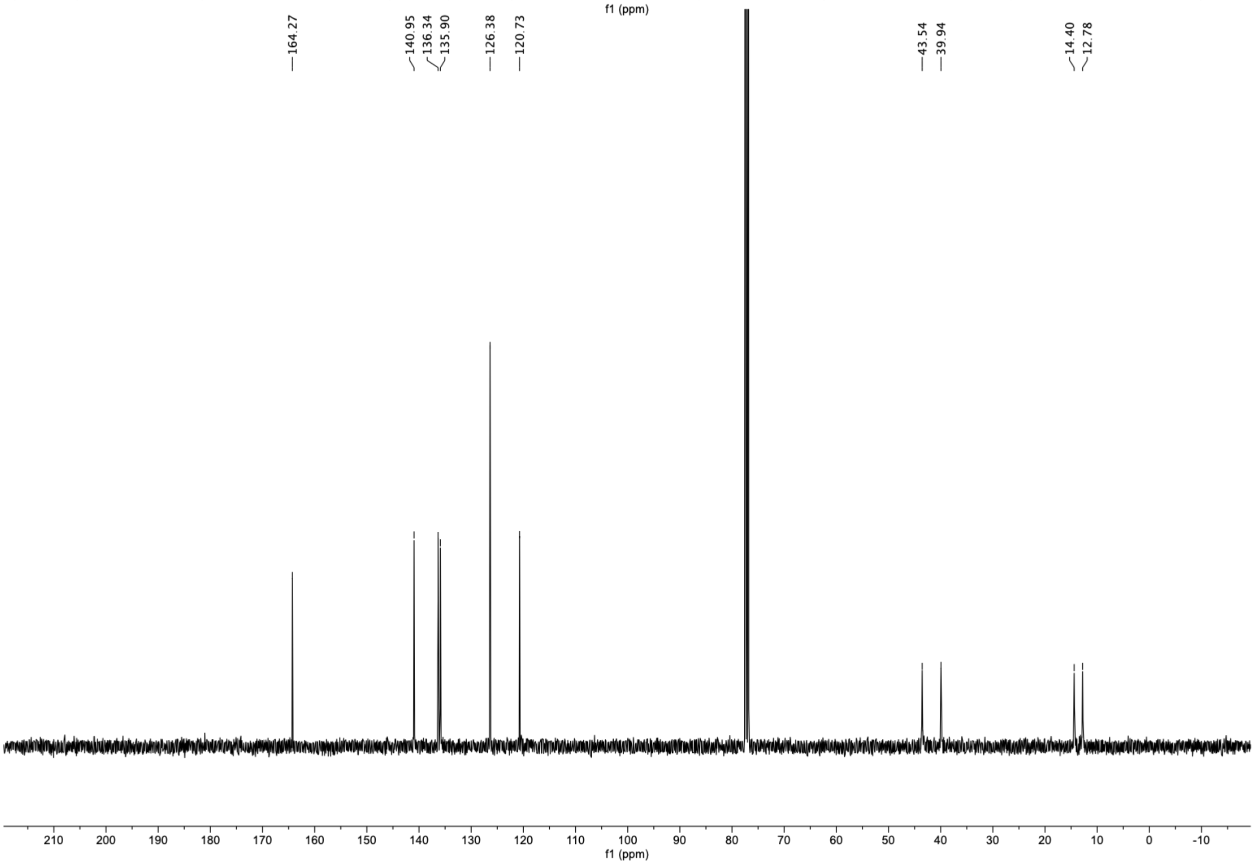
164.27
140.95
136.34
135.90
126.38
120.73
43.54
39.94
14.40
12.78
210 200 190 180 170 160 150 140 130 120 110 100 90 80 70 60 50 40 30 20 10 0 -10
f1 (ppm)

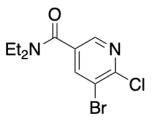
O
Et2N
N
Cl
Br

4

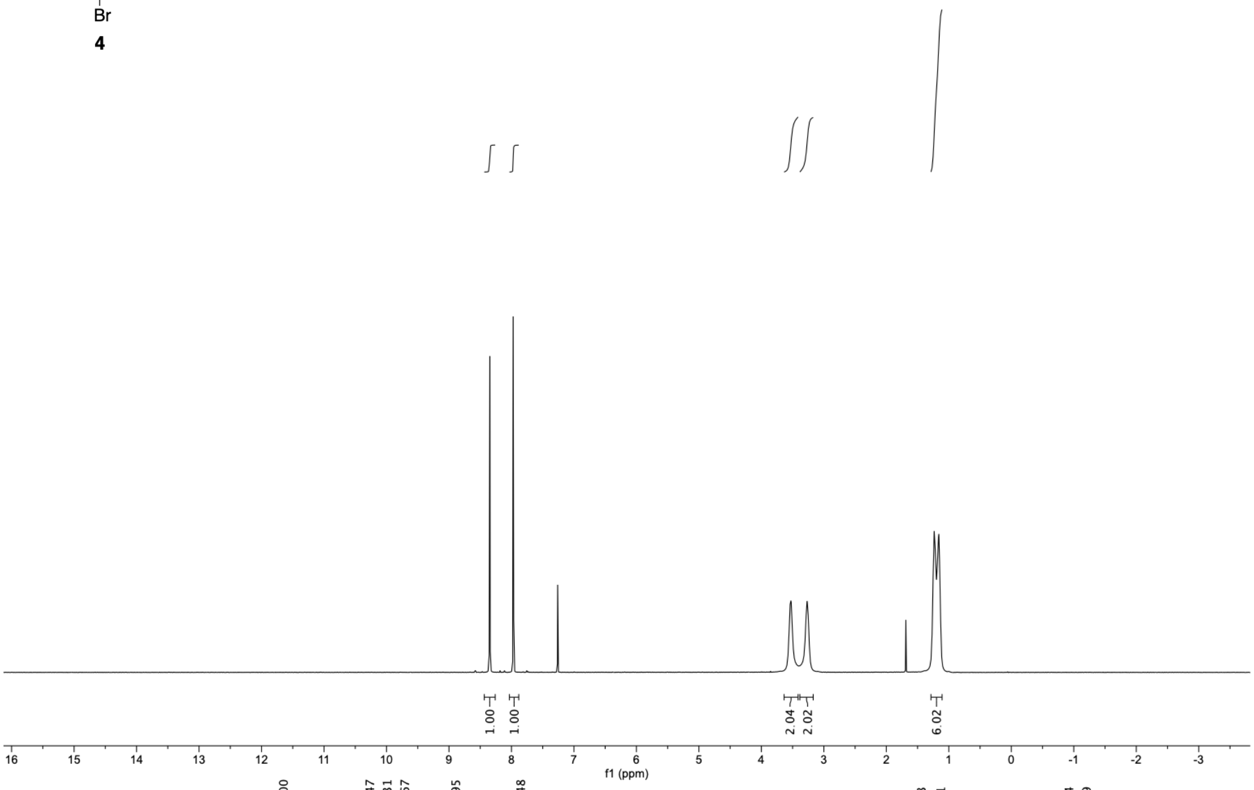
1.00
1.00
2.04
2.02
6.02
f1 (ppm)

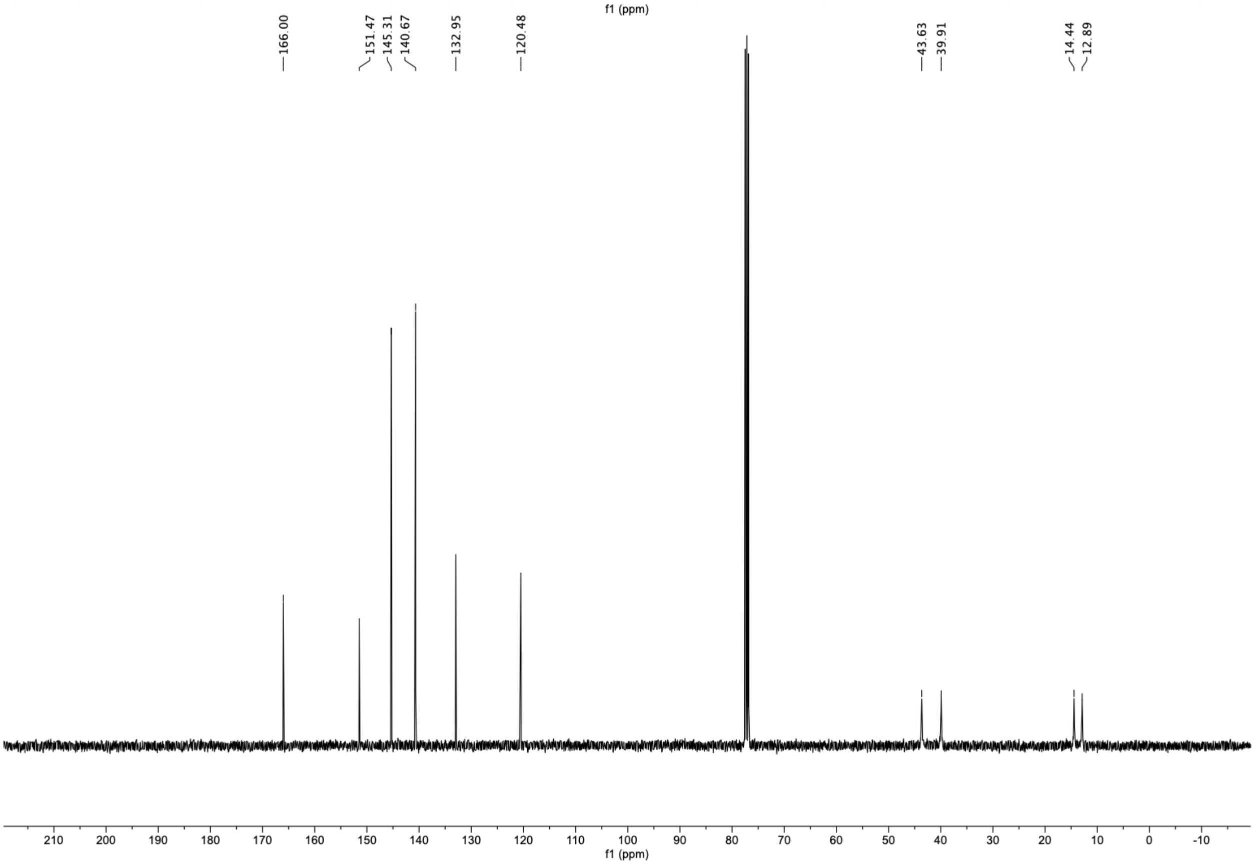
166.00
151.47
145.31
140.67
132.95
120.48
43.63
39.91
14.44
12.89
f1 (ppm)

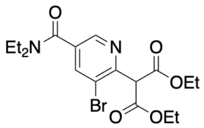

6

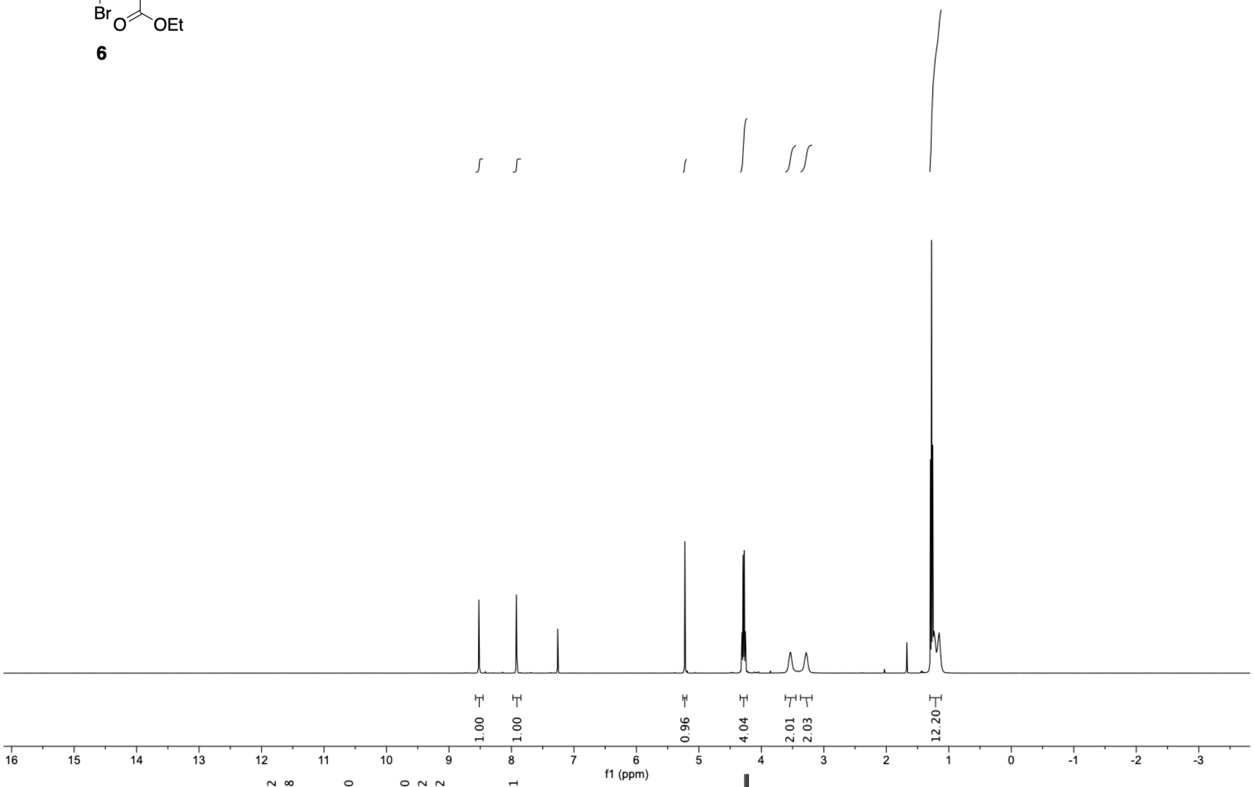

1.00
1.00
0.96
4.04
2.01
2.03
12.20
f1 (ppm)

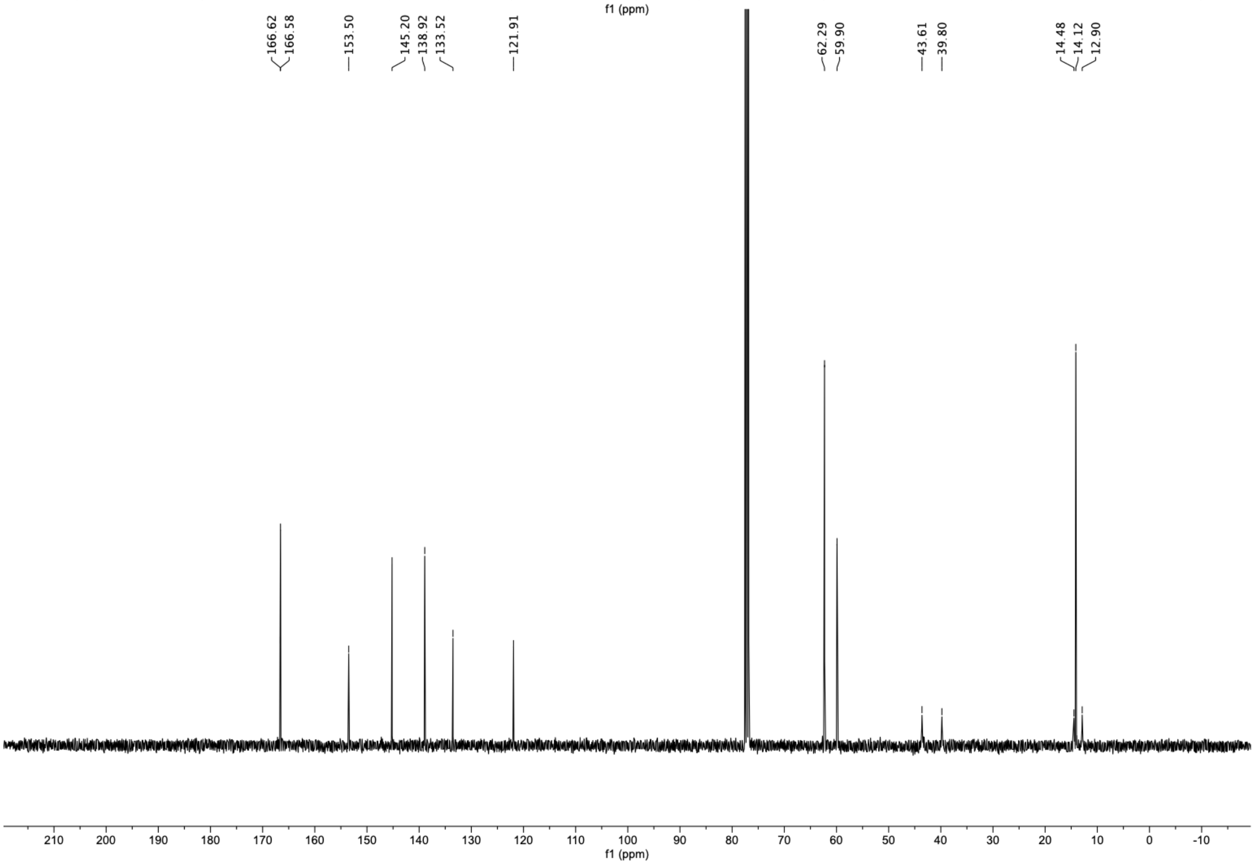

166.62
166.58
153.50
145.20
138.92
133.52
121.91
62.29
59.90
43.61
39.80
14.48
14.12
12.90
f1 (ppm)

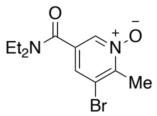

O
Et_2N
N
O
Me
Br

8

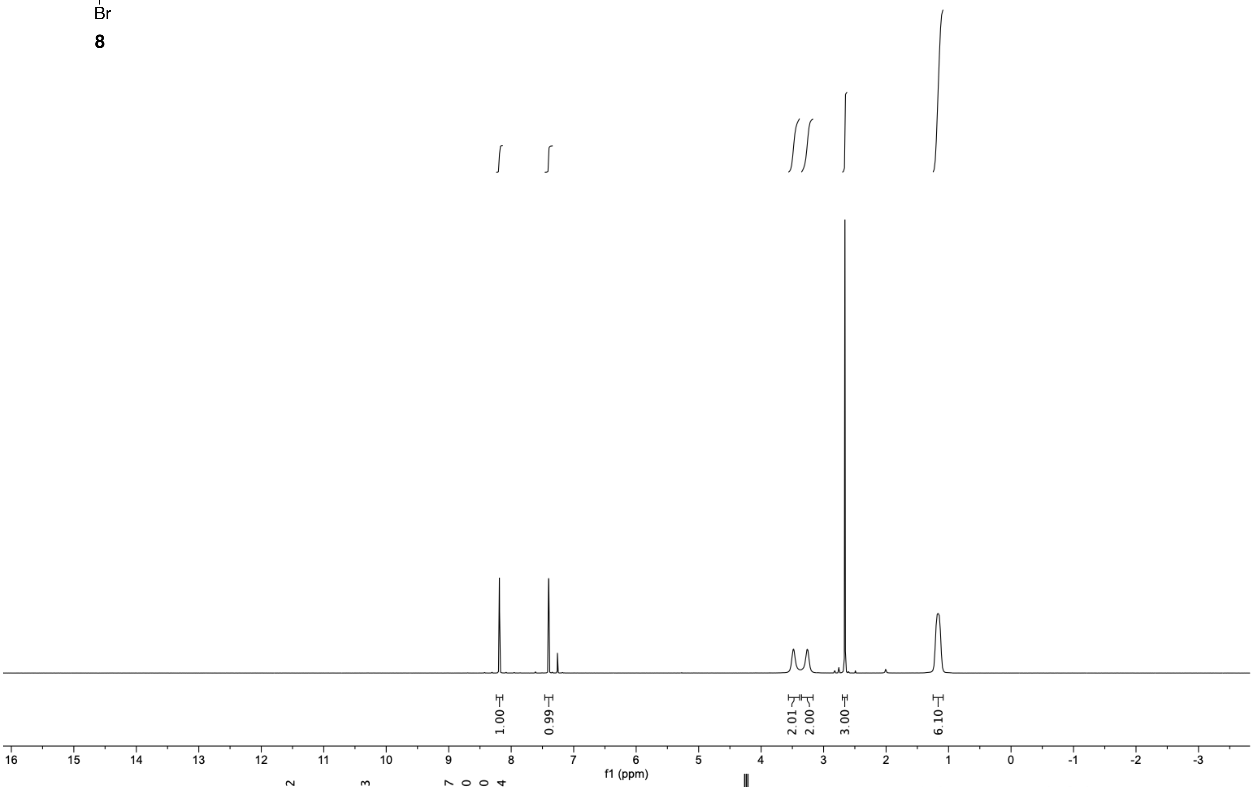

1.00
0.99
2.01
2.00
3.00
6.10
16 15 14 13 12 11 10 9 8 7 6 5 4 3 2 1 0 -1 -2 -3
f1 (ppm)

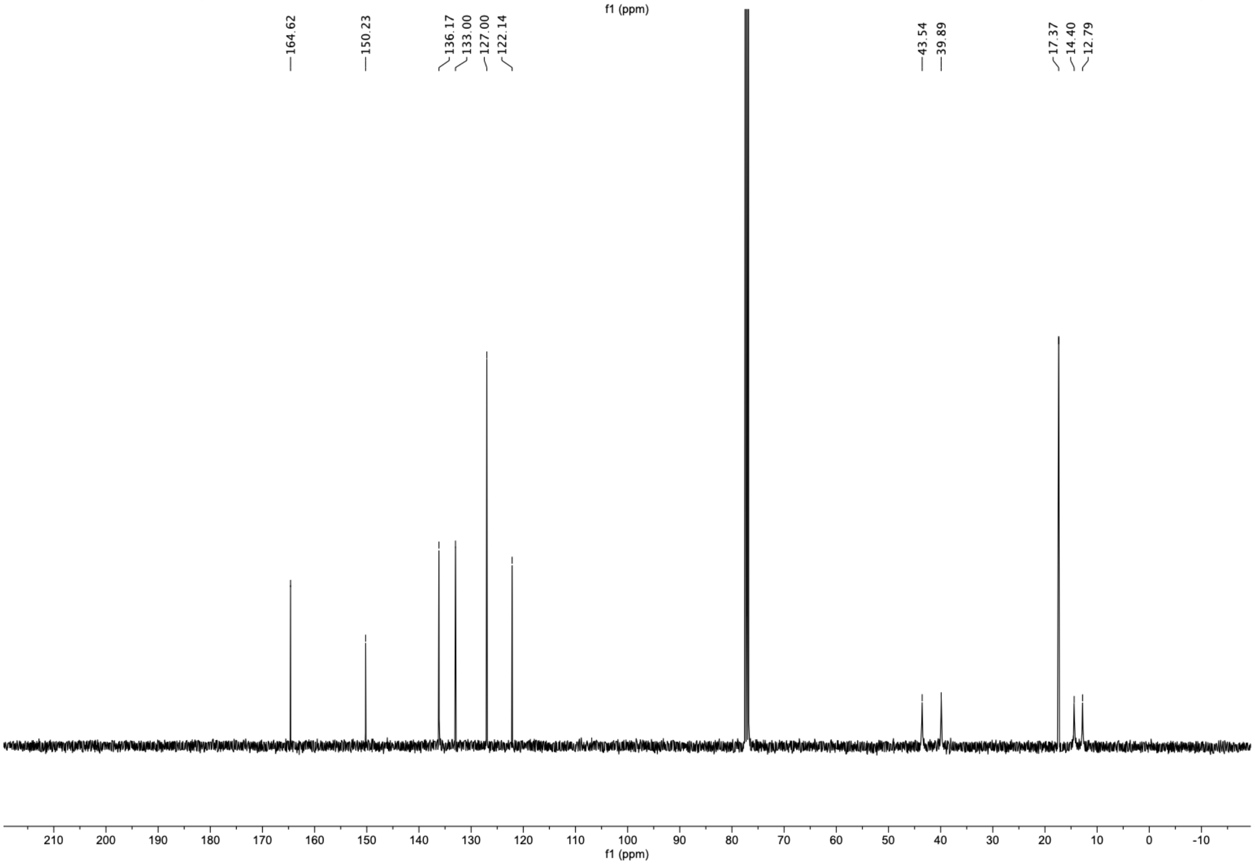

164.62
150.23
136.17
133.00
127.00
122.14
43.54
39.89
17.37
14.40
12.79
210 200 190 180 170 160 150 140 130 120 110 100 90 80 70 60 50 40 30 20 10 0 -10
f1 (ppm)

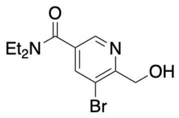
O
Et2N
N
OH
Br

9

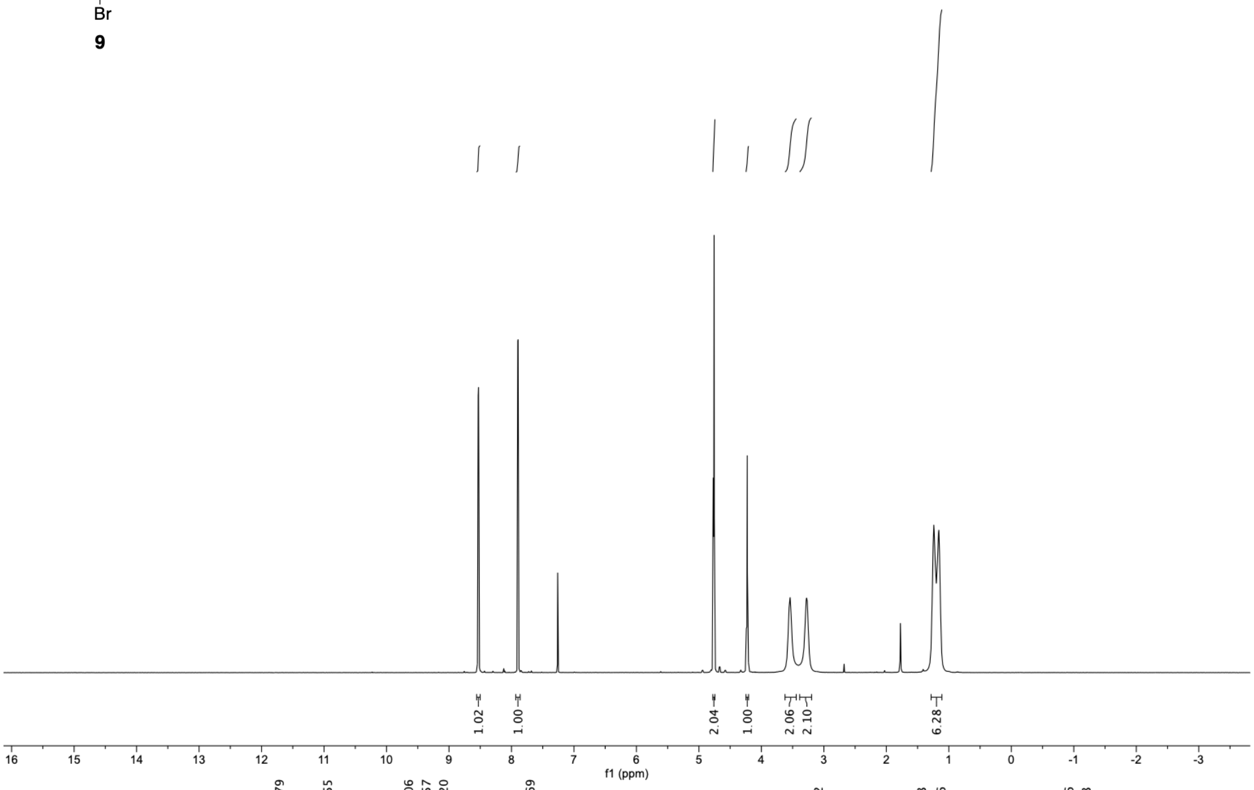
1.02
1.00
2.04
1.00
2.06
2.10
6.28
f1 (ppm)

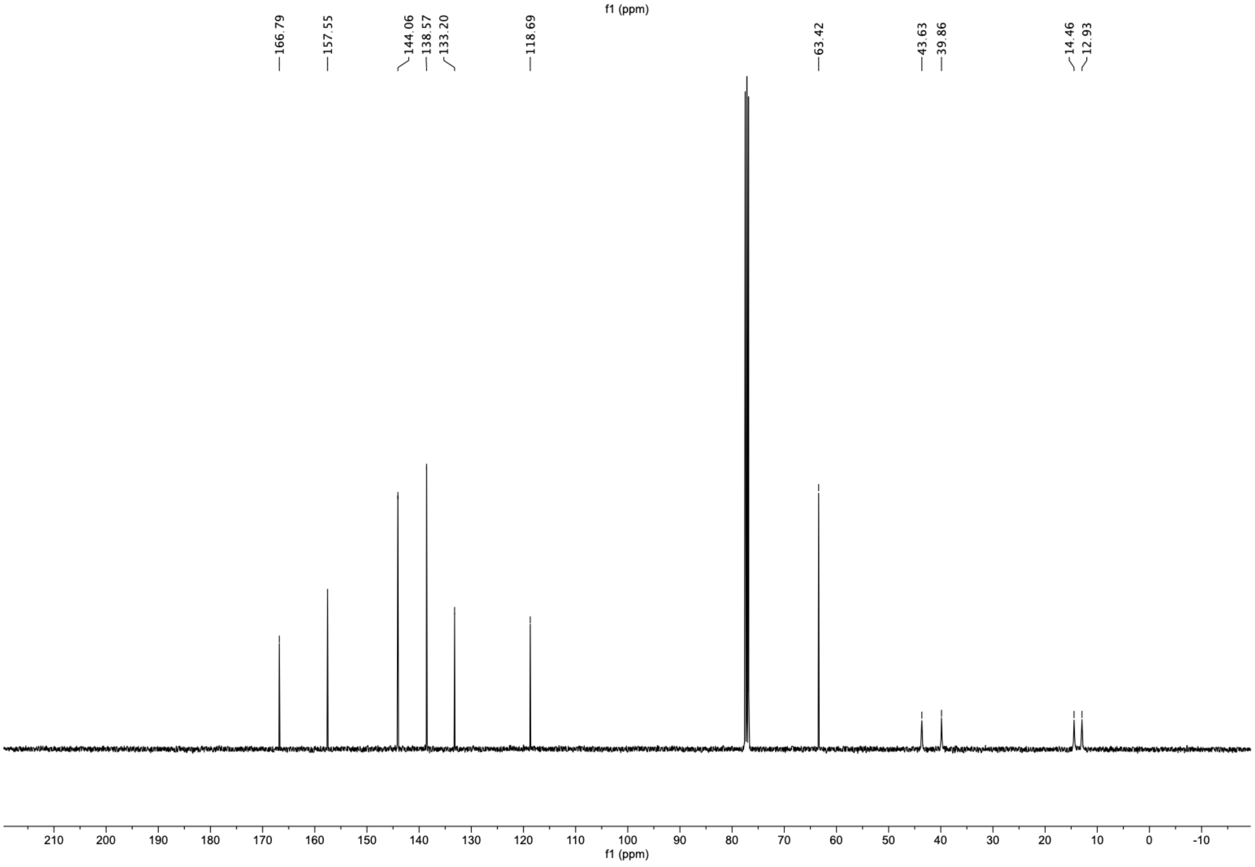
166.79
157.55
144.06
138.57
133.20
118.69
63.42
43.63
39.86
14.46
12.93
f1 (ppm)

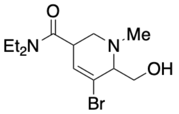

10 (major), 11 (minor)
[5:2 dr; inseparable]

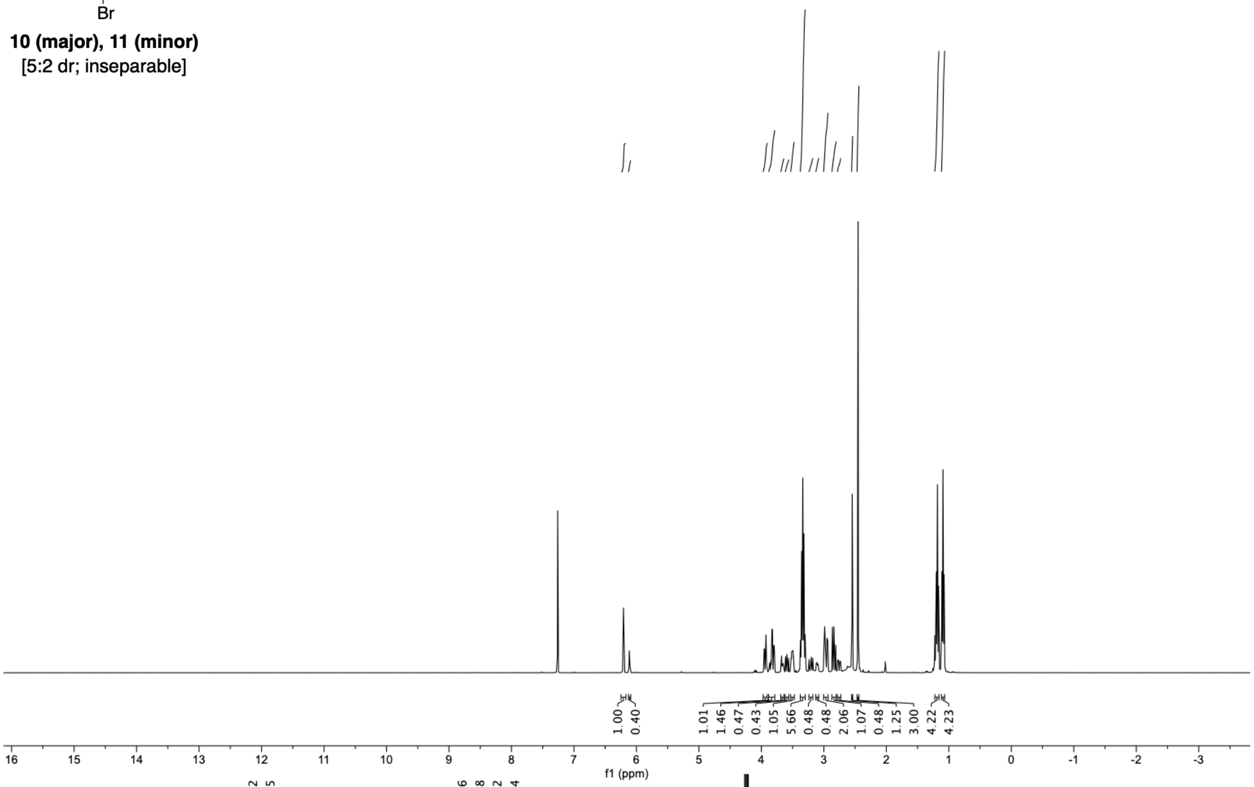

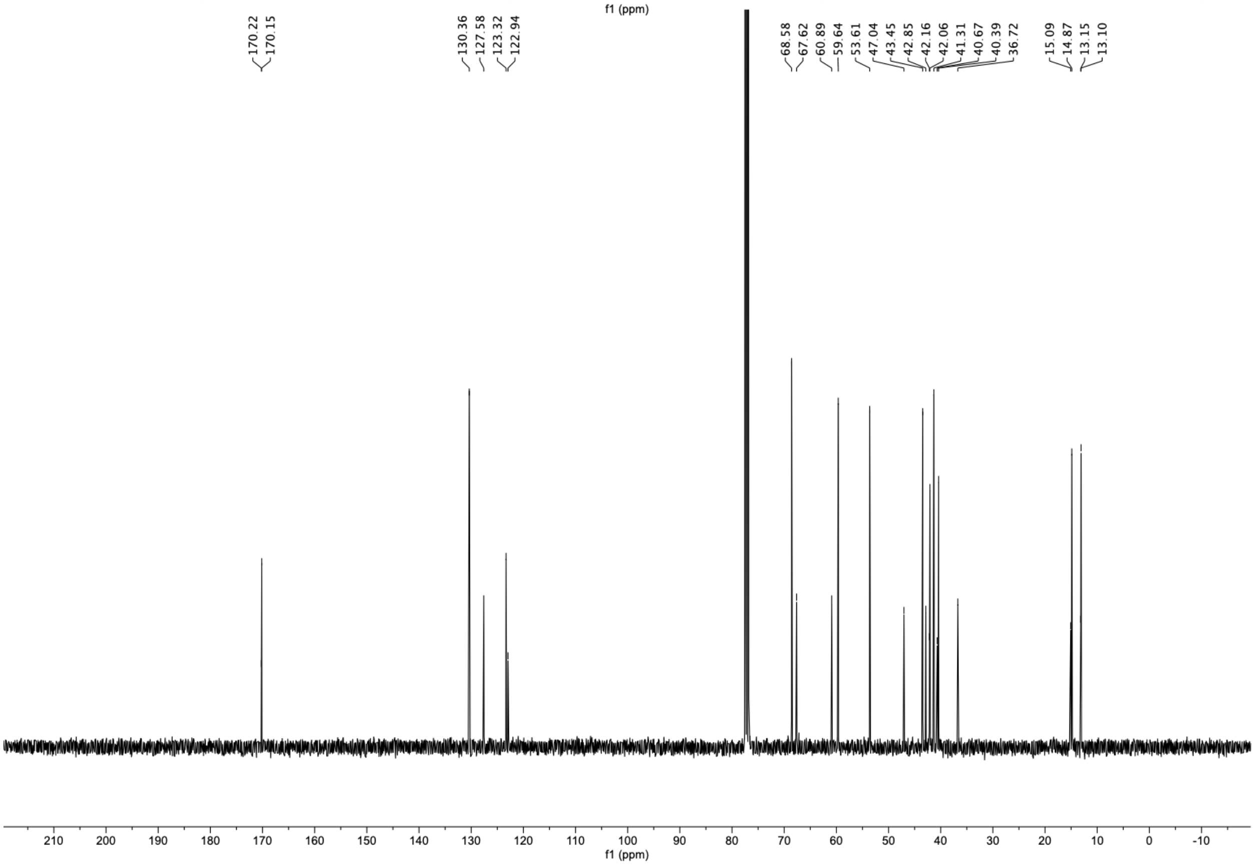

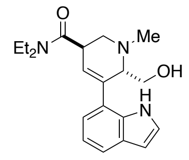
O
Et2N
N
Me
OH
H
N

12

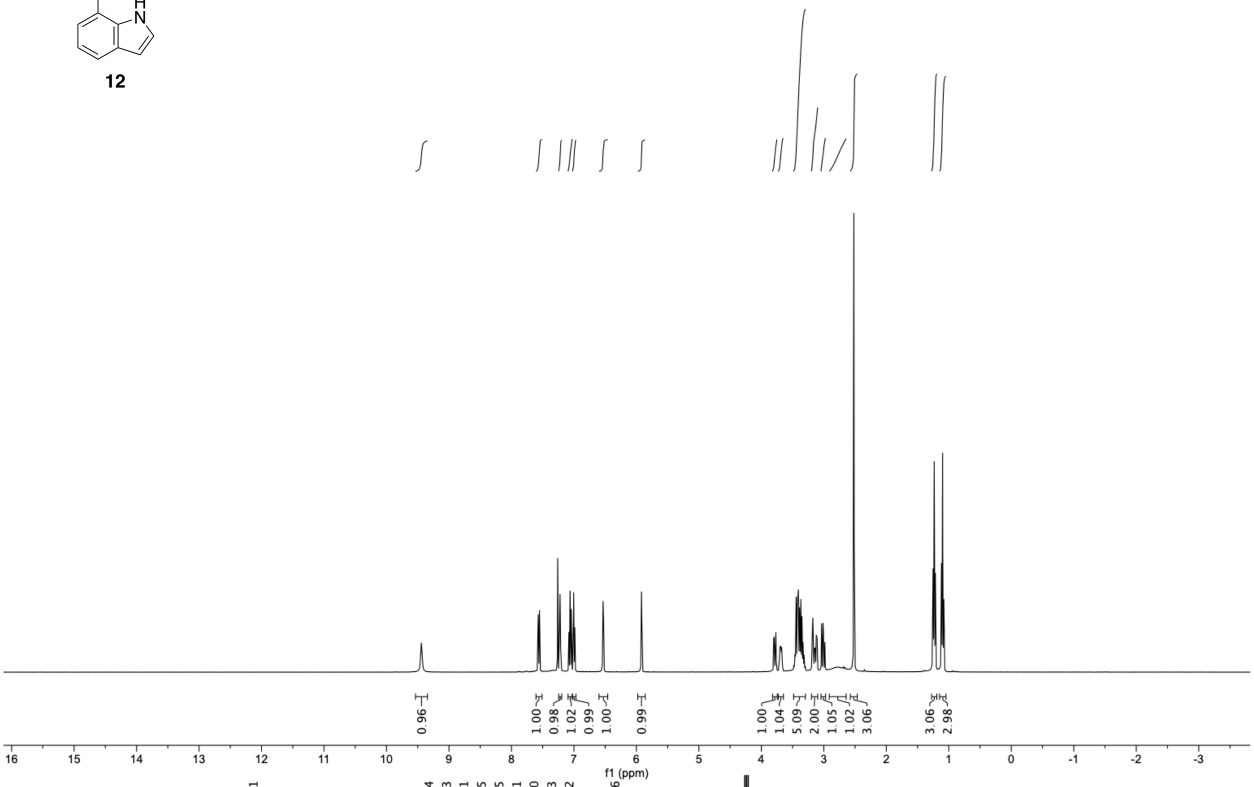
0.96
1.00
0.98
1.02
0.99
1.00
0.99
1.00
1.04
5.09
2.00
1.05
1.02
3.06
3.06
2.98
f1 (ppm)

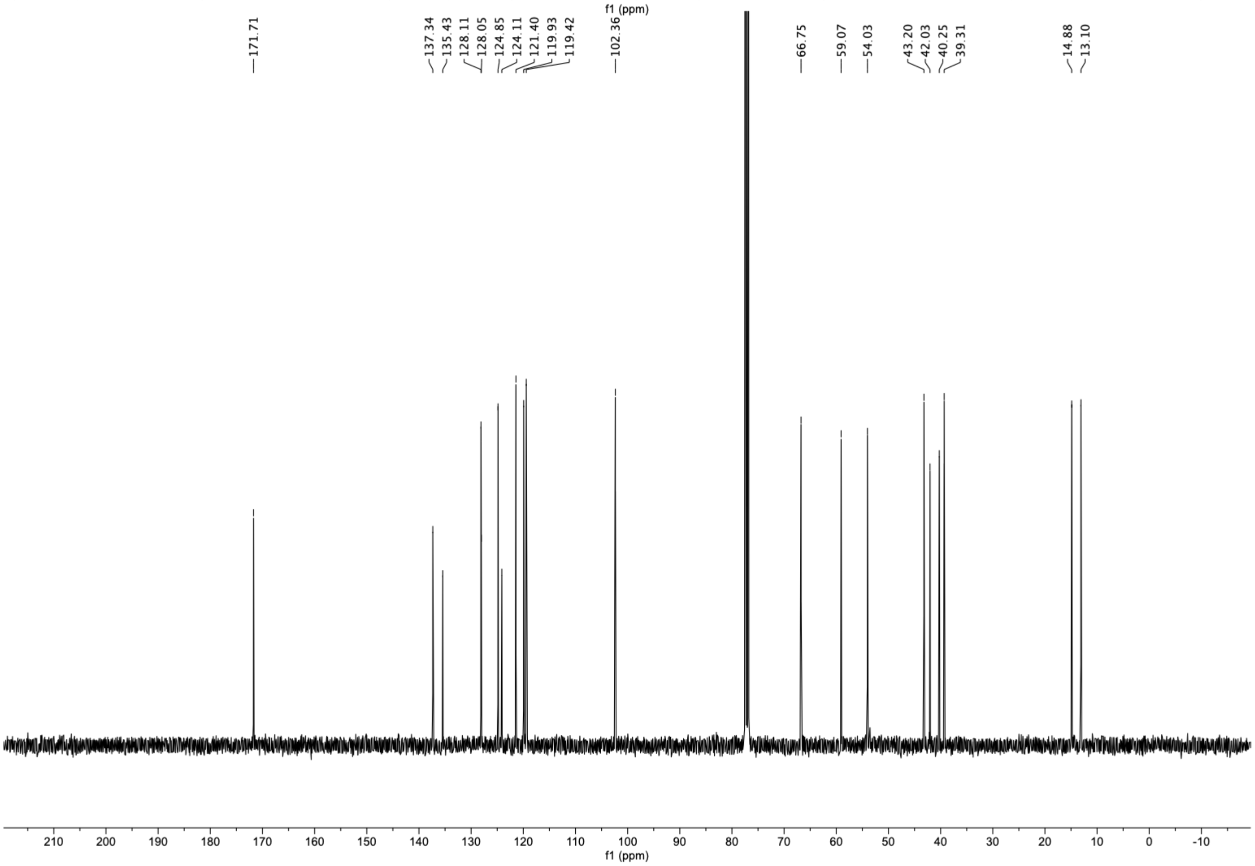
171.71
137.34
135.43
128.11
128.05
124.85
124.11
121.40
119.93
119.42
102.36
66.75
59.07
54.03
43.20
42.03
40.25
39.31
14.88
13.10
f1 (ppm)

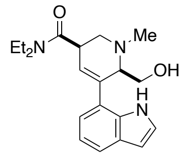
Et2N
O
N
Me
OH
H
N

13

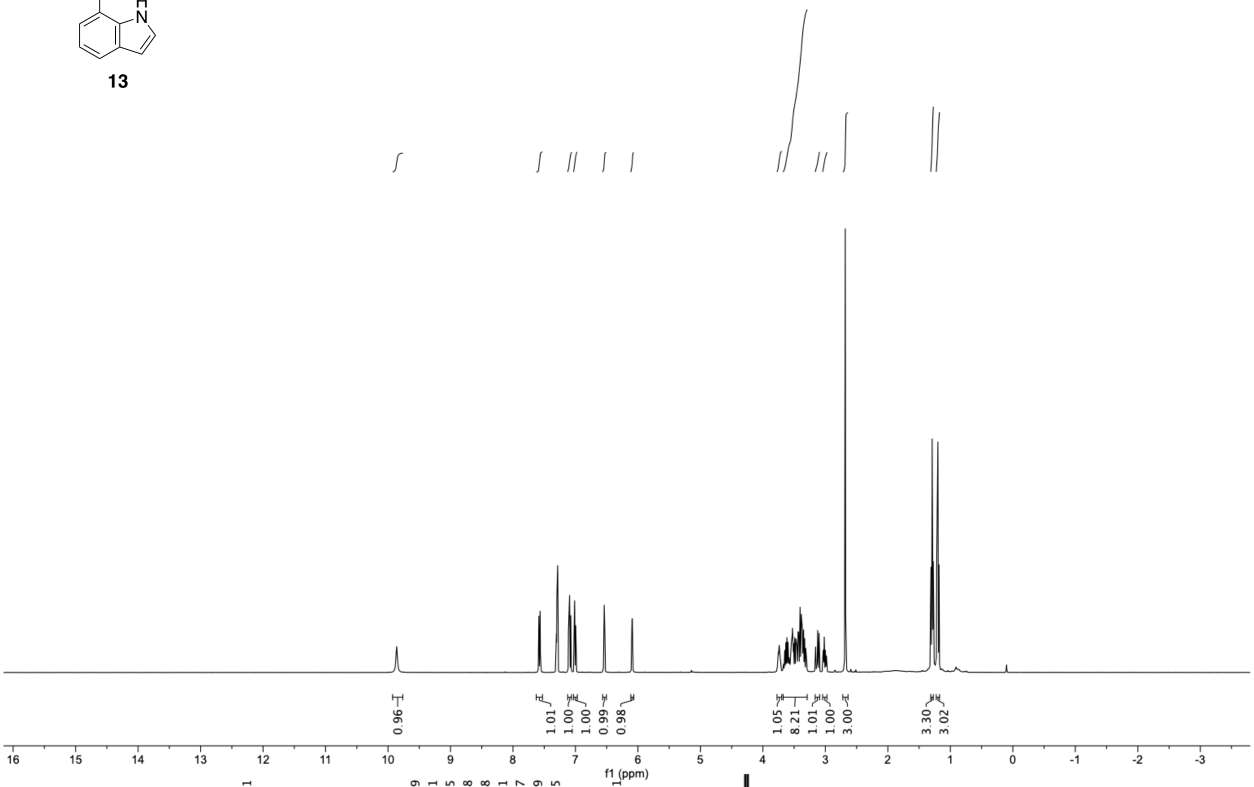
0.96
1.01
1.00
1.00
0.99
0.98
1.05
8.21
1.01
1.00
3.00
3.30
3.02
f1 (ppm)

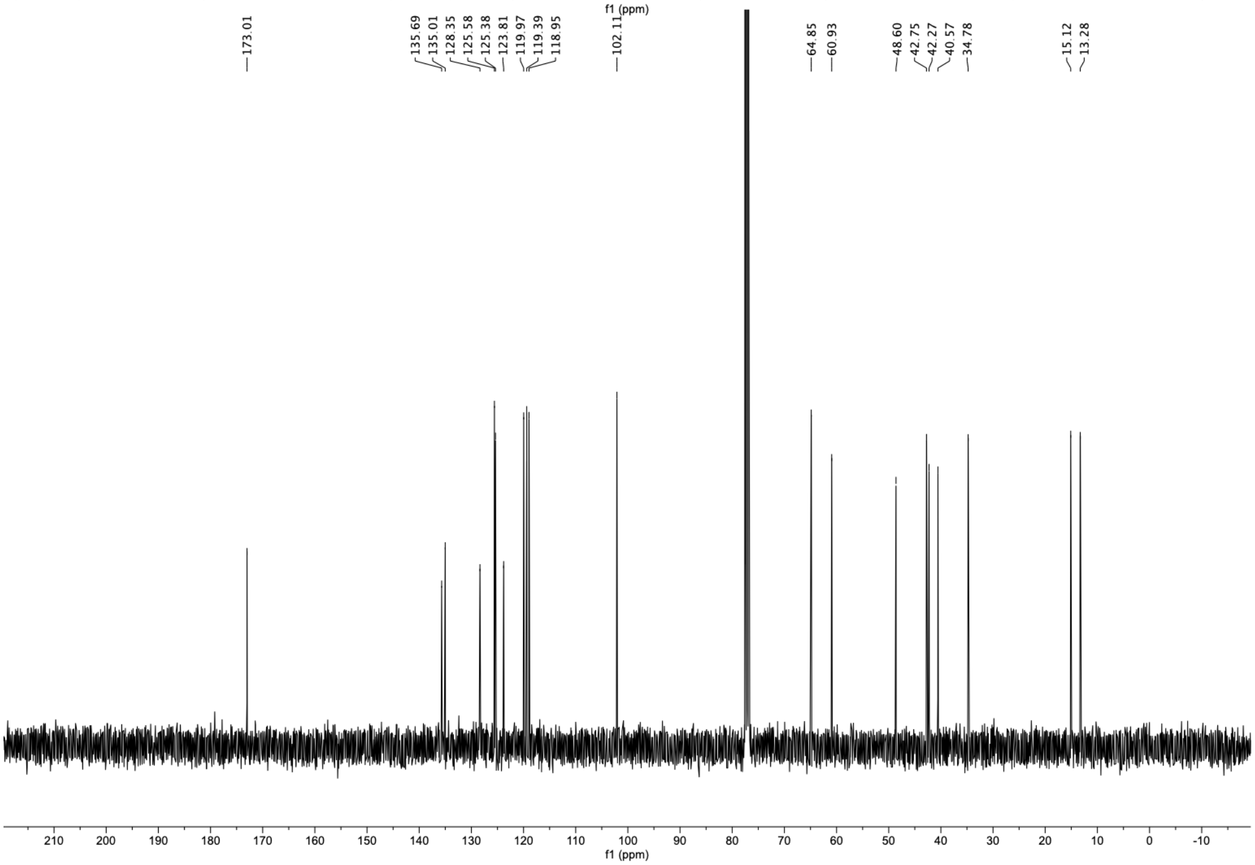
173.01
135.69
135.01
128.35
125.58
125.38
123.81
119.97
119.39
118.95
102.11
64.85
60.93
48.60
42.75
42.27
40.57
34.78
15.12
13.28
f1 (ppm)

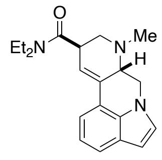
O
Et2N
N
Me
H
N

14

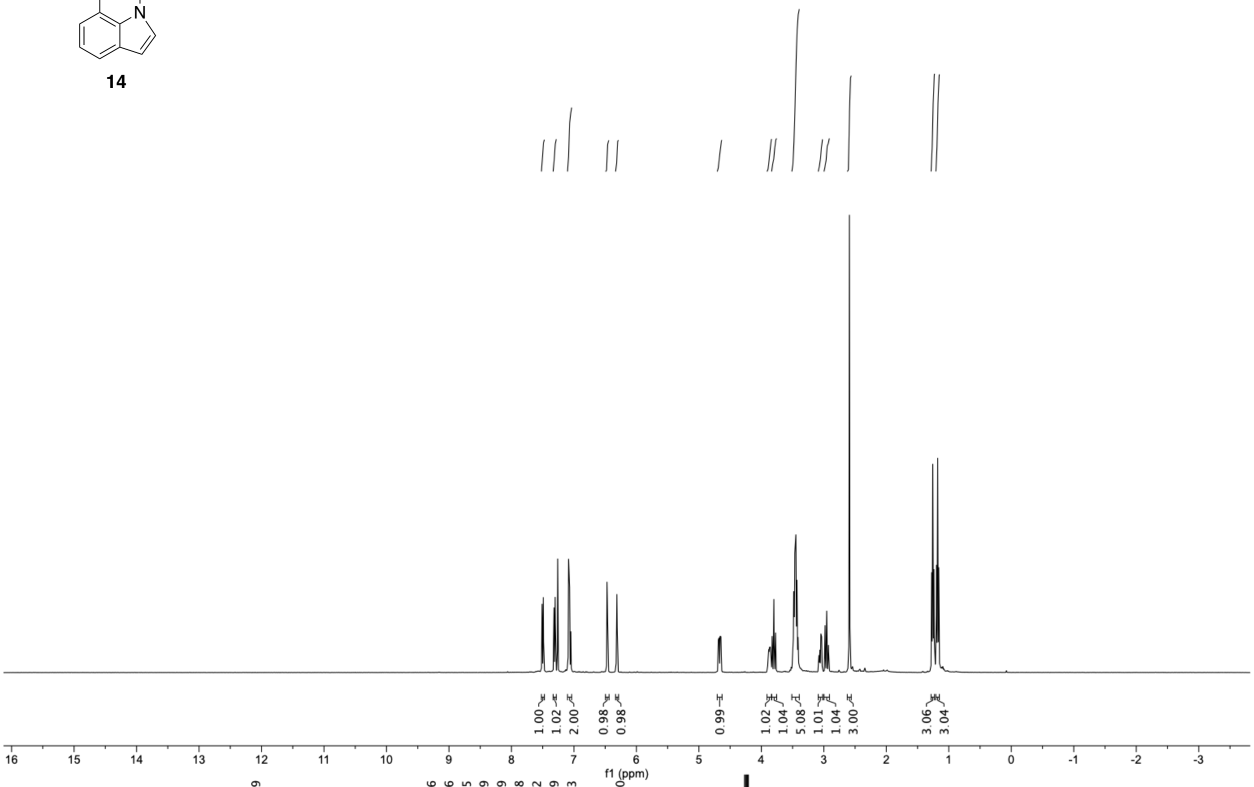
1.00
1.02
2.00
0.98
0.98
0.99
1.02
1.04
5.08
1.01
1.04
3.00
3.06
3.04
16 15 14 13 12 11 10 9 8 7 6 5 4 3 2 1 0 -1 -2 -3
f1 (ppm)

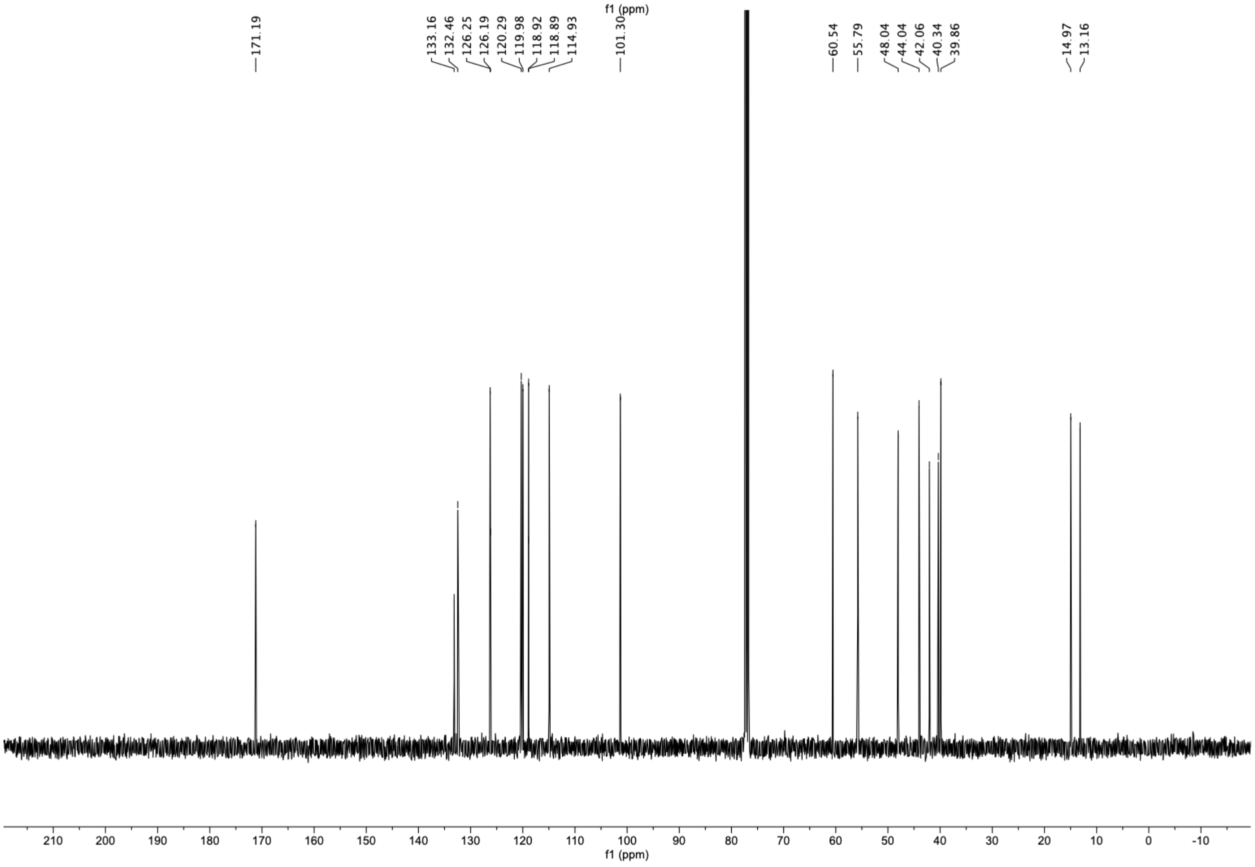
171.19
133.16
132.46
126.25
126.19
120.29
119.98
118.92
118.89
114.93
101.30
60.54
55.79
48.04
44.04
42.06
40.34
39.86
14.97
13.16
210 200 190 180 170 160 150 140 130 120 110 100 90 80 70 60 50 40 30 20 10 0 -10
f1 (ppm)

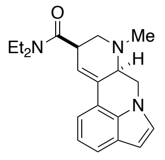
O
Et_2N
N
Me
H
N

15

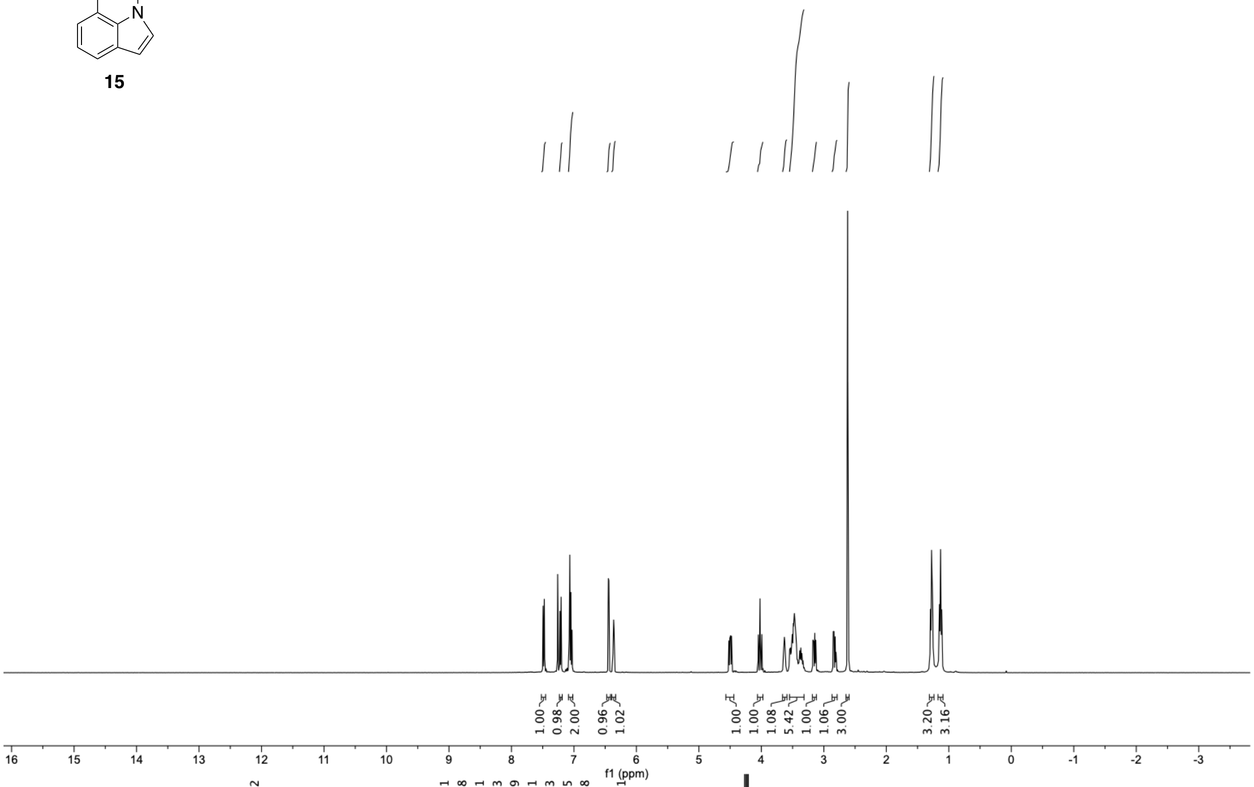
1.00
0.98
2.00
0.96
1.02
1.00
1.00
1.08
5.42
1.00
1.06
3.00
3.20
3.16
16
15
14
13
12
11
10
9
8
7
6
5
4
3
2
1
0
-1
-2
-3
f1 (ppm)

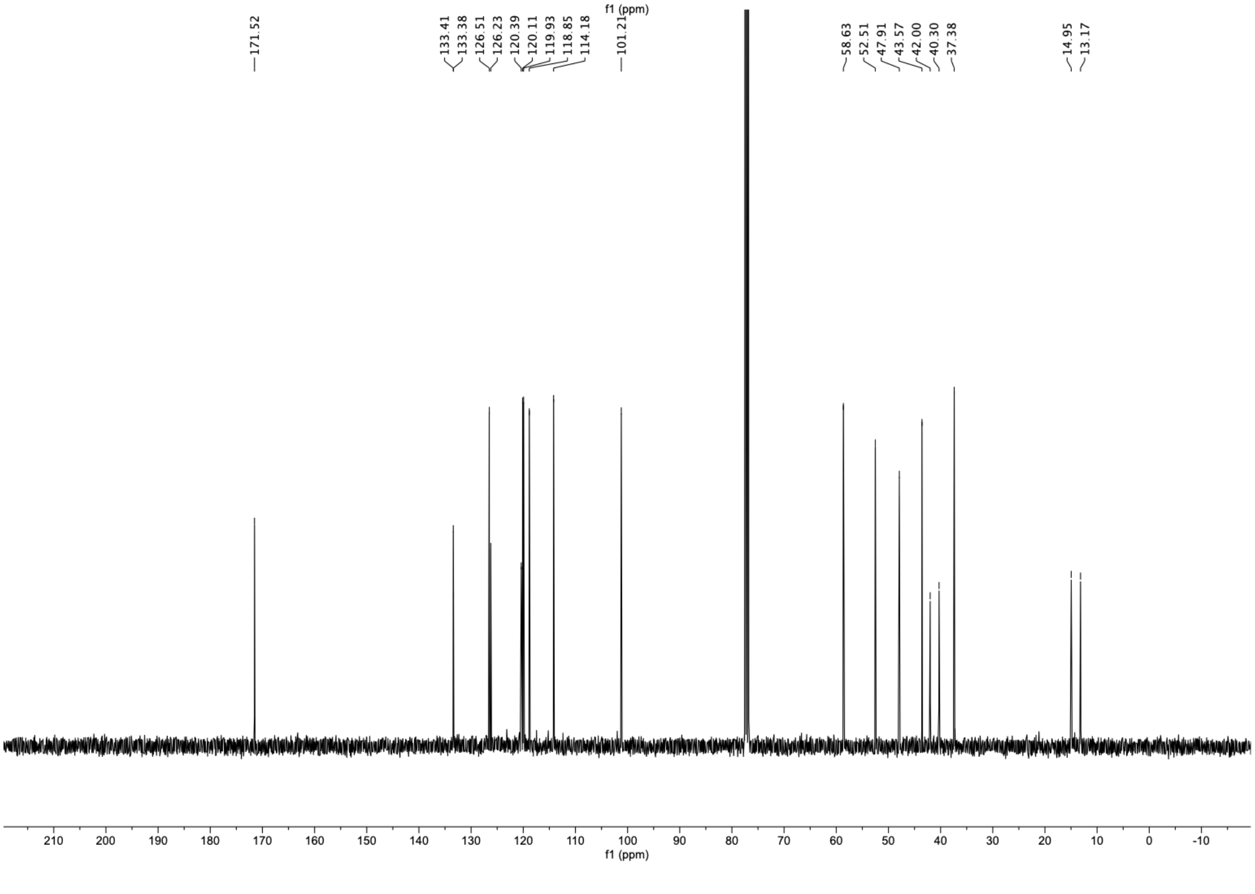
171.52
133.41
133.38
126.51
126.23
120.39
120.11
119.93
118.85
114.18
101.21
58.63
52.51
47.91
43.57
42.00
40.30
37.38
14.95
13.17
210
200
190
180
170
160
150
140
130
120
110
100
90
80
70
60
50
40
30
20
10
0
-10
f1 (ppm)

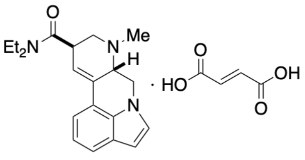

(±)-JRT · Fumarate

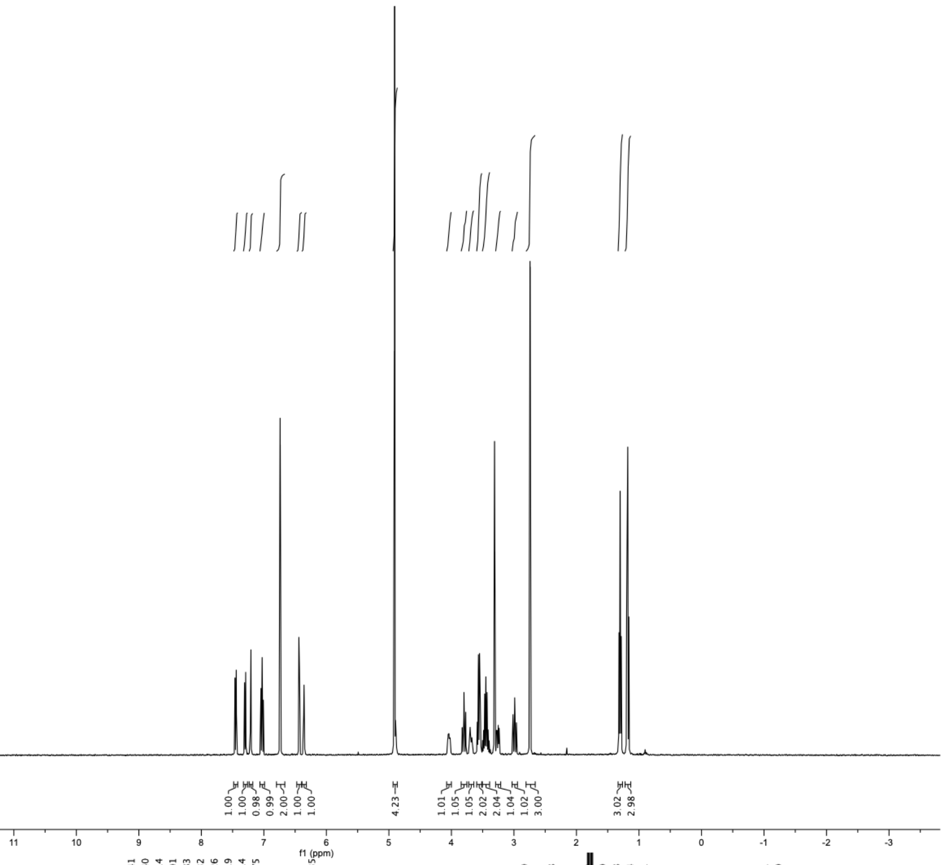

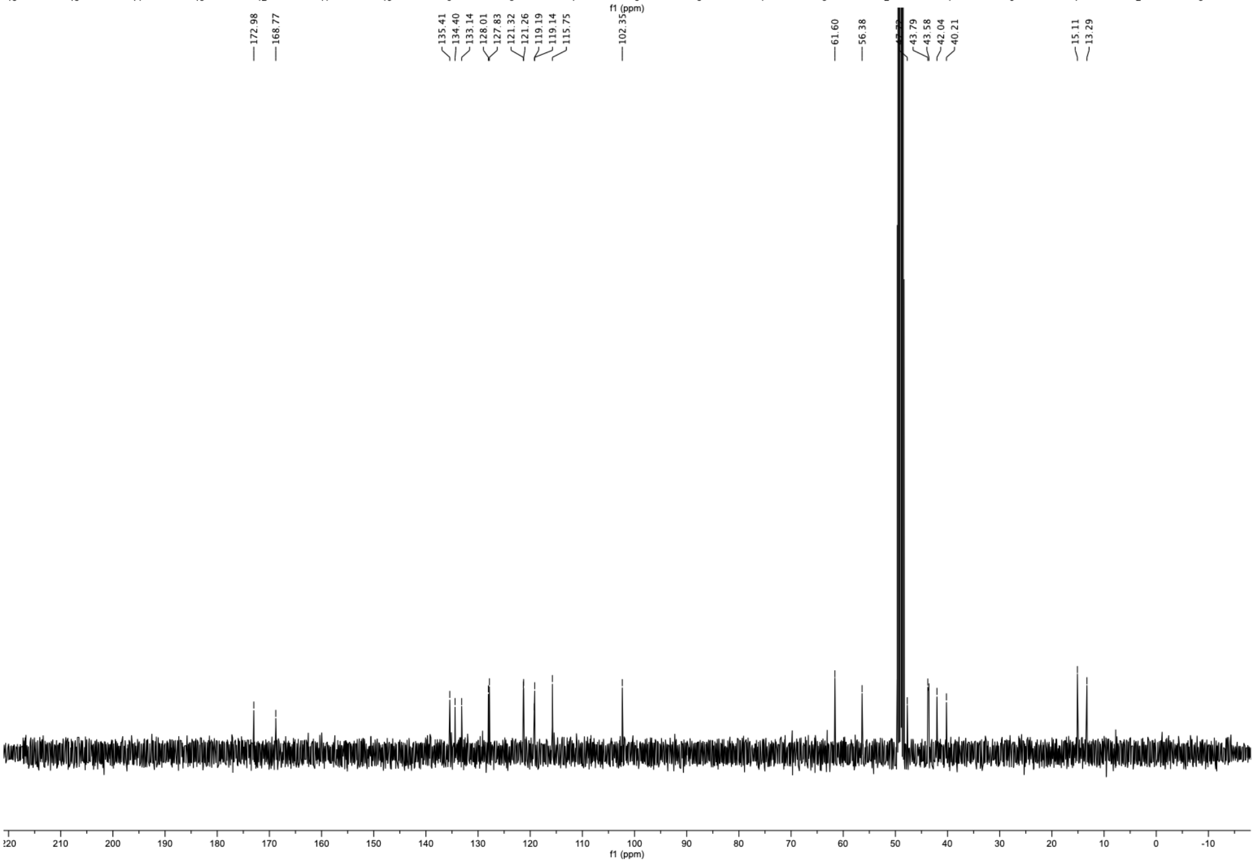

1. O. Trott, A. J. Olson, AutoDock Vina; improving the speed and accuracy of docking with a new scoring function, efficient optimization and multithreading. *J. Comput. Chem.* **31**, 455–461 (2010).
2. E. F. Petterson, T. D. Goddard, C. C. Huang, E. C. Meng, G. S. Couch, T. I. Croll, J. H. Morris, T. E. Ferrin, UCSF ChimeraX: structure visualization for researchers, educators, and developers. *Protein Sci.* 2021, **30**, 70 – 82.
3. T.D. Goddard, C. C. Huang, E. C. Meng, E. F. Petterson, G. S. Couch, J. H. Morris, T. E. Ferrin, UCSF ChimeraX: meeting modern challenges in visualization and analysis. *Protein Sci.* 2018, **27**, 14 – 25.
4. T. N. H. Pham, T. H. Nguyen, N. M. Tam, T. Y. Vu, N. T. Pham, N. T. Huy, B. K. Mai, N. T. Tung, M. Q. Pham, V. V. Vu, S. T. Ngo, Improving ligand-ranking of AutoDock Vina by changing the empirical parameters. *J. Comput. Chem.* **43**, 160–169 (2022).
5. D. E. Nichols, S. Frescas, D. Marona-Lewicka, D. M. Kurrasch-Orbaugh, Lysergamides of isomeric 2,4-dimethylazetidines map the binding orientation of the diethylamide moiety in the potent hallucinogenic agent N,N-diethyllysergamide (LSD). *J. Med. Chem.* **45**, 4344–4349 (2002).
6. S. J. Peroutka, Pharmacological differentiation and characterization of 5-HT1A, 5-HT1B, and 5-HT1C binding sites in rat frontal cortex. *J. Neurochem.* **47**, 529–540 (1986).
7. A. Janowsky, A. J. Eshleman, R. A. Johnson, K. M. Wolfrum, D. J. Hinrichs, J. Yang, T. M. Zabriskie, M. J. Smilkstein, M. K. Riscoe, Mefloquine and Psychotomimetics Share Neurotransmitter Receptor and Transporter Interactions In Vitro. *Psychopharmacology (Berl).* **231**, 2771–2783. (2014)
8. A. R. Knight, A. Misra, K. Quirk, K. Benwell, D. Revell, G. Kennett, M. Bickerdike, Pharmacological characterisation of the agonist radioligand binding site of 5-HT(2A), 5-HT(2B) and 5-HT(2C) receptors. *Naunyn Schmiedebergs Arch Pharmacol.* **370**, 114–123 (2004).
9. F. G. Boess, F. J. Monsma Jr., C. Carolo, V. Meyer, A. Rudler, C. Zwingelstein, A. J. Sleight, Functional and radioligand binding characterization of rat 5-HT6 receptors stably expressed in HEK293 cells. *Neuropharmacology* **36**, 713–720 (1997).
10. D. W. Hirst,B. Abrahamsen, F. E. Blaney, A. R. Calver, L. Aloi, G. W. Price, A. D. Medhurst, Differences in the central nervous system distribution and pharmacology of the mouse 5-hydroxytryptamine-6 receptor compared with rat and human receptors investigated by radioligand binding, site-directed mutagenesis, and molecular modeling. *Mol. Pharmacol.* **64**, 1295–1308 (2004).
11. F. G. Boess, I. L. Martin, Molecular biology of 5-HT receptors. *Neuropharmacology* **33**, 275–317 (1994).
12. Rickli, D. Luethi, J. Reinisch, D. Buchy, M. C. Hoener, M. E. Liechti, Receptor interaction profiles of novel N-2-methoxybenzyl (NBOMe) derivatives of 2,5-dimethoxy-substituted phenethylamines (2C drugs). *Neuropharmacology* **99**, 546–553 (2015).
13. D. S. Choi, G. Birraux, J. M. Launay, L. Maroteaux The human serotonin 5-HT2B receptor: pharmacological link between 5-HT2 and 5-HT1D receptors. *FEBS Lett.* **352**, 393–399 (1994).
14. R. H. Porter, K. R. Benwell, H. Lamb, C. S. Malcolm, N. H. Allen, D. F. Revell, D. R. Adams, M. J. Sheardown. Functional characterization of agonists at recombinant human 5-HT2A, 5-HT2B and 5-HT2C receptors in CHO-K1 cells. *Br. J. Pharmacol.* **128**, 13–20 (1999).
15. C. Dong, C. Ly, L. E. Dunlap, M. V. Vargas, J. Sun, I. Hwang, A. Azinfar, W. C. Oh, W. C. Wetsel, D. E. Olson, L. Tian, Psychedelic-inspired drug discovery using an engineered biosensor. *Cell* **184**, 2779–2792.e18 (2021).
16. D. Winpenny, M. Clark, D. Cawkill, Biased ligand quantification in drug discovery: From theory to high throughput screening to identify new biased μ opioid receptor agonists. *Br. J. Pharmacol.* **173**, 1393–1403 (2016).
17. L. E. Dunlap, A. Azinfar, C. Ly, L. P. Cameron, J. Viswanathan, R. J. Tombari, D. Myers-Turnbull, J. C. Taylor, A. C. Grodzki, P. J. Lein, D. Kokel, D. E. Olson, Identification of pyschoplastogenic N,N-dimethylaminoisotryptamine (isoDMT) analogues through structure-activity relationship studies. *J. Med. Chem.* **63**, 1142–1155 (2020).
18. C. Ly, A. C. Greb, L. P. Cameron, J. M. Wong, E. V. Barragan, P. C. Wilson, K. F. Burbach, S. S. Zarandi, A. Sood, M. R. Paddy, W. C. Duim, M. Y. Dennis, A. K. McAllister, K. M. Ori-McKinney, J. A. Gray, D. E. Olson, Psychedelics promote structural and functional neural plasticity. *Cell Rep.* **23**, 3170–3182 (2018).
19. M. K. St-Pierre, M. Bordeleau, M. È. Tremblay, Visualizing dark microglia. *Methods Mol. Biol.* **2034**, 97–110 (2019).
20. H. Horstmann, C. Körber, K. Sätzler, D. Aydin, T. Kuner, Serial section scanning electron microscopy (S^3EM) on silicon wafers for ultra-structural volume imaging of cells and tissues. *PLoS One* **7**, e35172 (2012).
21. A. Burel, M. Lavault, C. Chevalier, H. Gnaegi, S. Prigent, A. Mucciolo, S. Dutertre, B. M. Humberl, T. Guillaudeux, A targeted 3D EM and correlative microscopy method using SEM array tomography. *Development* **145**, dev160879 (2018).
22. M. Kuwajima, J. M. Mendenhall, L. F. Lindsey, K. M. Harris, Automated transmission-mode scanning electron microscopy (tSEM) for large volume analysis at nanoscale resolution. *PLoS One* **8**, e59573 (2013).

23. A. Cardona, S. Saalfeld, J. Schindelin, I. Arganda-Carreras, S. Preibisch, M. Longair, P, Tomancak, V. Hartenstein, R. J. Douglas, TrakEM2 software for neural circuit reconstruction. *PLoS One* **7**, e38011 (2012).
24. A. W. Wetzel, J. Bakal, M. Dittrich, D. G. C. Hildebrand, J. L. Morgan, J. W. Lichtman, Registering large volume serial-section electron microscopy image sets for neural circuit reconstruction using FFT signal whitening. *IEEE Applied Imagery Pattern Recognition Workshop (AIPR)*, 1–10, 10.1109/AIPR.2016.8010595 (2016).
25. E. Litvina, A. Adams, A. Barth, M. Bruchez, J. Carson, J. E. Chung, K. B. Dupre, L. M. Frank, K. M. Gates, K. M. Harris, H. Joo, J. W. Lichtman, K. M. Ramos, T. Sejnowski, J. S. Trimmer, S. White, W. Koroshetz, BRAIN initiative: Cutting-edge tools and resources for the community. *J. Neurosci.* **39**, 8275–8284 (2019).
26. D. R. Berger, H. S. Seung, J. W. Lichtman, VAST (volume annotation and segmentation tool): Efficient manual and semi-automatic labeling of large 3D image stacks. *Front. Neural Circuits* **12**, 1–15 (2018).
27. Blender Contributors (2017). Blender - a 3D modelling and rendering package. Stichting Blender Foundation, Amsterdam. Retrieved from http://www.blender.org.
28. K. M. Harris, R. J. Weinberg, Ultrastructure of synapses in the mammalian brain. *Cold Spring Harb. Perspect. Biol.* **4**, a005587 (2012).
29. D. A. Smith, J. M. Bailey, D. Williams, W. E. Fantegrossi, Tolerance and cross-tolerance to head twitch behavior elicited by phenethylamine- and tryptamine-derived hallucinogens in mice. *J. Pharmacol. Exp. Ther.* **351**, 485–491 (2014).
30. M. J. Gandal, P. Zhang, E. Hadjimichael, R. L. Walker, C. Chen, S. Liu, H. Won, H. van Bakel, M. Varghese, Y. Wang, A. W. Shieh, J. Haney, S. Parhami, J. Belmont, M. Kim, P. M. Losada, Z. Khan, J. Mleczko, Y. Xia, R. Dai, D. Wang, Y. T. Yang, M. Xu, K. Fish, P. R. Hof, J. Warrell, D. Fitzgerald, K. White, A. E. Jaffe; PsychENCODE Consortium; M. A. Peters, M. Gerstein, C. Liu, L. M. Iakoucheva, D. Pinto, D. H. Geschwind. Transcriptome-wide isoform-level dysregulation in ASD, schizophrenia, and bipolar disorder. *Science* **362**, eaat8127 (2018).
31. B. D. Kangas, J. Bergman, Touchscreen technology in the study of cognition-related behavior. *Behav. Pharmacol.* **28**, 623–629 (2017).
32. B. D. Kangas, L. M. Wooldridge, O. T. Luc, J. Bergman, D. A. Pizzagalli, Empirical validation of a touchscreen probabilistic reward task in rats. *Transl Psychiatry.* **10**, 285 (2020).
33. D. A. Pizzagalli, A. L. Jahn, J. P. O'Shea, Toward an objective characterization of an anhedonic phenotype: a signal-detection approach. *Biol Psychiatry* **57**, 319–327 (2005).
34. O. T. Luc, D. A. Pizzagalli, B. D. Kangas, Toward a Quantification of Anhedonia: Unified Matching Law and Signal Detection for Clinical Assessment and Drug Development. *Perspect Behav Sci* **44**, 517–540 (2021).
35. J. Lu, M. Tjia, B. Mullen, B. Cao, K. Lukasiewicz, S. Shah-Morales, S. Weiser, L. P. Cameron, D. E. Olson, L. Chen, Y. Zuo, An Analog of Psychedelics Restores Functional Neural Circuits Disrupted by Unpredictable Stress. *Mol. Psychiatry* **26**, 6237–6252 (2021).
36. C. Johnson, L. Wilbrecht, Juvenile mice show greater flexibility in multiple choice reversal learning than adults. *Dev. Cogn. Neurosci.* **1**, 540–551 (2011).
37. A. Harkin, D. D. Houlihan, J. P. Kelly, Reduction in preference for saccharin by repeated unpredictable stress in mice and its prevention by imipramine. *J Psychopharmacol.* **16**, 115–123 (2002).
38. M. C. Schweizer, M. S. Henniger, I. Sillaber, Chronic mild stress (CMS) in mice: of anhedonia, 'anomalous anxiolysis' and activity. *PLoS One* **4**, e4326 (2009).
39. Bruker (2019) APEXII (Version 2019.0) and (2016) SAINT (Version 8.37a). Bruker AXS Inc., Madison, Wisconsin, USA.
40. R. H. Blessing, An empirical correction for absorption anisotropy. *Acta Crystallogr. A.* **51**, 33-38 (1995).
41. G. M. Sheldrick, SADABS (2016) Version 2016/2, 'Siemens Area Detector Absorption Correction' Universität Göttingen: Göttingen, Germany.
42. G. M. Sheldrick, (2002). SHELXTL. Version 6.1. Bruker AXS Inc., Madison, Wisconsin, USA.
43. G. M. Sheldrick, (2015) SHELXT – Integrated space-group and crystal-structure determination. *Acta. Cryst.* **A71**, 3–8. (2015)
44. G. M. Sheldrick, (2018). SHELXL2018/3. Universität Göttingen: Göttingen, Germany.
45. H. D. Flack, On enantiomorph-polarity estimation. *Acta Crystallogr. A.* **39**, 876-881 (1983).
46. R. W. W. Hooft, L. H. Straver, A. L. Spek, Determination of absolute structure using Bayesian statistics on bijvoet differences. J. *Appl, Crystallogr.* **41**, 96-103 (2008).
47. A. L. Thompson, D. J. Watkin, X-ray crystallography and chirality: understanding the limitations. *Tetrahedron Asymmetry* **20**, 712–717 (2009).
